# On the Role of Pre- and Post-Electron-Transfer Steps in the SmI_2_/Amine/H_2_O-Mediated Reduction of Esters: New Mechanistic Insights and Kinetic Studies

**DOI:** 10.1002/chem.201400295

**Published:** 2014-03-11

**Authors:** Michal Szostak, Malcolm Spain, David J Procter

**Affiliations:** [a] School of Chemistry, University of ManchesterOxford Road, Manchester M13 9PL (UK), Fax: (+44) 161-275-4939

**Keywords:** electron donors, electron transfer, radicals, reduction, reductive coupling

## Abstract

The mechanism of the SmI_2_-mediated reduction of unactivated esters has been studied using a combination of kinetic, radical clocks and reactivity experiments. The kinetic data indicate that all reaction components (SmI_2_, amine, H_2_O) are involved in the rate equation and that electron transfer is facilitated by Brønsted base assisted deprotonation of water in the transition state. The use of validated cyclopropyl-containing radical clocks demonstrates that the reaction occurs via fast, reversible first electron transfer, and that the electron transfer from simple Sm(II) complexes to aliphatic esters is rapid. Notably, the mechanistic details presented herein indicate that complexation between SmI_2_, H_2_O and amines affords a new class of structurally diverse, thermodynamically powerful reductants for efficient electron transfer to carboxylic acid derivatives as an attractive alternative to the classical hydride-mediated reductions and as a source of acyl-radical equivalents for C=C bond forming processes.

Samarium(II)-mediated generation of ketyl radicals has been the focus of intense research for more than three decades,[1] and the SmI_2_-promoted reductions, which enable the synthesis of alcohols under conditions orthogonal to other reagents operating through single- and two-electron pathways,[2,3] are a prominent class of these processes (Figure 1). Until recently, it had been thought that unactivated carboxylic acid derivatives were outside the reducing range of SmI_2_,[4] which prevented progression of the rich carbonyl chemistry of SmI_2_ (e.g., reduction, cross-coupling, tandem bond-forming events) to acyl-type radicals generated from carboxylic acid derivatives under mild and chemoselective reaction conditions (Figure 1).

**Figure 1:**
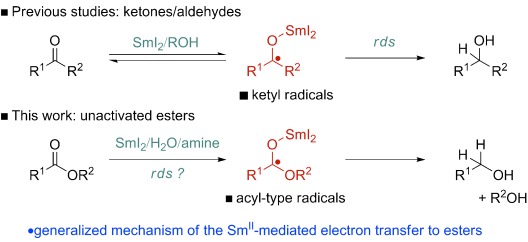
Accepted and proposed mechanism of SmI_2_-mediated electron transfer to aldehydes, ketones, and carboxylic acid derivatives; rds=rate-determining step.

In 2011, we reported that the Sm^II^ reagent produced from SmI_2_, amine and H_2_O is capable of reducing unactivated esters via radical intermediates,[5] thus for the first time expanding the carbonyl chemistry of SmI_2_ beyond ketones and aldehydes.[6] However, the mechanistic details of this process, including the critical role of amine and H_2_O additives, remained unclear.[6,7] As a better mechanistic understanding of the role of these additives could afford key insights for the development of new reductive processes, including chemoselective reduction of less reactive functional groups, such as nitriles, amides and amino acids, as well as the development of new C=C bond-forming reactions,[8] we initiated a mechanistic investigation into the reduction of unactivated esters using SmI_2_/amine/H_2_O. The data described herein show two important features: 1) all reaction components (SmI_2_, amine, H_2_O) are involved in the rate equation , and there is a direct correlation between the rate of ester reduction and p*K*_BH+_ of amines; 2) the reaction occurs via fast, reversible first electron transfer, and the electron transfer from simple Sm^II^ complexes to aliphatic esters is rapid. Importantly, this study sets the stage for the use of SmI_2_/amine/H_2_O complexes to generate acyl-type radicals from a plethora of carboxylic acid derivatives.

We started our investigation by conducting a range of kinetic studies (Table 1). *tert*-Butyl 3-phenylpropanoate (**1**) was selected as a model substrate, because its rate of reduction is in a convenient range for kinetic studies, and there is ample literature precedent for Sm^II^ reduction conditions available for this substrate.[5] Within experimental error, the reduction of **1** in the presence of SmI_2_/Et_3_N/H_2_O was found to be first order in all components of the reaction (Table 1). The rate constant of 1.4±0.1×10 m^−3^ s^−1^ was determined for the reduction of **1** under these reaction conditions. Taken together, these results suggest that all reaction components are involved in the rate equation, and that the reduction of **1** is a fast process.

**Table 1:** Rate constant and reaction orders for the reduction of 1 using the SmI_2_/Et_3_N/H_2_O system.[a]

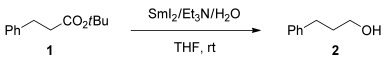				
	Rate order			
*k*[a][m^−3^ s^−1^]	Substrate[a]	SmI_2_[b]	Et_3_N[c]	H_2_O[d]
1.4×10	0.96±0.10	1.09±0.10	1.18±0.10	0.92±0.10

[a] [SmI_2_]=75 mm; [H_2_O]=250 mm; [Et_3_N]=150 mm; [ester]=5–20 mm.

[b] [SmI_2_]=50–100 mm; [H_2_O]=250 mm; [Et_3_N]=150 mm; [ester]=12.5 mm.

[c] [SmI_2_]=75 mm; [H_2_O]=250 mm; [Et_3_N]=75–250 mm; [ester]=12.5 mm.

[d] [SmI_2_]=75 mm; [H_2_O]=75–300 mm; [Et_3_N]=150 mm; [ester]=12.5 mm. *T*=23 °C. See the Supporting Information.

To further explore the impact of H_2_O, the reduction rate of **1** was monitored over a 20-fold concentration range as depicted in Figure 2. In this study, a nonlinear rate dependence on H_2_O was found. At lower concentrations (up to 300 mm), the rate was found to increase linearly with a slope corresponding to the rate order of one, consistent with saturation behavior (300 mm). However, at higher concentrations (300–1200 mm), the rate decreased dramatically, consistent with substrate displacement from the inner coordination sphere of Sm^II^. In contrast, a linear rate dependence on amine at these concentrations was found. In agreement with previous studies, H_2_O is expected to show high affinity for Sm^II^ and compete for coordination to Sm^II^ with the ester substrate.[9] Interestingly, the concentration of H_2_O at which the decrease in the reaction rate was observed correlates with iodide displacement from the Sm^II^ coordination sphere.[10]

**Figure 2:**
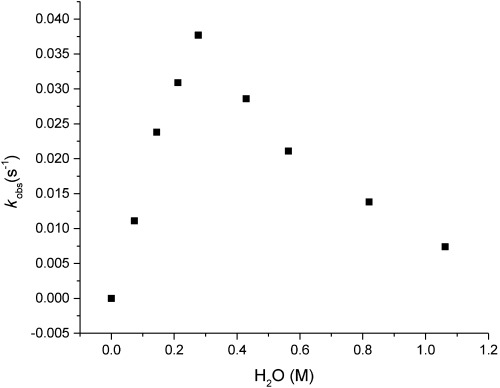
Plot of *k*_obs_ versus concentration of H_2_O for the reduction of 1. [H_2_O]=0.075–1.2 m; [SmI_2_]=75 mm; [Et_3_N]=150 mm; [ester]=12.5 mm; *T*=23 °C.

To further elucidate the role of the amine component, the reduction rate of **1** was measured in the presence of a wide range of amines with varying steric and electronic properties (Table 2). Remarkably, a dramatic change in the reaction rate of over two orders of magnitude was found by simply using different amines for the reduction. Moreover, a good correlation between the reaction rate and basicity of amines was obtained.[11] By plotting log (*k*_obs_) versus p*K*_BH+_, a linear correlation was found with a slope corresponding to 0.79, which corresponds very well to the value obtained in the reduction of alkyl halides via an *outer-sphere mechanism* using SmI_2_/amine/H_2_O reported by Hilmersson (0.76; for a detailed comparison, see the Supporting Information).[7e] This result strongly suggests that the role of the amine component is independent of the mechanistic pathway (inner- vs. outer-sphere electron transfer) and the relative redox potentials of both classes of substrates. Considering steric properties exerted by these amines, our findings bode well for the chemoselective fine tuning of Sm^II^/amine reductants to specific functional groups.

**Table 2:** Determined initial rate in the reduction of 1 using SmI_2_/amine/H_2_O versus p*K*_BH+_.[a]

Entry	Amine	*v*_initial_ [mM s^−1^]	p*K*_BH+_[b]
1	morpholine	2.4×10^−4^	9.0±0.2
2	*n*Bu_3_N	3.9×10^−5^	10.0±0.5
3	Et_3_N	5.0×10^−4^	10.6±0.3
4	*n*BuNH_2_	6.8×10^−3^	10.7±0.1
5	pyrrolidine	8.8×10^−3^	11.3±0.2

[a] [SmI_2_]=75 mm; [H_2_O]=250 mm; [ester]=12.5 mm; [amine]=150 mm; *T*=23 °C.

[b] Determined from ACD lab prediction algorithm.

Several additional studies give insight into the electron-transfer steps. 1) The reduction with SmI_2_/amine/D_2_O gives the alcohol with >95 % [D]_2_ incorporation suggesting that anions are protonated in a series of electron transfers. 2) The kinetic isotope effect in the reduction of isopropyl 3-phenylpropanoate using SmI_2_/Et_3_N/H_2_O of 1.5±0.1, parallel runs, and 1.4±0.1, intramolecular competition,[5] indicate that proton transfer is not involved in the rate-determining step.[12] 3) UV/Vis spectrophotometric studies carried out on various SmI_2_/amine/H_2_O systems show isosbestic points and absorbance changes upon addition of amines and H_2_O to SmI_2_,[7d] which is consistent with the formation of distinct Sm^II^ reductants.

Next, we utilized intermolecular competition studies to elucidate the actual productivity difference in the SmI_2_/amine/H_2_O-mediated reduction of esters (Table 3). In these experiments, an equimolar amount of two esters was reacted with limiting SmI_2_ (typically, less than 2 equiv). The relative reactivity values were determined from the product distribution. This method allows to accurately measure the relative reactivity values of Sm^II^-mediated reactions provided that the studied substrates do not participate in alternative reaction pathways.[13] Methyl decanoate was chosen as an arbitrary standard. Remarkably, in the series of eight methyl esters, a reactivity range of over three orders of magnitude was observed, depending on the steric and electronic properties of the α-carbon substituent at the ester group undergoing the reduction (Table 3, entries 1–8). This effect is consistent with both electronic stabilization of ketyl-type radicals (Table 3, entries 1–4) and steric inhibition of coordination to Sm^II^ (entries 4–8). Moreover, several substrates with enhanced leaving-group ability compared to the methyl ester were examined (Table 3, entries 9–12). These results further support the importance of electronic effects for the stabilization of the ketyl-type radical intermediates and determining the redox potential of the substrates.[14] Importantly, the data presented in Table 3 indicate high levels of chemoselectivity in the reduction of esters with SmI_2_/Et_3_N/H_2_O.

**Table 3:** Steric and electronic influence on the relative rates for the reduction of esters.

Entry	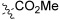	RV[a]
1	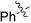	>100
2	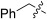	9.14
3	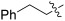	4.29
4	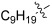	1.00
5	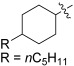	0.41
6	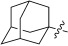	0.26
7	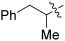	0.91
8	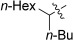	0.05

Entry	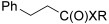	RV[a]
9	R=OMe	1.00
10	R=OPh	6.88
11	R=Opfp	9.15
12	R=SEt	5.78

[a] Relative reactivity values (RV) determined from product distribution by ^1^H NMR and/or GC analyses of crude reaction mixtures. All data represent the average of at least two experiments. pfp=pentafluorophenyl.

Evidence for the electronic and steric stabilization of ketyl-type radical intermediates was further substantiated by Hammett and Taft correlation studies (see the Supporting Information). The Hammett correlation study, employing methyl esters of 4-substituted phenylacetic acids,[15] showed a large positive *ρ* value of 0.43 (*R*^2^=0.98), which can be compared with the *ρ* value of 0.49 for ionization of phenylacetic acids in H_2_O at 25 °C.[16] The Taft correlation study,[17] obtained by plotting log (*k*_obs_) versus *E*_S_ in a series of aliphatic esters of hydrocinnamic acid showed a large positive slope of 0.97 (*R*^2^=0.97). Overall, these results suggest that an anionic intermediate is formed in the transition state of the reaction, and that a conformational change similar in geometry to the ester hydrolysis, tetrahedral intermediate, is taking place in the rate-determining step of the reaction.[18]

Finally, to gain independent evidence on the role of electron-transfer steps, we carried out several studies employing mechanistic probes (Scheme 1 and the Supporting Information). Most importantly, we recognized that implementation of a suitable radical clock should allow the detection of reversible reaction pathways.[19] To this end, the *trans*-cyclopropane-containing radical clock **3** (approximated unimolecular rate constant *k*_frag_≈3×10^11^ s^−1^ at 25 °C)[20] was selected and subjected to the reaction conditions with a limiting amount of SmI_2_ (Scheme 1). The reaction resulted in rapid cyclopropyl-ring opening to give acyclic ester **4** and alcohol **5** in 94:6 ratio. Cyclopropylcarbinol **6** was not detected in the reaction. Several control experiments were performed (see the Supporting Information). 1) The reaction of **3** with SmI_2_/H_2_O (8 equiv, RT, 2 h) resulted in a facile opening to ester **4**, with no over-reduction to **5** or **6** observed. 2) The reduction of the methyl ester of cyclopropanecarboxylic acid (approximated unimolecular rate constant *k*_frag_≈9.4×10^7^ s^−1^ at 25 °C)[20] with excess SmI_2_/amine/H_2_O afforded the corresponding acyclic alcohol and cyclopropylcarbinol in 96:4 ratio. This allows to estimate the rate of reduction of ketyl-type radicals with Sm^II^ to be comparable to a unimolecular reaction with *k* of about 10^8^ s^−1^.[21] 3) The reductive opening of radical clock **3** was not observed with other Sm^II^ reagents, including systems with higher redox potential (SmI_2_/MeOH, SmI_2_/LiCl, SmI_2_/HMPA (HMPA=hexamethylphosphoramide), and SmI_2_/Et_3_N).[4b] Finally, experiments utilizing chiral probe **7** (Scheme 1) were carried out and demonstrate that enolization did not occur in the process despite basic reaction conditions, whereas control experiments using H_2_^18^O (Scheme 1 and the Supporting Information) show that the reduction did not proceed via a sequential ester hydrolysis/acid reduction mechanism. Overall, these findings strongly suggest that the reduction of unactivated esters with SmI_2_/amine/H_2_O occurs through fast, reversible electron transfer, and, in contrary to the current paradigm,[1,2] show that electron transfer from simple SmI_2_/H_2_O complexes to aliphatic esters is rapid.[22]

**Scheme 1:**
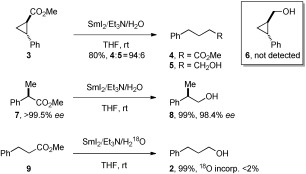
Studies designed to investigate the mechanism of reduction of unactivated esters using SmI_2_/Et_3_N/H_2_O.

A mechanism that best fits the kinetic and reactivity studies presented herein features the following steps (Scheme 2):[24] 1) Formation of the reactive complex between SmI_2_, H_2_O, and amine, in which one or more molecules of H_2_O and amine are coordinated to the Sm^II^ center.[23] Within this complex, one molecule of amine participates in partial deprotonation of H_2_O, resulting in a formal negative charge at oxygen and an overall increase of the redox potential of the Sm^II^ reductant in the transition state; 2) reversible ester coordination, protonation and first electron transfer steps; 3) rate-limiting second electron-transfer step; 4) inner-sphere electron-transfer process that is inhibited by large concentrations of H_2_O and facilitated by Brønsted basic amines; and 5) rate-determining step that can be fine-tuned by steric and electronic properties of the ester substrate. The formation of a partial negative charge at oxygen was further supported by our finding that under optimized reaction conditions, SmI_2_/NaOH/H_2_O[25] reduces aliphatic esters in high yield. From a practical point of view,[26] the p*K*_BH+_-dependent elongation of the hydrogen bond from H_2_O in SmI_2_/amine/H_2_O complexes can have a profound impact on the chemoselectivity of electron transfer to carboxylic acid derivatives.

**Scheme 2:**
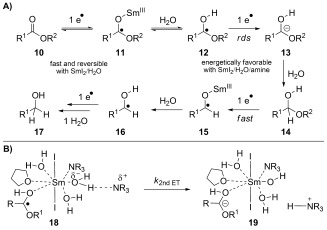
A) Proposed mechanism. B) Final steps of the reduction of esters using SmI_2_/Et_3_N/H_2_O.

In summary, we have presented a series of kinetic and reactivity experiments that probe the mechanism of the reduction of unactivated esters by using SmI_2_/amine/H_2_O. Our data are consistent with the formation of distinct Sm^II^ reductants by complexation between Sm^II^, amine, and H_2_O. The ester reduction appears to proceed after deprotonation of a molecule of H_2_O by amine and to involve a reversible first electron-transfer step. Most crucially, our results demonstrate that a set of new Sm^II^ reductants that can be fine-tuned by the p*K*_BH+_ of the amine component is now available for challenging electron-transfer reactions to carboxylic acid derivatives. Equally importantly, this work shows that the major role of additives (e.g., H_2_O, amine/H_2_O) is to stabilize the ketyl intermediates. We fully expect that these findings will serve as a foundation to enable the development of new electron-transfer reactions. Work in this direction using Sm^II^ systems is ongoing in our laboratories, and these results will be reported shortly.
