# Supplementary material for: On the Role of Pre- and Post-Electron-Transfer Steps in the SmI2/Amine/H2O-Mediated Reduction of Esters: New Mechanistic Insights and Kinetic Studies
Source: Chemistry. 2014 Mar 11;20(15):4222–6. doi: 10.1002/chem.201400295 (PMC4497321; doi:10.1002/chem.201400295)
Supplement: Supplementary file 1 [file chem0020-4222-sd1.pdf]

CHEMISTRY
A European Journal

Supporting Information

© Copyright Wiley-VCH Verlag GmbH & Co. KGaA, 69451 Weinheim, 2014

On the Role of Pre- and Post-Electron-Transfer Steps in the SmI_2/amine/H_2O-Mediated Reduction of Esters: New Mechanistic Insights and Kinetic Studies

Michal Szostak,* Malcolm Spain, and David J. Procter*[a]

chem_201400295_sm_miscellaneous_information.pdf

On the Role of Pre- and Post-Electron Transfer Steps in the SmI_2/amine/H_2O-Mediated Reduction of Esters: New Mechanistic Insights and Kinetic Studies

Michal Szostak,* Malcolm Spain, and David J. Procter*

School of Chemistry, University of Manchester, Oxford Road, Manchester, M13 9PL, UK

michal.szostak@manchester.ac.uk; david.j.procter@manchester.ac.uk

Supplementary Information

Table of Contents 1

List of Known Compounds/General Methods 2
Experimental Procedures and Characterization Data 2

- Intermolecular Competition Experiments 2
- Hammett and Taft Studies 7
- Radical Clock Experiments 12
- Epimerization Studies 18
- $H_2{}^{18}O$ Incorporation Experiments 19
- Reagent Stoichiometry Studies 20
- Reduction of Esters using SmI_2–NaOH 21

Kinetic Experiments 23
References 27
^{1}H and ^{13}C NMR Spectra 28
HPLC Traces 66

Corresponding Author:

Dr. Michal Szostak
Professor David J. Procter
School of Chemistry
University of Manchester
Oxford Road
Manchester, M13 9PL
United Kingdom

General Methods/List of Known Compounds

All compounds reported in the manuscript study have been described in literature or are commercially available. Esters were purchased from commercial suppliers or prepared by standard methods.[1-9] All experiments involving SmI_2 were performed using standard Schlenk or glovebox techniques under argon or nitrogen atmosphere unless stated otherwise. All solvents were purchased at the highest commercial grade and used as received or after purification by passing through activated alumina columns or distillation from sodium/benzophenone under nitrogen. All solvents were deoxygenated prior to use. All other chemicals were purchased at the highest commercial grade and used as received. Reaction glassware was oven-dried at 140 °C for at least 24 h or flame-dried prior to use, allowed to cool under vacuum and purged with argon (three cycles). Samarium(II) iodide was prepared by standard methods and titrated prior to use.[10-14] ^{1}H NMR and ^{13}C NMR spectra were recorded in $CDCl_3$ on Bruker spectrometers at 300, 400 and 500 MHz (^{1}H NMR) and 75, 100 and 125 MHz (^{13}C NMR). All shifts are reported in parts per million (ppm) relative to residual $CHCl_3$ peak (7.27 and 77.2 ppm, ^{1}H NMR and ^{13}C NMR, respectively). All coupling constants (J) are reported in hertz (Hz). Abbreviations are: s, singlet; d, doublet; t, triplet; q, quartet; br s, broad singlet. All flash chromatography was performed using silica gel, 60 Å, 230−400 mesh. TLC analysis was carried out on aluminium sheets coated with silica gel 60 F254, 0.2 mm thickness. The plates were visualized using a 254 nm ultraviolet lamp or aqueous potassium permanganate solutions. ^{1}H NMR and ^{13}C NMR data are given for all compounds in the Supporting Experimental for characterization purposes. ^{1}H NMR, ^{13}C NMR, IR and HRMS data are reported for all new compounds.

Intermolecular Competition Experiments

General Procedure. An oven-dried vial containing a stir bar was placed under a positive pressure of argon, and subjected to three evacuation/backfilling cycles under high vacuum. Samarium(II) iodide (THF solution, typically 0.20 mmol, 2.0 equiv, 0.10 M) was added followed by Et_3N (0.33 mL, 24 equiv) and H_2O (0.043 mL, 24 equiv) with vigorous stirring, which resulted in the formation of a characteristic dark brown color of the SmI_2–Et_3N–H_2O complex. A preformed solution of two substrates (each 0.10 mmol, 1.0 equiv, stock solution in THF, 1.0 mL) was added and the reaction mixture was stirred until decolorization to white had occurred. The reaction mixture was diluted with CH_2Cl_2 (30 mL) and HCl (1 *N*, 30 mL).

The aqueous layer was extracted with CH_2Cl_2 (3 x 30 mL), the organic layers were combined, dried over Na_2SO_4, filtered, and concentrated. The sample was analyzed by ^1H NMR ($CDCl_3$, 500 MHz) and GC-MS to obtain conversion and yield using internal standard and comparison with authentic samples.

Table SI-1. Steric and Electronic Influence on the Relative Rates in the Reduction of Esters.[a]

entry	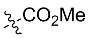	Run 1[b]	Run 2[b]	RV[b]
1	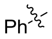	99.1:0.9	99.1:0.9	>100
2	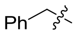	90:10	90:10	9.14
3	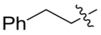	81:19	81:19	4.29
4	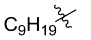	1.00	1.00	1.00
5	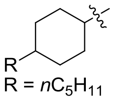	29:71	29:71	0.41
6	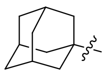	20:80	21:79	0.26
7	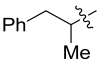	49:51	46:54	0.91
8	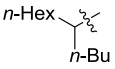	4.4:95.6	5.3:94.7	0.05
entry	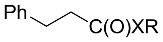	Run 1[b]	Run 2[b]	RV[b]
9	R = OMe	1.00	1.00	1.00
10	R = OPh	87:13	87:13	6.88
11	R = Opfp	90:10	90:10	9.15
12	R = SEt	85:15	85:15	5.78

[a]All reactions carried out using standard Schlenk techniques. Conditions: under argon, to SmI_2 (typically, 0.20 mmol), Et_3N (typically, 2.4 mmol) and H_2O (typically 2.4 mmol) an equimolar amount of the competition substrates (typically, 0.10 mmol) in THF (typically, 1 mL) was added, and the reaction mixture was vigorously stirred until decolorization to white had occurred. [b]Relative reactivity values determined from product distribution by ^1H NMR and/or GC of crude reaction mixtures and comparison with authentic samples. All data represent average of at least two experiments.

Methyl benzoate (Table 3, entry 1). ^1H NMR (500 MHz, $CDCl_3$) δ 3.83 (s, 3 H), 7.35 (t, *J* = 7.5 Hz, 2 H), 7.46 (tt, *J* = 1.5, 8.0 Hz, 1 H), 7.95 (dd, *J* = 1.5, 8.5 Hz, 2 H); ^{13}C NMR (125 MHz, $CDCl_3$) δ 52.1, 128.4, 129.6, 130.2, 132.9, 167.1.

Methyl 2-phenylacetate (Table 3, entry 2). ^1H NMR (500 MHz, $CDCl_3$) δ 3.53 (s, 2 H), 3.59 (s, 3 H); 7.14-7.20 (m, 3 H), 7.21-7.25 (m, 2 H); ^{13}C NMR (125 MHz, $CDCl_3$) δ 41.2, 52.1, 127.1, 128.6, 129.3, 134.0, 172.1.

Methyl 3-phenylpropanoate (Table 3, entries 3, 9). ^{1}H NMR (300 MHz, $CDCl_3$) δ 2.68 (t, *J* = 7.8 Hz, 2 H), 3.00 (t, *J* = 7.5 Hz, 2 H), 3.71 (s, 3 H), 7.21-7.28 (m, 3 H), 7.30-7.37 (m, 2 H); ^{13}C NMR (75 MHz, $CDCl_3$) δ 31.0, 35.7, 51.6, 126.3, 128.3, 128.6, 140.6, 173.3.

Methyl decanoate (Table 3, entry 4). ^{1}H NMR (300 MHz, $CDCl_3$) δ 0.84 (t, *J* = 6.6 Hz, 3 H), 1.18-1.31 (m, 12 H), 1.52-1.64 (m, 2 H), 2.27 (t, *J* = 7.8 Hz, 2 H), 3.62 (s, 3 H); ^{13}C NMR (75 MHz, $CDCl_3$) δ 14.0, 22.6, 24.9, 29.1, 29.2, 29.2, 29.4, 31.8, 34.0, 51.3, 174.2.

(1*S*,4*R*)-Methyl 4-pentylcyclohexanecarboxylate (Table 3, entry 5). ^{1}H NMR (300 MHz, $CDCl_3$) δ 0.83-0.98 (m, 2 H), 0.89 (t, *J* = 6.6 Hz, 3 H), 1.13-1.34 (m, 9 H), 1.42 (qd, *J* = 3.3, 12.6 Hz, 2 H), 1.77-1.86 (m, 2 H), 1.92-2.01 (m, 2 H), 2.24 (tt, *J* = 3.6, 12.3 Hz, 1 H), 3.67 (s, 3 H); ^{13}C NMR (75 MHz, $CDCl_3$) δ 14.1, 22.7, 26.5, 29.1, 32.2, 32.4, 36.9, 37.2, 43.5, 51.4, 176.8.

Methyl adamantane-1-carboxylate (Table 3, entry 6). ^{1}H NMR (300 MHz, $CDCl_3$) δ 1.62-1.75 (m, 6 H), 1.84-1.89 (m, 6 H), 1.95-2.02 (m, 3 H), 3.63 (s, 3 H); ^{13}C NMR (75 MHz, $CDCl_3$) δ 27.9, 36.5, 38.8, 40.7, 51.5, 178.2.

Methyl 2-methyl-3-phenylpropanoate (Table 3, entry 7). ^{1}H NMR (300 MHz, $CDCl_3$) δ 1.19 (d, *J* = 6.6 Hz, 3 H), 2.65-2.84 (m, 2 H), 3.07 (q, *J* = 6.3 Hz, 1 H), 3.67 (s, 3 H), 7.17-7.22 (m, 2 H), 7.24-7.35 (m, 3 H); ^{13}C NMR (75 MHz, $CDCl_3$) δ 16.8, 39.7, 41.5, 51.6, 126.4, 128.4, 129.0, 139.4, 176.6.

Methyl 2-butyloctanoate (Table 3, entry 8). ^{1}H NMR (300 MHz, $CDCl_3$) δ 0.88 (t, *J* = 6.9, 3 H), 0.89 (t, *J* = 6.6 Hz, 3 H), 1.18-1.34 (m, 12 H), 1.38-1.51 (m, 2 H), 1.53-1.67 (m, 2 H), 2.29-2.39 (m, 1 H), 3.68 (s, 3 H); ^{13}C NMR (75 MHz, $CDCl_3$) δ 13.9, 14.0, 22.6, 22.6, 27.4, 29.2, 29.7, 31.7, 32.2, 32.5, 45.7, 51.3, 177.1.

Phenyl 3-phenylpropanoate (Table 3, entry 10). ^{1}H NMR (300 MHz, $CDCl_3$) δ 2.96 (t, *J* = 7.5 Hz, 2 H), 3.15 (t, *J* = 7.2 Hz, 2 H), 7.05-7.11 (m, 2 H), 7.24-7.46 (m, 8 H); ^{13}C NMR (75 MHz, $CDCl_3$) δ 31.0, 36.1, 121.6, 125.9, 126.5, 128.5, 128.7, 129.5, 140.2, 150.7, 171.5.

Perfluorophenyl 3-phenylpropanoate (Table 3, entry 11). ^{1}H NMR (300 MHz, $CDCl_3$) δ 2.88-2.95 (m, 2 H), 2.99-3.05 (m, 2 H), 7.14-7.21 (m, 3 H), 7.22-7.29 (m, 2 H); ^{13}C NMR (75 MHz, $CDCl_3$) δ 30.7, 25.0, 126.7, 128.3, 128.7, 139.3, 168.8; ^{19}F (376 MHz, $CDCl_3$) δ -162.3 (m, 2 F), -158.0 (t, *J* = 21.5 Hz, 1 F), -152.5 (m, 2 F). Aromatic CF groups were not apparent in the ^{13}C NMR spectrum despite long acquisition times. The ^{19}F spectrum clearly indicates the presence of the pfp group.

***S*-Ethyl 3-phenylpropanethioate (Table 3, entry 12).** ^{1}H NMR (500 MHz, $CDCl_3$) δ 1.14 (t, *J* = 7.5 Hz, 3 H), 2.75 (t, *J* = 7.0 Hz, 2 H), 2.78 (q, *J* = 7.5 Hz, 2 H), 2.88 (t, *J* = 8.0 Hz, 2 H), 7.07-7.12 (m, 3 H), 7.16-7.21 (m, 2 H); ^{13}C NMR (125 MHz, $CDCl_3$) δ 14.8, 23.4, 31.5, 45.5, 126.4, 128.4, 128.6, 140.2, 198.6.

Benzyl alcohol (Table 3, entry 1). ^{1}H NMR (500 MHz, $CDCl_3$) δ 1.75 (br, 1 H), 4.60 (s, 2 H), 7.19-7.24 (m, 1 H), 7.26-7.31 (m, 4 H); ^{13}C NMR (125 MHz, $CDCl_3$) δ 65.4, 127.0, 127.7, 128.6, 140.9.

Phenethyl alcohol (Table 3, entry 2). ^{1}H NMR (500 MHz, $CDCl_3$) δ 1.65 (br, 1 H), 2.78 (t, *J* = 7.0 Hz, 2 H), 3.76 (t, *J* = 6.5 Hz, 2 H), 7.13-7.17 (m, 3 H), 7.21-7.25 (m, 2 H); ^{13}C NMR (125 MHz, $CDCl_3$) δ 39.2, 63.7, 126.5, 128.6, 129.1, 138.5.

3-Phenylpropan-1-ol (Table 3, entries 3, 10-12). ^{1}H NMR (400 MHz, $CDCl_3$) δ 1.32 (br, 1 H), 1.79-1.87 (m, 2 H), 2.64 (t, *J* = 7.6 Hz, 2 H), 3.61 (t, *J* = 6.4 Hz, 2 H), 7.09-7.24 (m, 5 H); ^{13}C NMR (100 MHz, $CDCl_3$) δ 32.1, 34.3, 62.3, 125.9, 128.4, 128.5, 141.8.

Decan-1-ol (Table 3, entry 4). ^{1}H NMR (500 MHz, $CDCl_3$) δ 0.81 (t, *J* = 6.9 Hz, 3 H), 1.15-1.33 (m, 15 H), 1.47-1.53 (m, 2 H), 3.57 (t, *J* = 6.6 Hz, 2 H); ^{13}C NMR (125 MHz, $CDCl_3$) δ 14.1, 22.7, 25.8, 29.3, 29.5, 29.6, 29.7, 31.9, 32.8, 63.1.

((1*S*,4*R*)-4-pentylcyclohexyl)methanol (Table 3, entry 5). ^{1}H NMR (300 MHz, $CDCl_3$) δ 0.75-0.91 (m, 7 H), 1.02-1.27 (m, 10 H), 1.38 (br, 1 H), 1.71 (d, *J* = 8.7 Hz, 4 H), 3.37 (d, *J* = 6.4 Hz, 2 H); ^{13}C NMR (75 MHz, $CDCl_3$) δ 14.1, 22.7, 26.6, 29.5, 32.2, 32.7, 37.4, 37.8, 40.7, 68.8.

1-Adamantanemethanol (Table 3, entry 6). ^{1}H NMR (400 MHz, $CDCl_3$) δ 1.26 (br, 1 H), 1.43-1.46 (m, 6 H), 1.55-1.70 (m, 6 H), 1.90-1.95 (m, 3 H), 3.13 (s, 2 H); ^{13}C NMR (75 MHz, $CDCl_3$) δ 28.2, 34.5, 37.2, 39.0, 73.9.

2-Methyl-3-phenylpropan-1-ol (Table 3, entry 7). ^{1}H NMR (300 MHz, $CDCl_3$) δ 0.85 (d, J = 6.9 Hz, 3 H), 1.33 (br, 1 H), 1.79-1.94 (m, 1 H), 2.36 (dd, J = 8.0, 13.5 Hz, 1 H), 2.69 (dd, J = 6.2, 13.4 Hz, 1 H), 3.37-3.50 (m, 2 H), 7.07-7.25 (m, 5 H); ^{13}C NMR (75 MHz, $CDCl_3$) δ 16.5, 37.8, 39.7, 67.7, 125.9, 128.3, 129.2, 140.6.

2-Butyloctan-1-ol (Table 3, entry 8). ^{1}H NMR (500 MHz, $CDCl_3$) δ 0.79-0.85 (m, 6 H), 1.09-1.14 (br, 1 H), 1.16-1.29 (m, 16 H), 1.35-1.42 (m, 1 H), 3.47 (d, J = 5.5 Hz, 2 H); ^{13}C NMR (100 MHz, $CDCl_3$) δ 14.1, 22.7, 23.1, 26.9, 29.1, 29.8, 30.6, 30.9, 31.9, 40.5, 65.8.

Hammett and Taft Studies

General Procedure. An oven-dried vial containing a stir bar was placed under a positive pressure of argon, and subjected to three evacuation/backfilling cycles under high vacuum. Samarium(II) iodide (THF solution, typically 0.20 mmol, 2.0 equiv, 0.10 M) was added followed by Et_3N (0.33 mL, 24 equiv) and H_2O (0.043 mL, 24 equiv) with vigorous stirring, which resulted in the formation of a characteristic dark brown color of the SmI_2–Et_3N–H_2O complex. A preformed solution of two substrates (each 0.10 mmol, 1.0 equiv, stock solution in THF, 1.0 mL) was added and the reaction mixture was stirred until decolorization to white had occurred. The reaction mixture was diluted with CH_2Cl_2 (30 mL) and HCl (1 *N*, 30 mL). The aqueous layer was extracted with CH_2Cl_2 (3 x 30 mL), the organic layers were combined, dried over Na_2SO_4, filtered, and concentrated. The sample was analyzed by ^1H NMR ($CDCl_3$, 500 MHz) and GC-MS to obtain conversion and yield using internal standard and comparison with authentic samples.

Table SI-2. Electronic Influence on the Relative Rates in the Reduction of Esters – Hammett Correlation Study.[a]

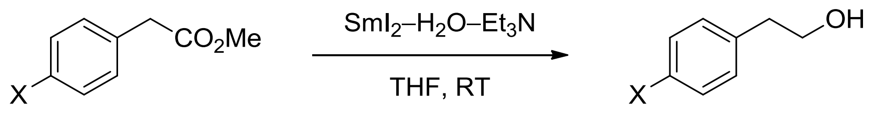

ρ = +0.43

entry	X	k_X/k_H[a]	Hammett constant
1	CF_3	1.79	0.54
2	Cl	1.44	0.23
3	F	1.22	0.06
4	H	1.00	0
5	MeO	0.81	-0.27

[a]Relative reactivity values determined from product distribution by ^1H NMR and/or GC of crude reaction mixtures.

Figure SI-1. Hammett plot in the Reduction of 4-Phenylacetic Methyl Esters with SmI_2–Et_3N–H_2O.[a]

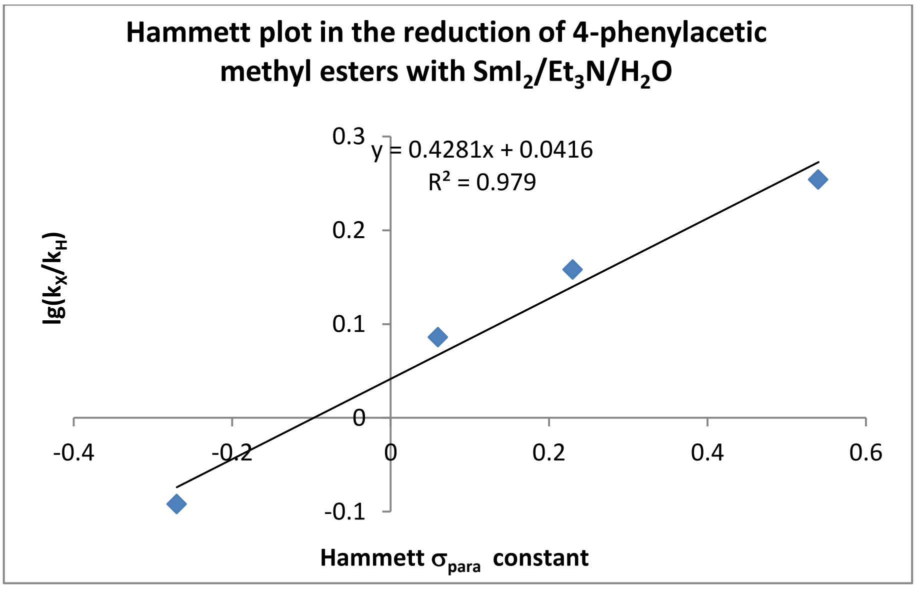

Table SI-3. Steric Influence on the Relative Rates in the Reduction of Esters – Taft E_S Correlation Study.[a]

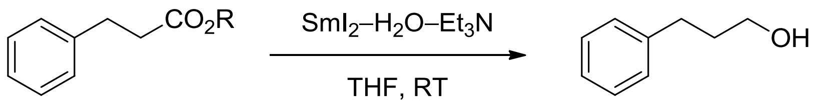

E_S = +0.97

entry	R	k_X/k_{Me}[a]	Taft E_S parameter
1	Me	1.00	0
2	Et	0.55	-0.07
3	*i*-Pr	0.19	-0.47
4	*t*-Bu	0.026	-1.54
5	Ph	6.20	-2.55

[a]Relative reactivity values determined from product distribution by ^{1}H NMR and/or GC of crude reaction mixtures.

Figure SI-2. Taft plot in the Reduction of Hydrocinnamic Acid Esters with SmI_2–Et_3N–H_2O.[a]

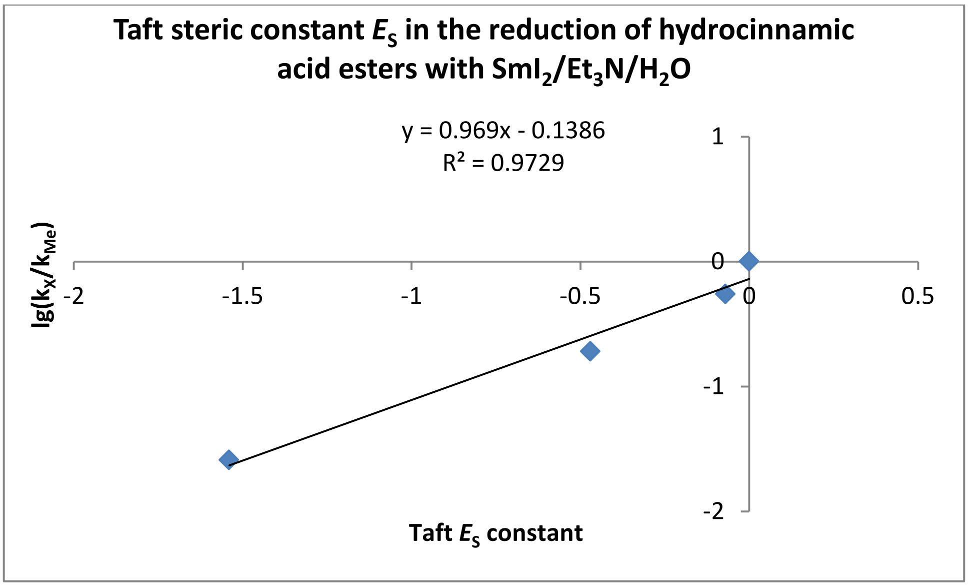

Methyl 2-(4-(trifluoromethyl)phenyl)acetate (Table SI-2, entry 1). ^{1}H NMR (400 MHz, $CDCl_3$) δ 3.70 (s, 2 H), 3.72 (s, 3 H), 7.42 (d, *J* = 8.4 Hz, 2 H), 7.60 (d, *J* = 8.4 Hz, 2 H); ^{13}C NMR (100 MHz, $CDCl_3$) δ 40.8, 52.2, 122.8, 125.5 (q, J^3 = 3.7 Hz), 129.5 (q, J^2 = 32.1 Hz), 129.7, 138.0, 171.2; ^{19}F (376 MHz, $CDCl_3$) δ -62.6.

Methyl 2-(4-chlorophenyl)acetate (Table SI-2, entry 2). ^{1}H NMR (300 MHz, $CDCl_3$) δ 3.61 (s, 2 H), 3.71 (s, 3 H), 7.23 (d, *J* = 8.4 Hz, 2 H), 7.31 (d, *J* = 8.4 Hz, 2 H); ^{13}C NMR (75 MHz, $CDCl_3$) δ 40.4, 52.2, 128.7, 130.7, 132.4, 133.1, 171.6.

Methyl 2-(4-fluorophenyl)acetate (Table SI-2, entry 3). ^{1}H NMR (300 MHz, $CDCl_3$) δ 3.61 (s, 2 H), 3.70 (s, 3 H), 7.02 (t, *J* = 8.7 Hz, 2 H), 7.26 (dd, *J* = 5.4, 8.4 Hz, 2 H); ^{13}C NMR (75 MHz, $CDCl_3$) δ 40.2, 52.1, 115.4 (d, J^2 = 21.7 Hz), 129.7 (d, J^4 = 3.2 Hz), 130.9 (d, J^3 = 7.6 Hz), 162.1 (d, J^1 = 243.9 Hz), 171.9; ^{19}F (376 MHz, $CDCl_3$) δ -115.7.

Methyl 2-(4-methoxyphenyl)acetate (Table SI-2, entry 5). ^{1}H NMR (300 MHz, $CDCl_3$) δ 3.59 (s, 2 H), 3.71 (s, 3 H), 3.81 (s, 3 H), 6.89 (d, *J* = 8.7 Hz, 2 H), 7.22 (d, *J* = 8.7 Hz, 2 H); ^{13}C NMR (75 MHz, $CDCl_3$) δ 40.3, 52.0, 55.3, 114.0, 126.1, 130.3, 158.7, 172.4.

Ethyl 3-phenylpropanoate (Table SI-3, entry 2). ^{1}H NMR (300 MHz, $CDCl_3$) δ 1.27 (t, *J* = 7.2 Hz, 3 H), 2.65 (t, *J* = 7.5 Hz, 2 H), 2.99 (t, *J* = 7.5 Hz, 2 H), 4.16 (q, *J* = 7.2 Hz, 2 H), 7.19-7.26 (m, 3 H), 7.28-7.36 (m, 2 H); ^{13}C NMR (75 MHz, $CDCl_3$) δ 14.2, 31.0, 36.0, 60.4, 126.2, 128.3, 128.5, 140.6, 172.9.

Isopropyl 3-phenylpropanoate (Table SI-3, entry 3). ^{1}H NMR (300 MHz, $CDCl_3$) δ 1.24 (d, *J* = 6.3 Hz, 6 H), 2.63 (t, *J* = 7.8 Hz, 2 H), 2.99 (t, *J* = 7.5 Hz, 2 H), 4.99-5.11 (m, 1 H), 7.19-7.27 (m, 3 H), 7.28-7.36 (m, 2 H); ^{13}C NMR (75 MHz, $CDCl_3$) δ 21.8, 31.1, 36.3, 67.8, 126.2, 128.4, 128.5, 140.6, 172.5.

***tert*-Butyl 3-phenylpropanoate (Table SI-3, entry 4).** ^{1}H NMR (300 MHz, $CDCl_3$) δ 1.46 (s, 9 H), 2.58 (t, *J* = 7.5 Hz, 2 H), 2.95 (t, *J* = 7.5 Hz, 2 H), 7.19-7.26 (m, 3 H), 7.28-7.36 (m, 2 H); ^{13}C NMR (75 MHz, $CDCl_3$) δ 28.1, 31.2, 37.1, 80.4, 126.1, 128.4, 128.4, 140.8, 172.3.

2-(4-(Trifluoromethyl)phenyl)ethanol (Table SI-2, entry 1). ^{1}H NMR (300 MHz, $CDCl_3$) δ 1.51 (br, 1 H), 2.95 (t, J = 6.6 Hz, 2 H), 3.92 (t, J = 6.6 Hz, 2 H), 7.37 (d, J = 8.1 Hz, 2 H), 7.59 (d, J = 8.1 Hz, 2 H); ^{13}C NMR (75 MHz, $CDCl_3$) δ 38.9, 63.2, 122.5, 125.5 (q, J^3 = 3.8 Hz), 129.3 (q, J^2 = 32.5 Hz), 129.4, 142.8; ^{19}F (376 MHz, $CDCl_3$) δ -62.4.

2-(4-Chlorophenyl)ethanol (Table SI-2, entry 2). ^{1}H NMR (300 MHz, $CDCl_3$) δ 1.48 (br, 1 H), 2.86 (t, J = 6.6 Hz, 2 H), 3.87 (t, J = 6.6 Hz, 2 H), 7.19 (d, J = 8.4 Hz, 2 H), 7.31 (d, J = 8.1 Hz, 2 H); ^{13}C NMR (75 MHz, $CDCl_3$) δ 38.5, 63.5, 128.7, 130.4, 132.3, 137.0.

2-(4-Fluorophenyl)ethanol (Table SI-2, entry 3). ^{1}H NMR (300 MHz, $CDCl_3$) δ 1.40 (br, 1 H), 2.77 (t, J = 6.6 Hz, 2 H), 3.77 (t, J = 6.6 Hz, 2 H), 6.93 (t, J = 8.7 Hz, 2 H), 7.12 (dd, J = 5.7, 8.7 Hz, 2 H); ^{13}C NMR (75 MHz, $CDCl_3$) δ 38.3, 63.5, 115.4 (d, J^2 = 21.2 Hz), 130.4 (d, J^3 = 7.7 Hz), 134.2 (d, J^4 = 3.2 Hz), 161.7 (d, J^1 = 242.8 Hz); ^{19}F (376 MHz, $CDCl_3$) δ -116.8.

2-(4-Methoxyphenyl)ethanol (Table SI-2, entry 5). ^{1}H NMR (300 MHz, $CDCl_3$) δ 1.55 (br, 1 H), 2.84 (t, J = 6.6 Hz, 2 H), 3.82 (s, 3 H), 3.85 (t, J = 6.6 Hz, 2 H), 6.88 (d, J = 8.7 Hz, 2 H), 7.17 (d, J = 8.7 Hz, 2 H); ^{13}C NMR (75 MHz, $CDCl_3$) δ 38.3, 55.3, 63.8, 114.1, 130.0, 130.4, 158.3.

Radical Clock Experiments

<u>*General Procedure.*</u> An oven-dried vial containing a stir bar was placed under a positive pressure of argon, and subjected to three evacuation/backfilling cycles under high vacuum. Samarium(II) iodide (THF solution, typically 0.20-0.80 mmol, 2.0-8.0 equiv, 0.10 M) was added followed by Et_3N (0.33 mL, 24 equiv) and H_2O (0.043 mL, 24 equiv) with vigorous stirring, which resulted in the formation of a characteristic dark brown color of the SmI_2–Et_3N–H_2O complex. A solution of ester substrate (0.10 mmol, 1.0 equiv, stock solution in THF, 1.0 mL) was added and the reaction mixture was stirred for the indicated time. The excess of Sm(II) was oxidized by bubbling air through the reaction mixture, and the reaction mixture was diluted with CH_2Cl_2 (30 mL) and HCl (1 *N*, 30 mL). The aqueous layer was extracted with CH_2Cl_2 (3 x 30 mL), the organic layers were combined, dried over Na_2SO_4, filtered, and concentrated. The sample was analyzed by ^{1}H NMR ($CDCl_3$, 500 MHz) and GC-MS to obtain conversion and yield using internal standard and comparison with authentic samples. In all other cases, the Sm(II) reagent was preformed by adding the specified additive to the SmI_2 solution prepared as described above, and stirring until the color characteristic to the particular Sm(II) complex had appeared (SmI_2–H_2O: burgundy red; SmI_2–MeOH: dark brown; SmI_2–HMPA: purple; SmI_2–LiCl: green; SmI_2–Et_3N: dark blue).

Table SI-4. Summary of the Radical Clock Experiments using SmI_2–Et_3N–H_2O and SmI_2–H_2O Complexes in the Reductive Opening of **3**.

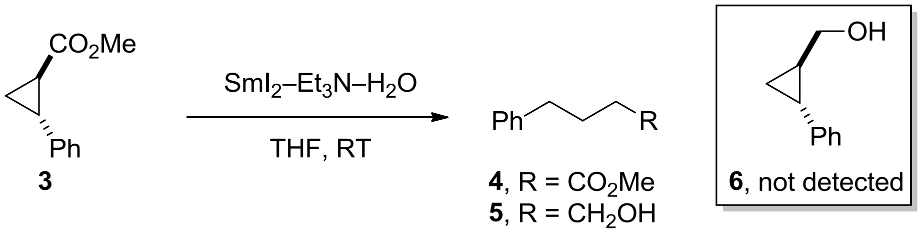

entry	SmI_2 (equiv)	Et_3N (equiv)	H_2O (equiv)	time[a] (h)	conv.[b] (%)	**4**[b] (%)	**5**[b] (%)	yield[c] (%)
1	4	24	24	1 min	80	75	5	80
2	8	48	48	2 h	>98	<2	98	98
3	2	-	415	1 min	1.7	1.7	-	1.7
4	4	-	833	15 min	4.1	4.1	-	4.1
5	4	-	200	5 min	11	11	-	11
6	8	-	200	2 h	53	53	-	53

All reactions carried out using standard Schlenk techniques. [a]Quenched with air after the indicated time. [b]Determined by ^{1}H NMR and/or GC-MS of crude reaction mixtures and comparison with authentic samples. [c]Combined yield of **4** and **5**. Conversion = (100-SM). In all entries **6** was not detected (<2.0%).

Table SI-5. Summary of the Radical Clock Experiments using Sm(II) Complexes with Different Redox Potentials in the Reductive Opening of **3**.

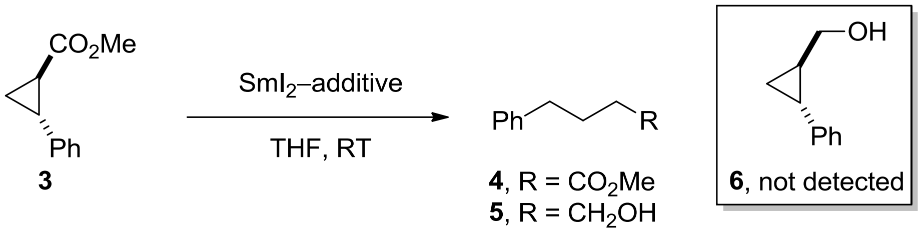

entry	SmI_2 (equiv)	additive	(equiv)	time[a] (h)	conv.[b] (%)	**4**[b] (%)	**5**[b] (%)	yield[c] (%)
1	4	Et_3N	48	2 h	<5[d]	-	-	<5
2	4	HMPA	24	2 h	decomp.	-	-	<5
3	4	LiCl	48	2 h	<5	-	-	<5
4	4	MeOH	370	2 h	<5	-	-	<5
5	4	-	-	72 h	<5	-	-	<5

All reactions carried out using standard Schlenk techniques. [a]Quenched with air after the indicated time. [b]Determined by ^1H NMR and/or GC-MS of crude reaction mixtures and comparison with authentic samples. [c]Combined yield of **4** and **5**. [d]66:34 ratio of ester to acid. In all entries, >85% recovery of the SM. In all entries, **6** was not detected (<2.0%).

Table SI-6. Summary of the Radical Clock Experiments using Sm(II) Complexes in the Reductive Opening of **3-SI**.

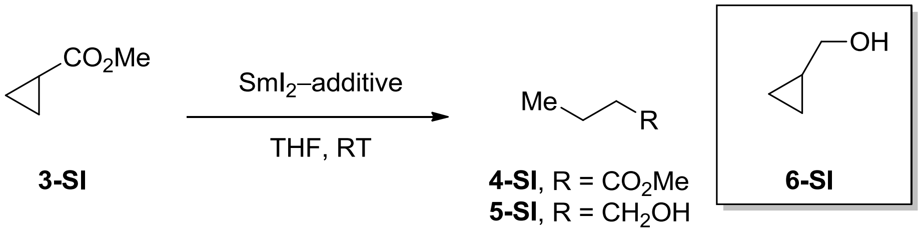

entry	SmI_2 (equiv)	additive	(equiv)	time[a] (h)	conv.[b] (%)	**4-SI**[b] (%)	**5-SI**[b] (%)	**6-SI**[b] (%)
1	2	H_2O/morpholine	24/24	2 h	62.8	34.2	26.6	2.0
2	8	H_2O/morpholine	48/48	2 h	>98	<2	93.6	6.4
3	8	MeOH	980	2 h	<2	-	-	<2
4	8	H_2O	200	2 h	<2	-	-	<2
5	8	H_2O	800	2 h	<2	-	-	<2

All reactions carried out using standard Schlenk techniques. [a]Quenched with air after the indicated time. [b]Determined by GC-MS analysis of crude reaction mixtures and comparison with authentic samples. Morpholine was used a Brønsted base to facilitate analysis of the crude reaction mixtures. Similar results were obtained with Et_3N. Agilent 7890A GC System and Agilent 5975C inert XL EI/CI MSD, Agilent HP-5MS column (19091S-433) (length 30 m, internal diameter 0.25 mm, film 0.25 μm), helium as the carrier gas at a flow rate of 1 mL/min, initial oven temperature of 40 °C. 15 °C/min ramp, after 40 °C hold for 3 min to 300 °C, then hold at 300 °C for 5 min: retention time: **3-SI**, 3.93 min; **4-SI**, 3.55 min; **5-SI**, 3.15 min; **6-SI**, 3.30 min.

Table SI-7. Summary of the Radical Clock Experiments using Sm(II) Complexes in the Reductive Cyclization of **7-SI**.

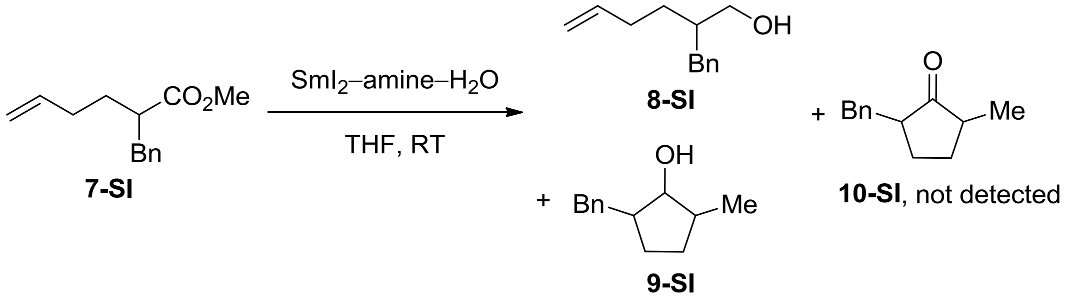

entry	SmI_2 (equiv)	additive	(equiv)	time[a] (h)	conv.[b] (%)	**8-SI**[b] (%)	**9-SI**[b] (%)	yield[c] (%)
1	6	H_2O	200	2 h	<2	-	-	<2
2	6	Et_3N/H_2O	48/48	18 h	91	60	40	90
3	6	pyrrolidine/ H_2O	48/48	18 h	>98	46	54	95

All reactions carried out using standard Schlenk techniques. [a]Quenched with air after the indicated time. [b]Determined by ^{1}H NMR and/or GC-MS of crude reaction mixtures and comparison with authentic samples. [c]Combined yield of **8-SI** and **9-SI**. In all entries, **10-SI** was not detected (<2.0%). Entry 2, **9-SI**, dr = 41:33:15:11; entry 3, **9-SI**, dr = 39:32:18:11. **10-SI** was not detected in the reaction of **7-SI** with limiting Sm(II) (SmI_2–Et_3N–H_2O, 2-24-24 equiv). Standard procedure for reductions using SmI_2–amine–H_2O was followed.

Rac-(1*R*,2*R*)-Methyl 2-phenylcyclopropanecarboxylate (3, Tables SI-4, SI-5). ^{1}H NMR (500 MHz, $CDCl_3$) δ 1.22-1.27 (m, 1 H), 1.51-1.55 (m, 1 H), 1.81-1.85 (m, 1 H), 2.43-2.48 (m, 1 H), 3.64 (s, 3 H), 7.02 (d, *J* = 7.5 Hz, 2 H), 7.12 (t, *J* = 7.5 Hz, 1 H), 7.20 (t, *J* = 7.5 Hz, 2 H); ^{13}C NMR (125 MHz, $CDCl_3$) δ 17.0, 24.0, 26.3, 51.9, 126.2, 126.5, 128.5, 140.0, 173.9.

Methyl 4-phenylbutanoate (4, Tables SI-4, SI-5). ^{1}H NMR (300 MHz, $CDCl_3$) δ 1.96-2.07 (m, 2 H), 2.38 (t, *J* = 7.5 Hz, 2 H), 2.70 (t, *J* = 7.2 Hz, 2 H), 3.71 (s, 3 H), 7.19-7.27 (m, 3 H), 7.30-7.37 (m, 2 H); ^{13}C NMR (75 MHz, $CDCl_3$) δ 26.6, 33.4, 35.2, 51.5, 126.0, 128.4, 128.5, 141.4, 173.9.

4-Phenylbutan-1-ol (5, Tables SI-4, SI-5). ^{1}H NMR (300 MHz, $CDCl_3$) δ 1.44-1.67 (m, 4 H), 1.71 (br, 1 H), 2.56 (t, *J* = 7.8 Hz, 2 H), 3.55 (t, *J* = 6.6 Hz, 2 H), 7.06-7.13 (m, 3 H), 7.16-7.23 (m, 2 H); ^{13}C NMR (75 MHz, $CDCl_3$) δ 27.6, 32.3, 35.7, 62.8, 125.8, 128.3, 128.5, 142.4.

Rac-((1*R*,2*R*)-2-Phenylcyclopropyl)methanol (6, Tables SI-4, SI-5). ^{1}H NMR (500 MHz, $CDCl_3$) δ 0.82-0.91 (m, 2 H), 1.34-1.41 (m, 1 H), 1.65 (br, 1 H), 1.72-1.76 (m, 1 H), 3.49-3.57 (m, 2 H), 6.99 (dd, *J* = 1.5, 7.5 Hz, 2 H), 7.07 (tt, *J* = 1.5, 7.0 Hz, 1 H), 7.18 (t, *J* = 7.5 Hz, 2 H); ^{13}C NMR (125 MHz, $CDCl_3$) δ 13.9, 21.3, 25.3, 66.6, 125.7, 125.9, 128.4, 142.5.

Rac-(1*R*,2*R*)-2-Phenylcyclopropanecarboxylic acid (Tables SI-4, SI-5). ^{1}H NMR (300 MHz, $CDCl_3$) δ 1.39-1.48 (m, 1 H), 1.66-1.73 (m, 1 H), 1.90-1.97 (m, 1 H), 2.59-2.67 (m, 1 H), 7.09-7.16 (m, 2 H), 7.21-7.35 (m, 3 H); ^{13}C NMR (100 MHz, $CDCl_3$) δ 17.6, 24.1, 27.2, 126.3, 126.8, 128.6, 139.5, 180.2.

Methyl 2-benzylhex-5-enoate (7-SI, Table SI-7). ^{1}H NMR (300 MHz, $CDCl_3$) δ 1.56-1.68 (m, 1 H), 1.72-1.86 (m, 1 H), 1.98-2.19 (m, 2 H), 2.67-2.82 (m, 2 H), 2.89-3.03 (m, 1 H), 3.62 (s, 3 H), 4.96-5.07 (m, 2 H), 5.71-5.84 (m, 1 H), 7.14-7.33 (m, 5 H); ^{13}C NMR (75 MHz, $CDCl_3$) δ 31.2, 31.5, 38.5, 47.0, 51.4, 115.2, 126.4, 128.4, 128.9, 137.7, 139.3, 175.9.

2-Benzylhex-5-en-1-ol (8-SI, Table SI-7). ^{1}H NMR (300 MHz, $CDCl_3$) δ 1.25-1.48 (m, 2 H), 1.62 (br, 1 H), 1.67-1.80 (m, 1 H), 1.94-2.12 (m, 2 H), 2.56 (d, *J* = 7.2 Hz, 2 H), 3.36-3.48 (m, 2 H), 4.83-4.97 (m, 2 H), 5.62-5.77 (m, 1 H), 7.07-7.14 (m, 3 H), 7.16-7.23 (m, 2 H); ^{13}C NMR (75 MHz, $CDCl_3$) δ 29.9, 31.2, 37.6, 41.9, 64.6, 114.7, 126.0, 128.4, 129.2, 138.8, 140.7.

2-Benzyl-5-methylcyclopentanol (9-SI, Table SI-7). ^{1}H NMR (400 MHz, $CDCl_3$) δ (ca. 65:35 mixture of diastereoisomers) 0.90 (d, *J* = 6.8 Hz, 3 H, minor), 0.92 (d, *J* = 6.8 Hz, 3 H, major), 0.98-1.31 (m, 3 H), 1.58-2.03 (m, 3 H), 2.04-2.17 (m, 1 H), 2.48 (dd, *J* = 8.4, 13.6 Hz, 1 H, major), 2.55 (dd, *J* = 8.0, 13.6 Hz, 1 H, minor), 2.70 (dd, *J* = 7.2, 13.6 Hz, 1 H, major), 2.81 (dd, *J* = 7.2, 13.6 Hz, 1 H, minor), 3.61 (dd, *J* = 2.8, 5.2 Hz, 1 H, minor), 3.71 (dd, *J* = 4.8, 5.6 Hz, 1 H, major), 7.09-7.24 (m, 5 H); ^{13}C NMR (100 MHz, $CDCl_3$) δ (ca. 65:35 mixture of diastereoisomers) 13.9, 19.8, 29.0, 29.1, 30.6, 31.0, 35.4, 37.5, 40.6, 42.4, 45.1, 48.6, 80.0, 80.9, 125.7, 125.9, 128.4, 128.4, 128.8, 128.9, 141.2, 141.9.

Scheme SI-1. Reductive Opening of Cyclopropyl Carbinol **6** using SmI_2–Et_3N–H_2O – Control Experiment to C–C Bond Cleavage.

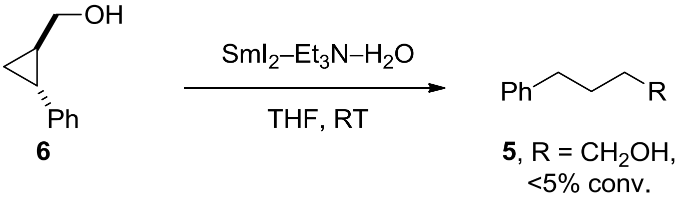

According to the general procedure, an oven-dried vial containing a stir bar was charged with samarium(II) iodide (THF solution, 0.40 mmol, 8.0 equiv, 0.085 M), Et_3N (0.50 mL, 72 equiv) and H_2O (0.065 mL, 72 equiv) with vigorous stirring, which resulted in the formation of a characteristic dark brown color of the SmI_2–Et_3N–H_2O complex. A solution of ((1*R*,2*R*)-2-phenylcyclopropyl)methanol (0.05 mmol, 1.0 equiv, stock solution in THF, 1.0 mL) was added and the reaction mixture was stirred for 18 h at room temperature. After the standard work-up as described above, the sample was analyzed by ^{1}H NMR to obtain conversion and yield using internal standard: conversion <5%; yield of recovered starting material: 96%, indicating that the reductive opening of phenyl-activated cyclopropyl carbinols with SmI_2–Et_3N–H_2O is not operative under these reaction conditions. We thank Prof. Chaozhong Li (Shanghai Institute of Organic Chemistry, Chinese Academy of Sciences) for suggesting this experiment.

Additional Discussion. To determine the rate of the reduction of acyl-type radicals with Sm(II), three types of radical probes with gradually increasing sensitivity[15] were selected and subjected to the standard reaction conditions with SmI_2–amine–H_2O:

a) trans-cyclopropyl-phenyl containing radical clock **3** (Tables 4-SI and 5-SI) (k_{frag} = ca. 3 x 10^{11} s^{-1} at 25 °C).[15]

b) cyclopropyl-containing radical clock **3-SI** (Table 6-SI) (k_{frag} = ca. 9.4 x 10^7 s^{-1} at 25 °C).[15]

c) substituted 5-exo-hexenyl radical clock **7-SI** (Table 7-SI) ($k_{5\text{-}exo}$ = ca. 2.3 x 10^5 s^{-1} at 25 °C).[15] The relative product distribution obtained in these experiments allows to approximate a bimolecular rate constant for the reduction of acyl-type radicals using Sm(II).[15-18] The rate constant k_{SmI2} was estimated as follows: k_{SmI2} = (red/cycl) x $k_{5\text{-}exo}$ x $[SmI_2]^{-1}$.[15-18] In all cases the concentration of SmI_2 was corrected for changing the reaction volumes as indicated in Tables 4-SI to 7-SI. The product distribution was quantified by ^{1}H NMR and GC-MS analysis of the crude reaction mixtures and comparison with authentic samples. The approximated k_{SmI2} rate constant as estimated from the results obtained in Table 6-SI using

cyclopropyl-containing radical clock **3-SI** indicates that the reduction of acyl-type radicals under these reaction conditions is comparable to a unimolecular reaction with k of about 10^8 s^{-1}. The approximated k_{SmI2} rate constant as estimated from the results obtained in Table 7-SI using the substituted analogue of 5-exo-cyclohexenyl radical clock **7-SI** indicates that the reduction of acyl-type radicals is comparable to a unimolecular reaction with k of about 10^7 s^{-1}. Overall, these results demonstrate that the reduction of acyl-type radicals with SmI_2–amine–H_2O is remarkably fast (previously, the rate constant of 7 x 10^6 $M^{-1}s^{-1}$ was determined for the reduction of primary alkyl iodides with SmI_2–HMPA complexes, which is a benchmark reaction in this field).[16-18] Equally importantly, these results indicate that acyl-type radicals generated using SmI_2–amine–H_2O can participate in a wide range of cross-coupling reactions with unactivated radical acceptors.[19] Studies in this direction are a subject of current research in our laboratory and these results will be reported shortly.

Epimerization Studies

Scheme SI-2. Epimerization Study of Enantioenriched Ester **7** under SmI_2–Et_3N–H_2O Reaction Conditions.

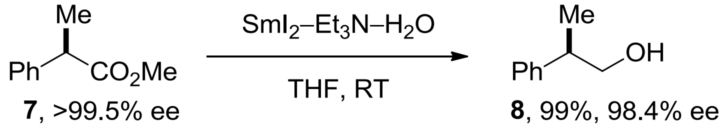

According to the general procedure using the preformed Sm(II) complex, (*R*)-methyl 2-phenylpropanoate (0.10 mmol, >99.5% ee), was reacted with samarium(II) iodide (0.8 mmol), water (4.8 mmol) and triethylamine (4.8 mmol) for 2 h at rt to afford the title compound in 99% yield. ^{1}H NMR (500 MHz, $CDCl_3$) δ 1.21 (d, *J* = 7.0 Hz, 3H), 1.33 (br, 1H), 2.84-2.91 (m, 1H), 3.63 (d, *J* = 7.0 Hz, 2H), 7.14-7.18 (m, 3H), 7.24-7.28 (m, 2H); ^{13}C NMR (125 MHz, $CDCl_3$) δ 17.6, 42.5, 68.7, 126.7, 127.5, 128.7, 143.7. HPLC analysis (chiralpak IA 28C, hexanes/*i*-PrOH 99/1, 1.0 mL/min, 220 nm) indicated 98.4% ee: t_R (minor) = 18.42 minutes, t_R (major) = 19.48 minutes. In addition, the reaction of (*R*)-**7** (0.1 mmol, >99.5% ee) was stopped at half-conversion (conditions: SmI_2 (0.6 mmol), water (3.6 mmol) and triethylamine (3.6 mmol), preformed Sm(II) complex, 5 min, room temperature) to give the title product in 44% yield (44% conv.). HPLC analysis chiralpak IA 28C, hexanes/*i*-PrOH 99/1, 1.0 mL/min, 220 nm) indicated 98.9% ee: t_R (minor) = 17.75 minutes, t_R (major) = 19.02 minutes. Taken together, these results indicate that enolization does not occur to an appreciable extent under these reaction conditions, which is consistent with fast first electron transfer.

$H_2{}^{18}O$ Incorporation Studies

General Procedure. According to the general procedure using the preformed Sm(II) complex, methyl 3-phenylpropanoate (0.10 mmol), was reacted with samarium(II) iodide (0.6 mmol), $H_2{}^{18}O$ (4.8 mmol) and triethylamine (4.8 mmol) for 18 h at rt to afford the title compound in 99% yield, <2.0% of ^{18}O incorporation (determined by HRMS analysis) (Table SI-8, entry 1). In addition, the reaction of a hindered isopropyl 3-phenylpropanoate (Table SI-8, entry 2) and electronically-activated phenyl 3-phenylpropanoate (Table SI-8, entry 3) under the reaction conditions described above afforded the title compound in 92% and 98% yield, respectively, with <2.0% of ^{18}O incorporation (determined by HRMS analysis). These results are consistent with direct electron transfer to the ester carbonyl groups even in the case of sterically-hindered (Table SI-8, entry 2) and electronically-activated substrates (Table SI-8, entry 3). See also Scheme SI-3 (Page SI-21) for experiment using the SmI_2–NaOH–$H_2{}^{18}O$ system.

Table SI-8. Determination of $H_2{}^{18}O$ Incorporation in the Reduction of Esters of Hydrocinnamic Acid using SmI_2–Et_3N–$H_2{}^{18}O$.

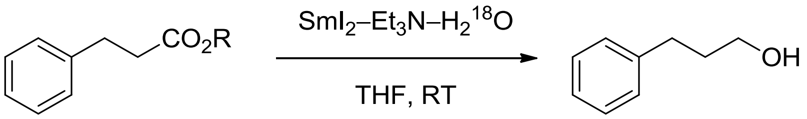

entry	R	SmI_2 (equiv)	Et_3N (equiv)	H_2O (equiv)	time[a] (h)	conv.[b] (%)	yield[b] (%)	^{18}O[c] (%)
1	Me	6	48	48	18 h	>98	99	<2
2	*i*-Pr	6	48	48	18 h	>98	92	<2
3	Ph	6	48	48	18 h	>98	98	<2

All reactions carried out using standard Schlenk techniques. [a]Quenched with air after the indicated time. [b]Determined by ^1H NMR. [c]Determined by HRMS.

Reagent Stoichiometry Studies

General Procedure. An oven-dried vial containing a stir bar was placed under a positive pressure of argon, and subjected to three evacuation/backfilling cycles under high vacuum. Samarium(II) iodide (THF solution, 0.45 mmol, 4.5 equiv, 0.10 M) was added followed by Et_3N (4-24 equiv) and H_2O (4-24 equiv) with vigorous stirring, which resulted in the formation of a characteristic dark brown color of the SmI_2–Et_3N–H_2O complex. A solution of ester substrate (0.10 mmol, 1.0 equiv, stock solution in THF, 1.0 mL) was added and the reaction mixture was stirred for the indicated time. The excess of Sm(II) was oxidized by bubbling air through the reaction mixture, a small aliquot (typically, 1.0 mL) was removed from the reaction mixture, diluted with diethyl ether (2 mL) and HCl (0.1 N, 0.25 mL) and analyzed by GC-MS to obtain product distribution. All data represent values corrected for response factors obtained by analyzing known quantities of the starting materials/products. The required amounts are: H_2O (3 equiv); Et_3N (2 equiv); SmI_2 (1 equiv).

Table SI-9. Determination of the Reagent Stoichiometry of Required for the Reduction of Esters of using SmI_2–Et_3N–H_2O.[a]

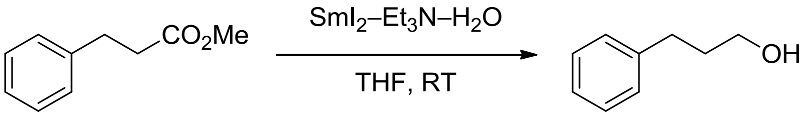

entry	H_2O (equiv)	Et_3N (equiv)	conv. (%)[b]
1	4	4	21.1
2	8	4	17.1
3	4	8	10.0
4	8	8	52.3
5	12	8	82.7
6	8	12	52.8
7	12	12	86.4
8	24	24	>98
9	4	12	7.4
10	12	4	17.1
11	-	12	<2
12	12	-	<2

[a]All reactions carried out using standard Schlenk techniques. Conditions: SmI_2 (4.5 equiv), rt, 24 h. Quenched with air after the indicated time. [b]Determined by GC-MS. Agilent HP-5MS (19091S-433) (length 30 m, internal diameter 0.25 mm, film 0.25 μm), He as the carrier gas, flow rate 1 mL/min, initial oven temp. 50 °C, 25 °C/min ramp, after 50 °C hold for 3 min to 300 °C, then hold at 300 °C for 5 min: retention time: **1**: 9.11 min; **2**: 8.87.

Reduction of Esters using SmI_2–NaOH

<u>*General Procedure.*</u> An oven-dried vial containing a stir bar was charged with sodium hydroxide (pellets, ground in a mortar immediately prior to use), placed under a positive pressure of argon, and subjected to three evacuation/backfilling cycles under high vacuum. Samarium(II) iodide (THF solution, typically 0.60 mmol, 6.0 equiv, 0.080 M) was added followed by ester (0.10 mmol, 1.0 equiv, stock solution in THF, 1.0 mL) and H_2O (typically, 24-48 equiv) with vigorous stirring, which resulted in the formation of a characteristic dark green color of the SmI_2–NaOH–H_2O complex. The excess of Sm(II) was oxidized by bubbling air through the reaction mixture, and the reaction mixture was diluted with CH_2Cl_2 (30 mL) and HCl (1 *N*, 30 mL). The aqueous layer was extracted with CH_2Cl_2 (3 x 30 mL), the organic layers were combined, dried over Na_2SO_4, filtered, and concentrated. The sample was analyzed by ^1H NMR ($CDCl_3$, 500 MHz) and GC-MS to obtain conversion and yield using internal standard and comparison with authentic samples.

Table SI-10. Reduction of Unactivated Esters using the SmI_2–NaOH–H_2O Complex (Modified Kamochi and Kudo Conditions[20-22]).[a]

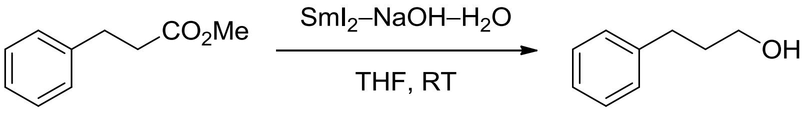

entry	SmI_2 (equiv)	NaOH (equiv)	H_2O (equiv)	time[b]	conv.[c] (%)	acid[c] (%)	yield[c] (%)
1	6	12	167[d]	2 h	40	<5	35
2	6	24	333[d]	60 s[e]	31	51	31
3	6	72	1000[d]	2 min[e,f]	8	34	7
4	6	12	24	2 h	95	<5	92
5	6	12	48	2 h	87	<5	81
6	6	12	12	2 h	84	<5[g]	81
7	8	12	24	2 h	84	<5	79
8	6	12	24	30 s	5.0	<5	5
9	6	12	0	2 h	44[h]	25	33
10	6	12	48	18 h	>95	<5	90

[a]Conditions: entries 1-3: SmI_2 was added to ester, followed by $NaOH_{(aq)}$; entries 4-10: SmI_2 was added to $NaOH_{(solid)}$, followed by ester and H_2O. [b]Quenched with air after the indicated time. [c]Determined by ^1H NMR; conversion is based on reduced starting material and does not include the hydrolysis product. [d]4 *N* $NaOH_{(aq)}$ was used. [e]Oxidation of the Sm(II) reagent was observed after the indicated time. [f]Heterogenous mixture formed immediately upon addition of NaOH. [g]Acid formed in 4.5%. In other entries, when <5, hydrolysis to acid was not observed. [h]Homo-coupling product formed in 23% yield.

To determine whether ester hydrolysis contributes to the mechanism of ester reduction using SmI_2–NaOH–H_2O under these reaction conditions, the reduction was carried out in the presence of $H_2{}^{18}O$ (Scheme SI-3, see also Table SI-8, page SI-18).

Scheme SI-3. Determination of $H_2{}^{18}O$ Incorporation in the Reduction of Esters of Hydrocinnamic Acid using SmI_2–NaOH–$H_2{}^{18}O$.

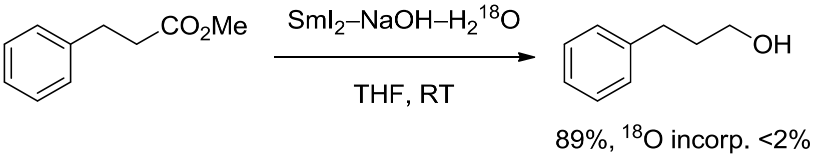

<u>*General Procedure.*</u> According to the general procedure using for the reduction of esters using SmI_2–NaOH–H_2O, methyl 3-phenylpropanoate (0.10 mmol), was reacted with samarium(II) iodide (0.6 mmol), sodium hydroxide (1.2 mmol) and $H_2{}^{18}O$ (4.8 mmol) for 18 h at rt to afford the title compound in 89% yield, >95% conversion, <2.0% of ^{18}O incorporation (determined by HRMS analysis). This result demonstrates that the reduction of esters under these reaction conditions occurs via direct electron transfer to the ester carbonyl groups, which is consistent with the proposed role of amine/H_2O in the general reduction mechanism as described in the manuscript.

Kinetic Experiments

<u>*General Procedure.*</u> An oven-dried vial containing a stir bar was placed under a positive pressure of argon, and subjected to three evacuation/backfilling cycles under high vacuum. Samarium(II) iodide (THF solution, 0.10 M) was added followed by Et_3N and H_2O with vigorous stirring, which resulted in the formation of a characteristic dark brown color of the SmI_2–Et_3N–H_2O complex. A solution of ester substrate (stock solution in THF) was added and the reaction mixture was vigorously stirred under argon. Small aliquots (typically, 0.25 mL) were removed from the reaction mixture at set time intervals, immediately quenched by bubbling air through the reaction mixture, diluted with diethyl ether (2.0 mL) and HCl (0.1 N, 0.25 mL), and analyzed by GC and/or GC-MS to obtain yield and product distribution using internal standard and comparison with authentic samples. Agilent HP-5MS (19091S-433) (length 30 m, internal diameter 0.25 mm, film 0.25 μm), He as the carrier gas, flow rate 1 mL/min, initial oven temp. 90 °C, 10 °C/min ramp, after 90 °C hold for 3 min to 220 °C, then hold at 220 °C for 5 min: Product: 11.50 min, starting material: 12.55 min, standard: 12.45 min.

Ester Order. Conditions: SmI_2 (6.0 equiv), Et_3N (12.0 equiv), H_2O (18.0 equiv), Ester (0.05-0.15 equiv).

Figure SI-3. Determination of Ester Rate Order in the Reduction of **1** using SmI_2–Amine–H_2O.

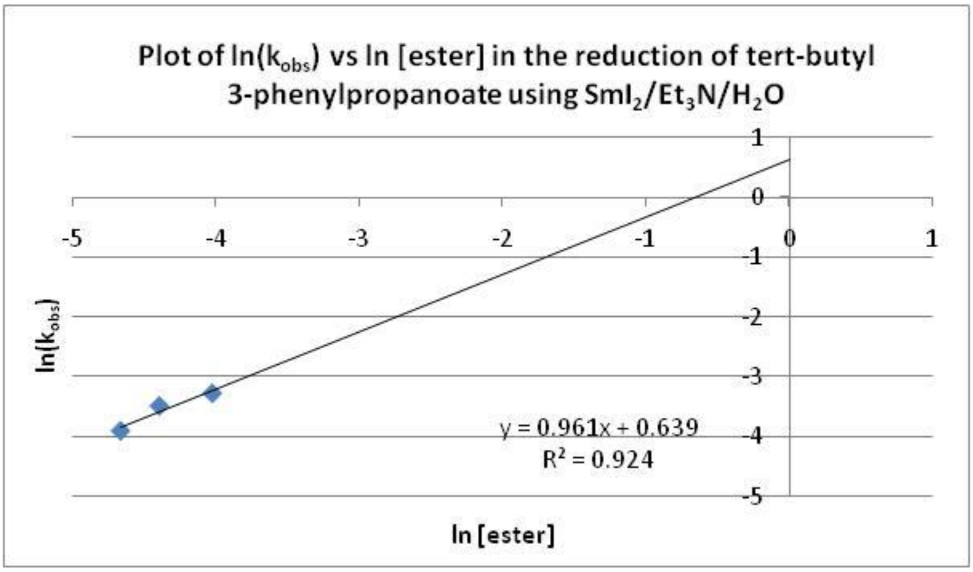

Samarium(II) Iodide Order. Conditions: SmI_2 (5.0-8.0 equiv), Et_3N (12.0 equiv), H_2O (18.0 equiv), Ester (1.0 equiv).

Figure SI-4. Determination of SmI_2 Rate Order in the Reduction of **1** using SmI_2–Amine–H_2O.

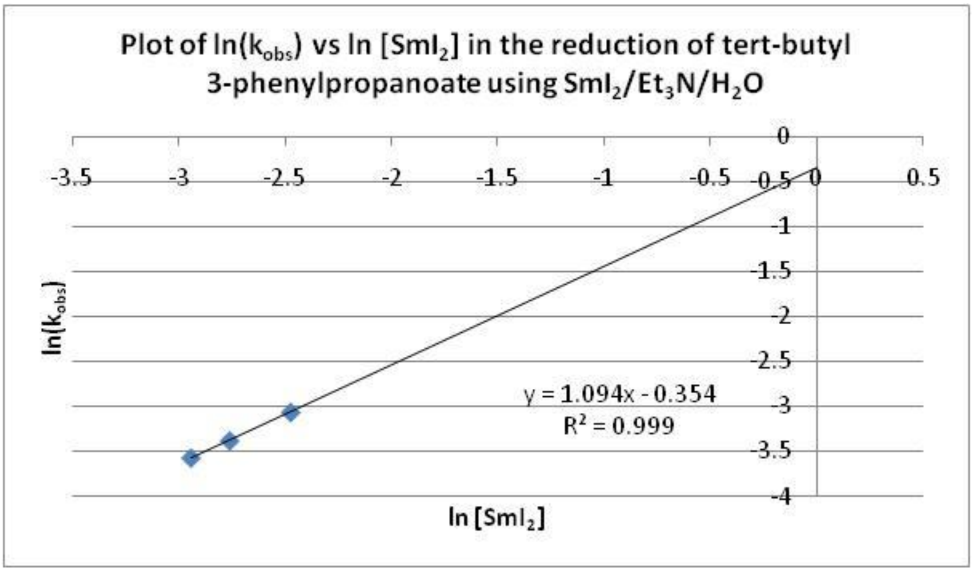

Amine Rate Order. Conditions: SmI_2 (6.0 equiv), amine (6.0-96.0 equiv), H_2O (18.0 equiv), Ester (1.0 equiv).

Figure SI-5. Determination of Amine Rate Order in the Reduction of **1** using SmI_2–Amine–H_2O.

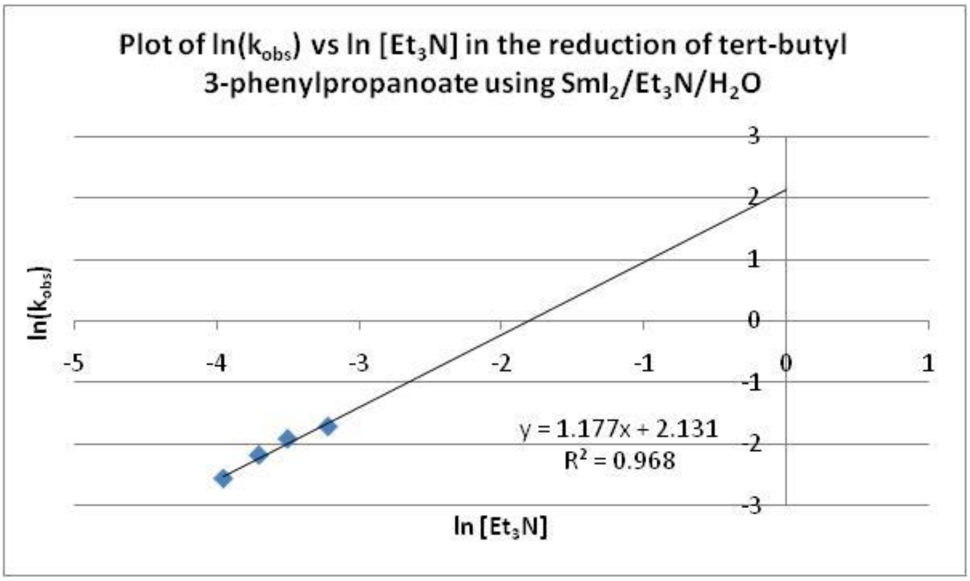

Water Rate Order. Conditions: SmI_2 (6.0 equiv), amine (12.0 equiv), H_2O (6.0-96.0 equiv), Ester (1.0 equiv).

Figure SI-6. Determination of H_2O Rate Order in the Reduction of **1** using SmI_2–Amine–H_2O.

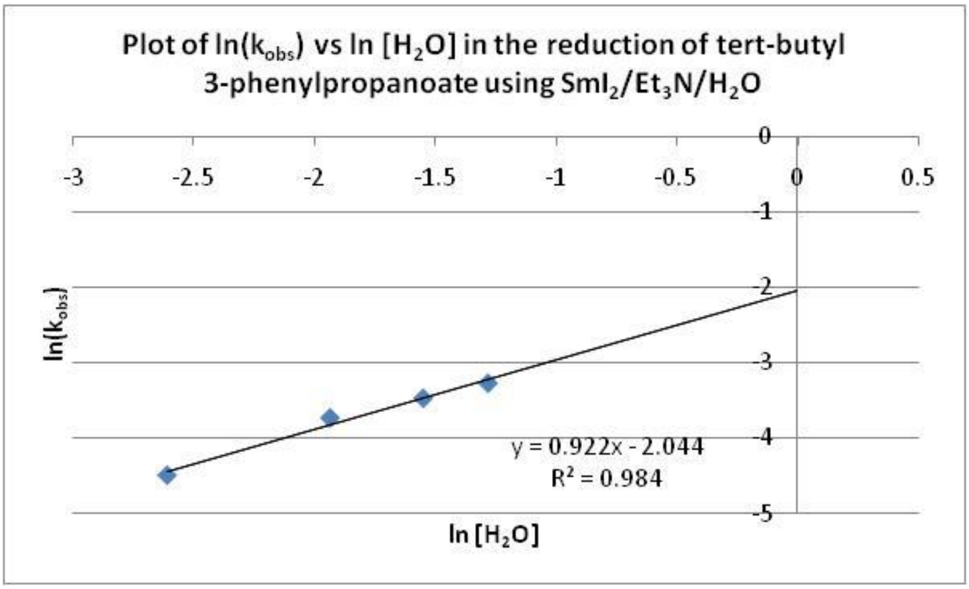

Figure SI-7. Determination of H_2O Rate Order in the Reduction of **1** using SmI_2–Amine–H_2O.

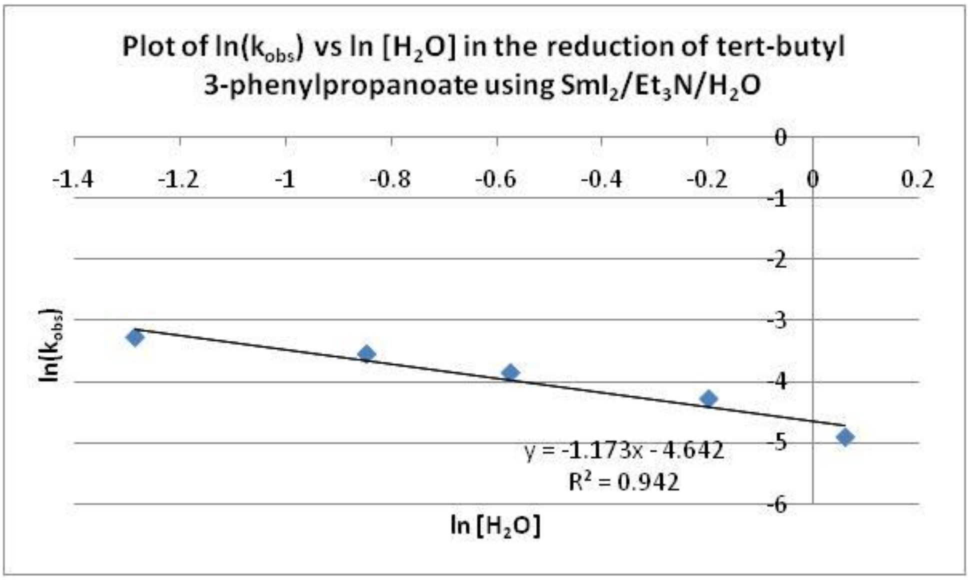

pK_{BH+} of Amines. Conditions: SmI_2 (6.0 equiv), amine (12.0 equiv), H_2O (18.0 equiv), Ester (1.0 equiv).

Figure SI-8. Plot of Initial Rates vs. pK_{BH+} in the Reduction of **1** and 1-Chlorodecane[23] using SmI_2–Amine–H_2O.

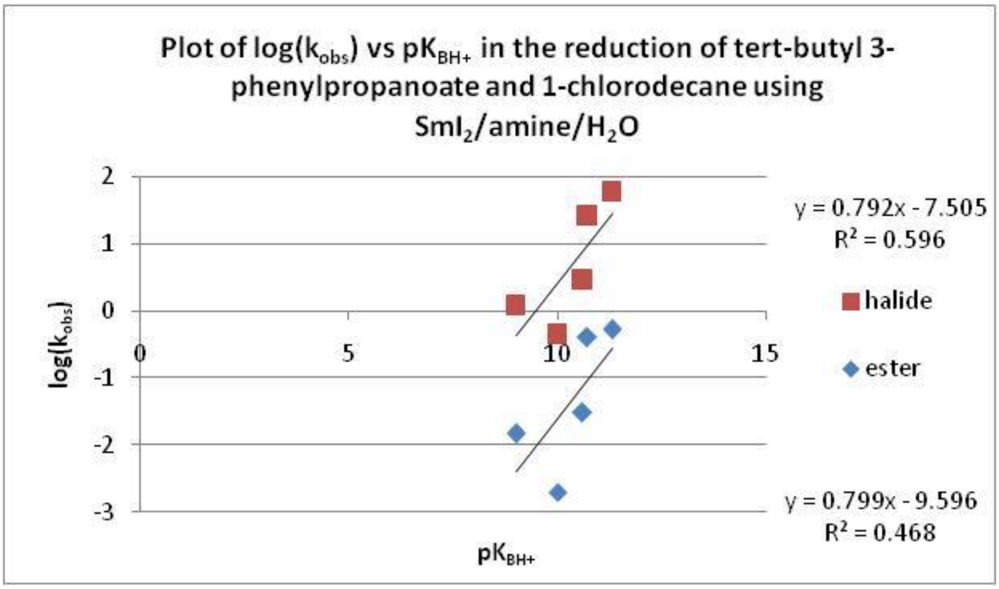

References

1. B. S. Bodnar and P. F. Vogt, *J. Org. Chem.*, **2009**, *74*, 2598.
2. C. A. Mosley, S. J. Myers, E. E. Murray, R. Santangelo, Y. A. Tahirovic, N. Kurtkaya, P. Mullasseril, H. Yuan, P. Lyuboslavsky, P. Le, L. J. Wilson, M. Yepes, R. Dingledine, S. F. Traynelis and D. C. Liotta, *Bioorg. Med. Chem.*, **2009**, *17*, 6463.
3. B. D. Hosangadi and R. H. Dave, *Tetrahedron Lett.*, **1996**, *37*, 6375.
4. L. J. Goossen and A. Döhring, *Synlett*, **2004**, 263.
5. V. V. Rekha, M. V. Ramani, A. Ratnamala, V. Rupakalpana, G. V. Subbaraju, C. Satyanarayana, and C. S. Rao, *Org. Process Res. Dev.*, **2009**, *13*, 769.
6. J. Yamazaki, T. Watanabe and K. Tanaka, *Tetrahedron Asymmetry*, **2001**, *12*, 669.
7. P. C. Bulman Page, M. J. McKenzie, S. M. Allin and D. R. Buckle, *Tetrahedron*, **2000**, *56*, 9683.
8. S. Khatib, O. Nerya, R. Musa, S. Tamir, T. Peter and J. Vaya, *J. Med. Chem.*, **2007**, *50*, 2676.
9. S. W. Wright, D. L. Hageman, A. S. Wright and L. D. McClure, *Tetrahedron Lett.*, **1997**, *38*, 7345.
10. P. Girard, J. L. Namy and H. B. Kagan, *J. Am. Chem. Soc.*, **1980**, *102*, 2693.
11. T. Imamoto and M. Ono, *Chem. Lett.*, **1987**, 501.
12. A. Dáhlen and G. Hilmersson, *Eur. J. Inorg. Chem.*, **2004**, 3020.
13. J. A. Teprovich, Jr., P. K. S. Antharjanam, E. Prasad, E. N. Pesciotta and R. A. Flowers, II, *Eur. J. Inorg. Chem.*, **2008**, 5015.
14. M. Szostak, M. Spain and D. J. Procter, *J. Org. Chem.*, **2012**, *77*, 3049.
15. M. Newcomb, *Tetrahedron*, **1993**, *49*, 1151-1176.
16. E. Hasegawa and D. P. Curran, *Tetrahedron Lett.*, **1993**, *34*, 1717-1720.
17. D. P. Curran, T. L. Fevig, C. P. Jasperse and M. J. Totleben, *Synlett*, **1992**, 943-961.
18. G. A. Molander and C. Kenny, *J. Am. Chem. Soc.*, **1989**, *111*, 8236-8246.
19. C. Chatgilialoglu and A. Studer, *Encyclopedia of Radicals in Chemistry, Biology and Materials*; Wiley-Blackwell: 2012.
20. Y. Kamochi and T. Kudo, *Chem. Lett.*, **1991**, 893.
21. Y. Kamochi and T. Kudo, *Tetrahedron Lett.*, **1991**, *32*, 3511.
22. Y. Kamochi and T. Kudo, *Bull. Chem. Soc. Jpn.*, **1992**, *65*, 3049.
23. A. Dáhlen and G. Hilmersson, *J. Am. Chem. Soc.*, **2005**, *127*, 8340.

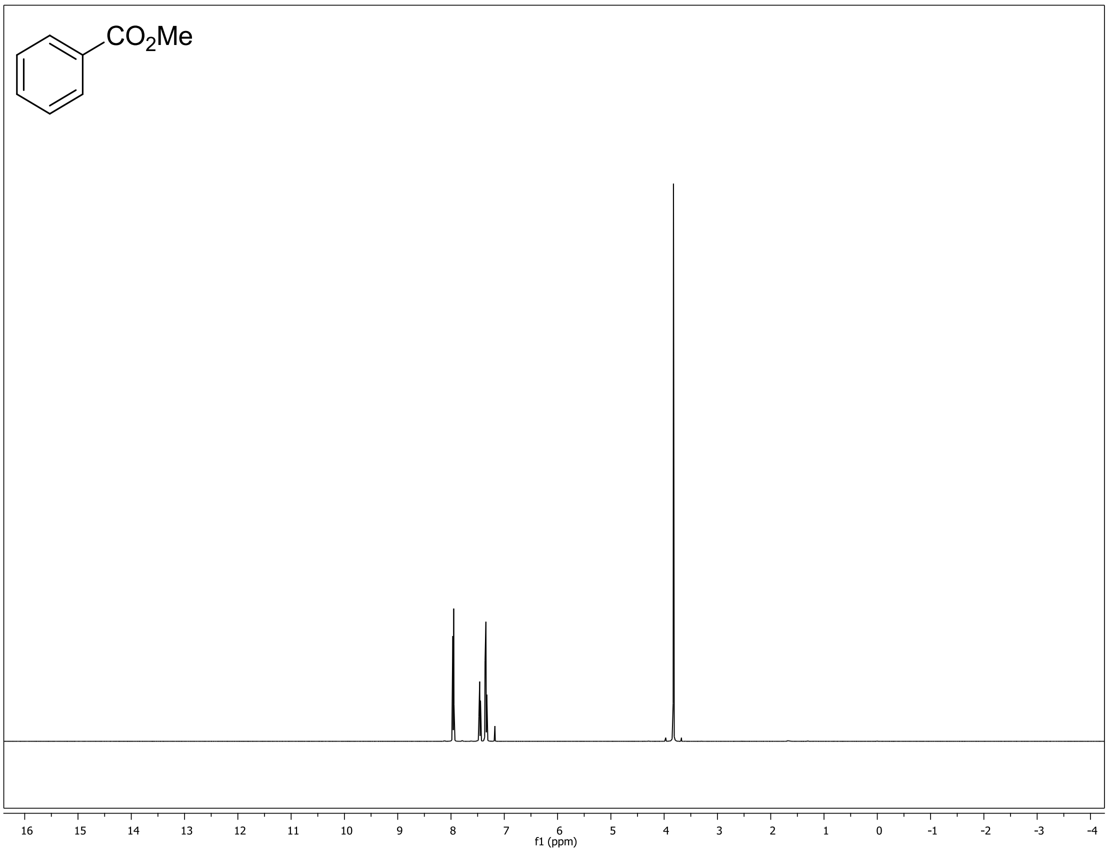
CO2Me
16 15 14 13 12 11 10 9 8 7 6 5 4 3 2 1 0 -1 -2 -3 -4
f1 (ppm)

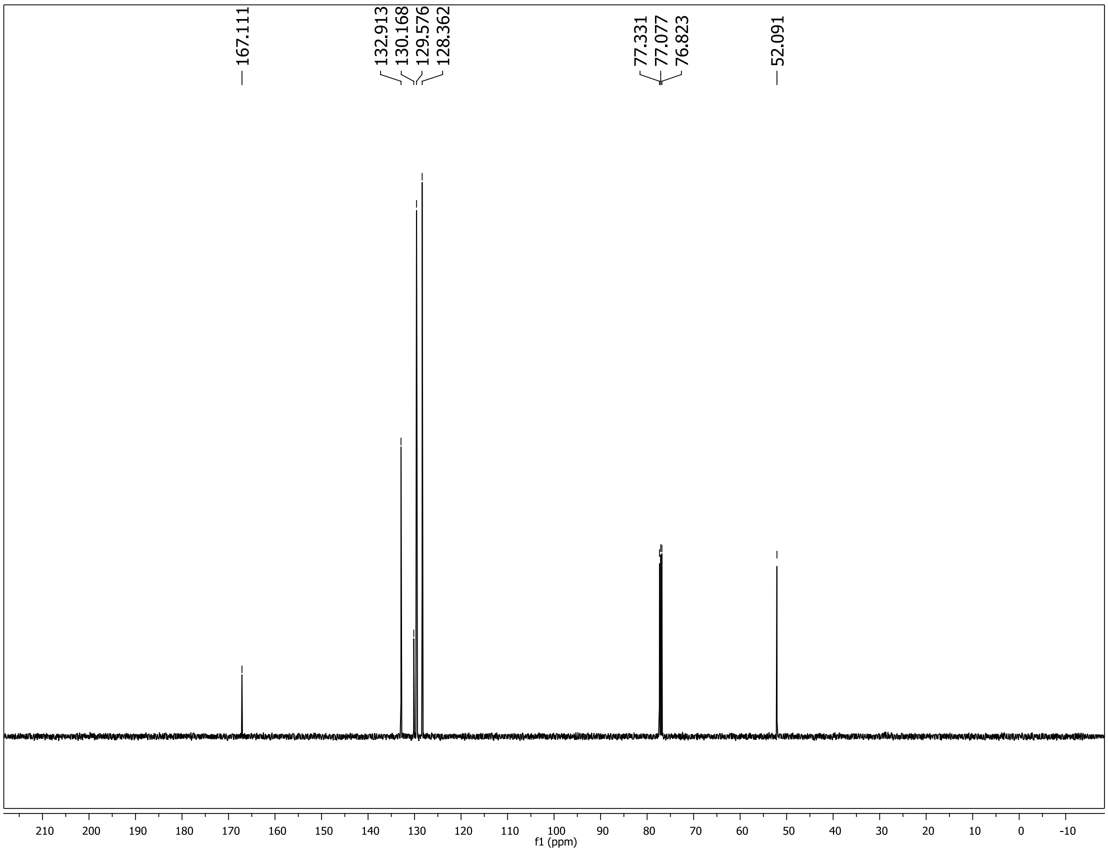
167.111
132.913
130.168
129.576
128.362
77.331
77.077
76.823
52.091
210 200 190 180 170 160 150 140 130 120 110 100 90 80 70 60 50 40 30 20 10 0 -10
f1 (ppm)

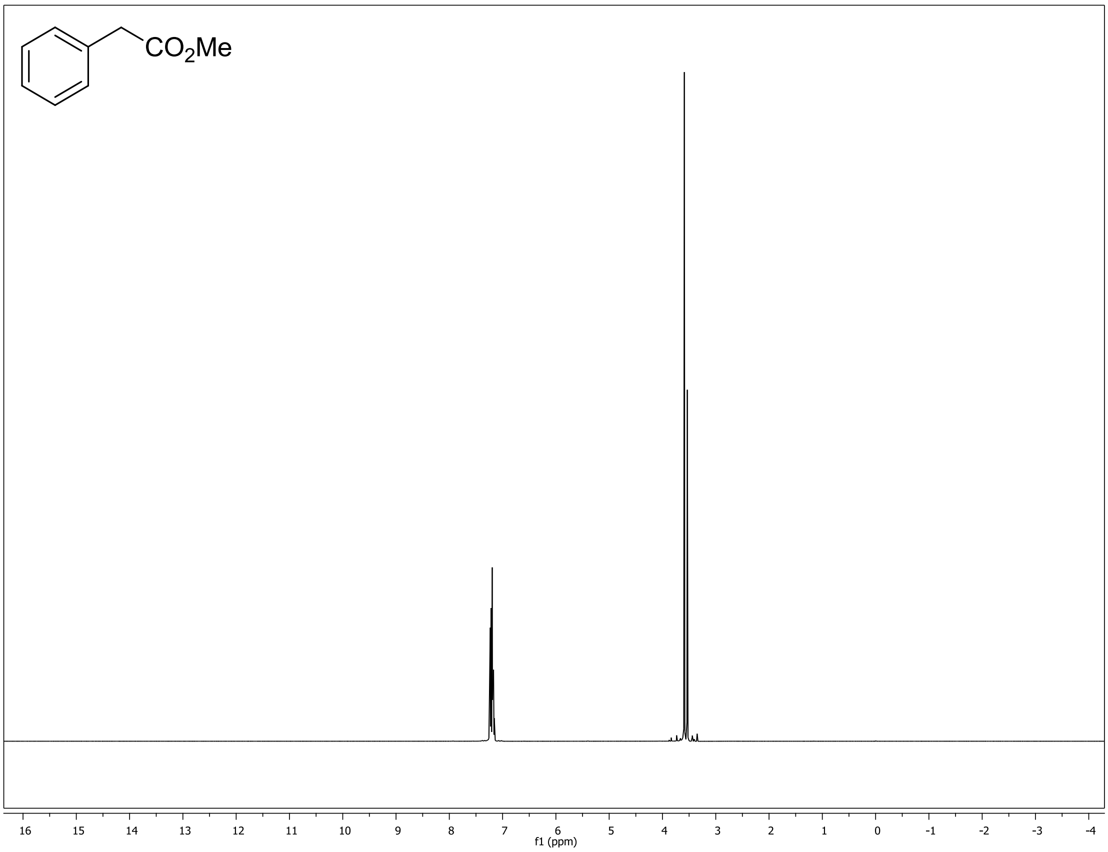
CO2Me
16
15
14
13
12
11
10
9
8
7
6
5
4
3
2
1
0
-1
-2
-3
-4
f1 (ppm)

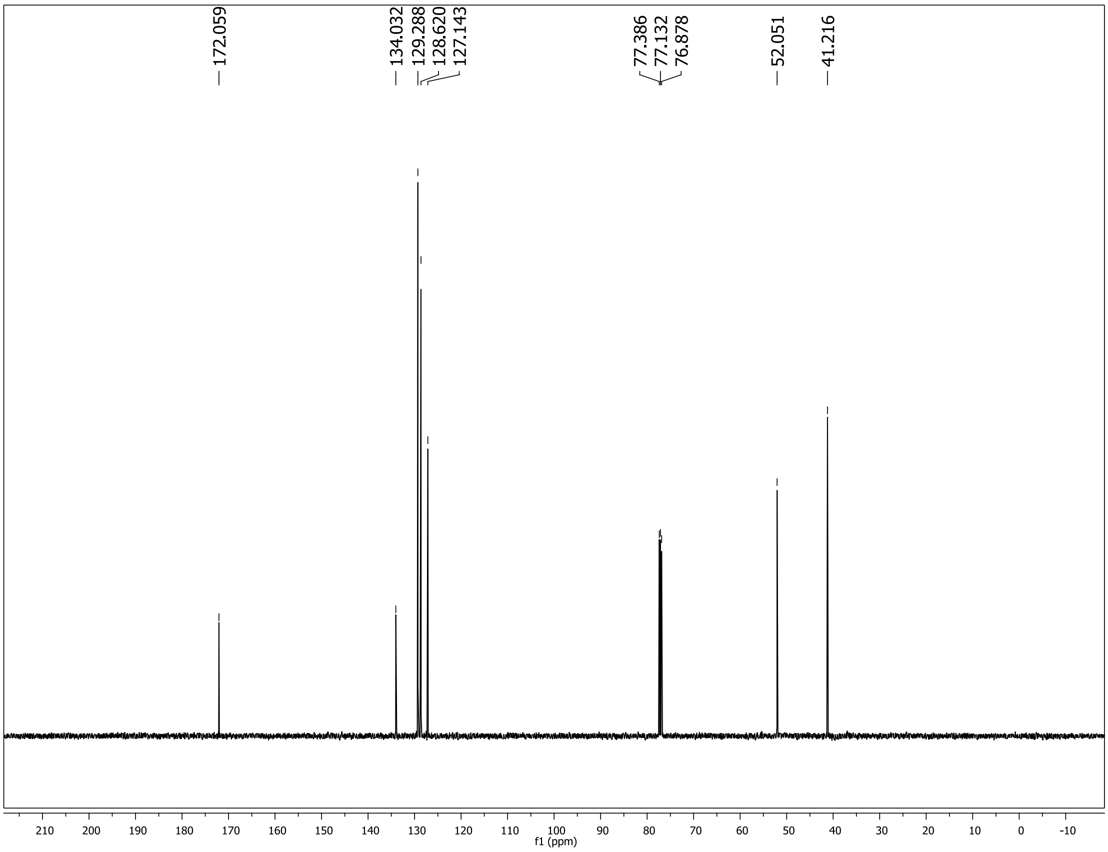
172.059
134.032
129.288
128.620
127.143
77.386
77.132
76.878
52.051
41.216
210
200
190
180
170
160
150
140
130
120
110
100
90
80
70
60
50
40
30
20
10
0
-10
f1 (ppm)

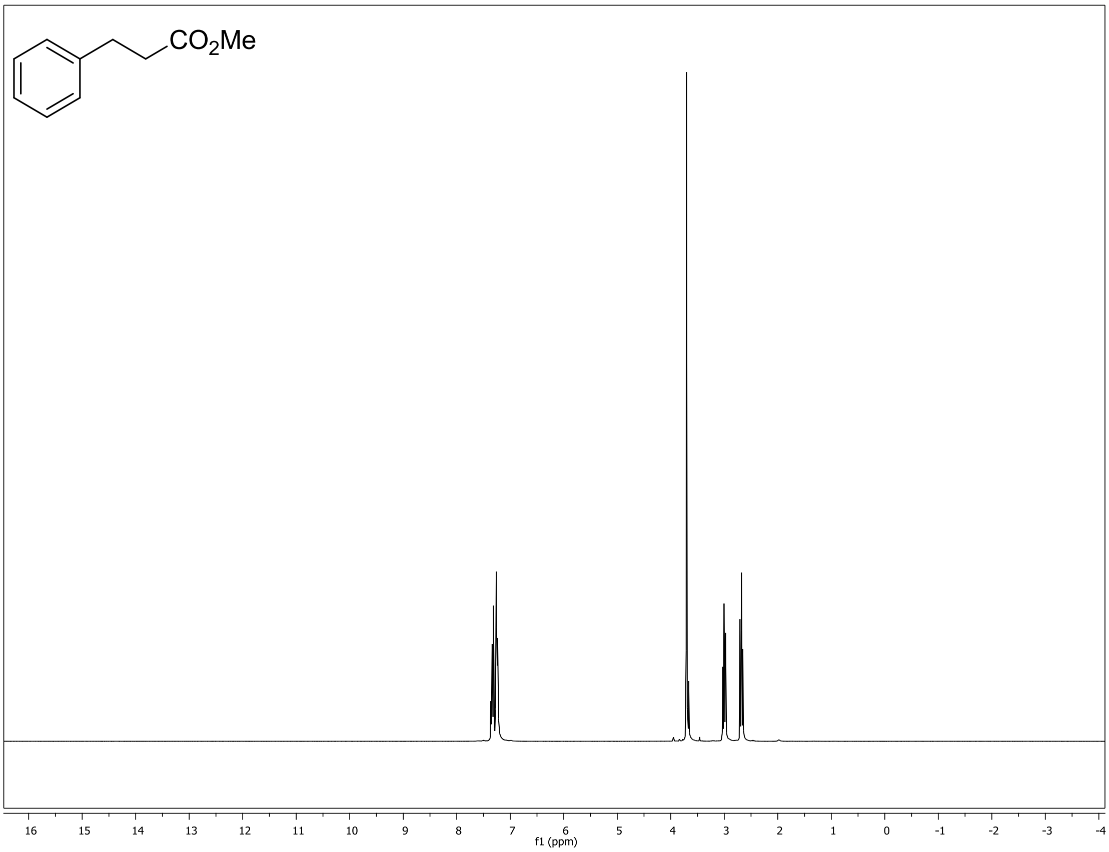
CO2Me
16 15 14 13 12 11 10 9 8 7 6 5 4 3 2 1 0 -1 -2 -3 -4
f1 (ppm)

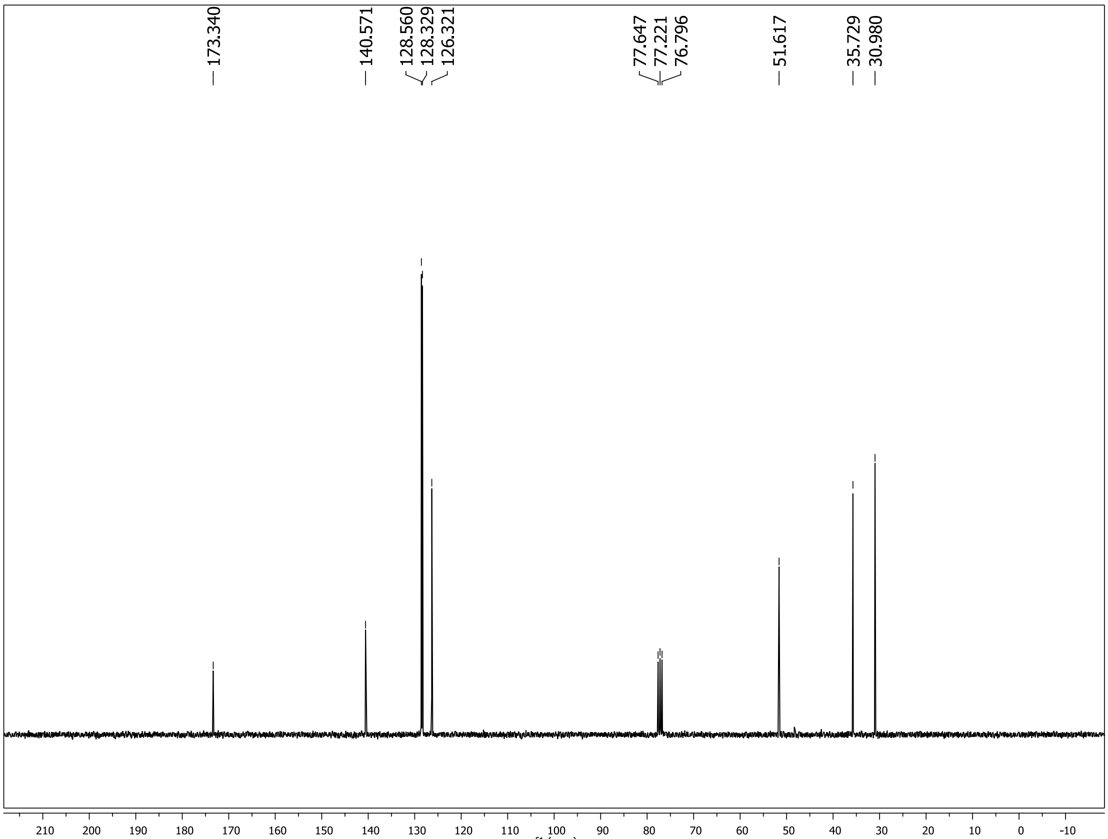
173.340
140.571
128.560
128.329
126.321
77.647
77.221
76.796
51.617
35.729
30.980
210 200 190 180 170 160 150 140 130 120 110 100 90 80 70 60 50 40 30 20 10 0 -10
f1 (ppm)

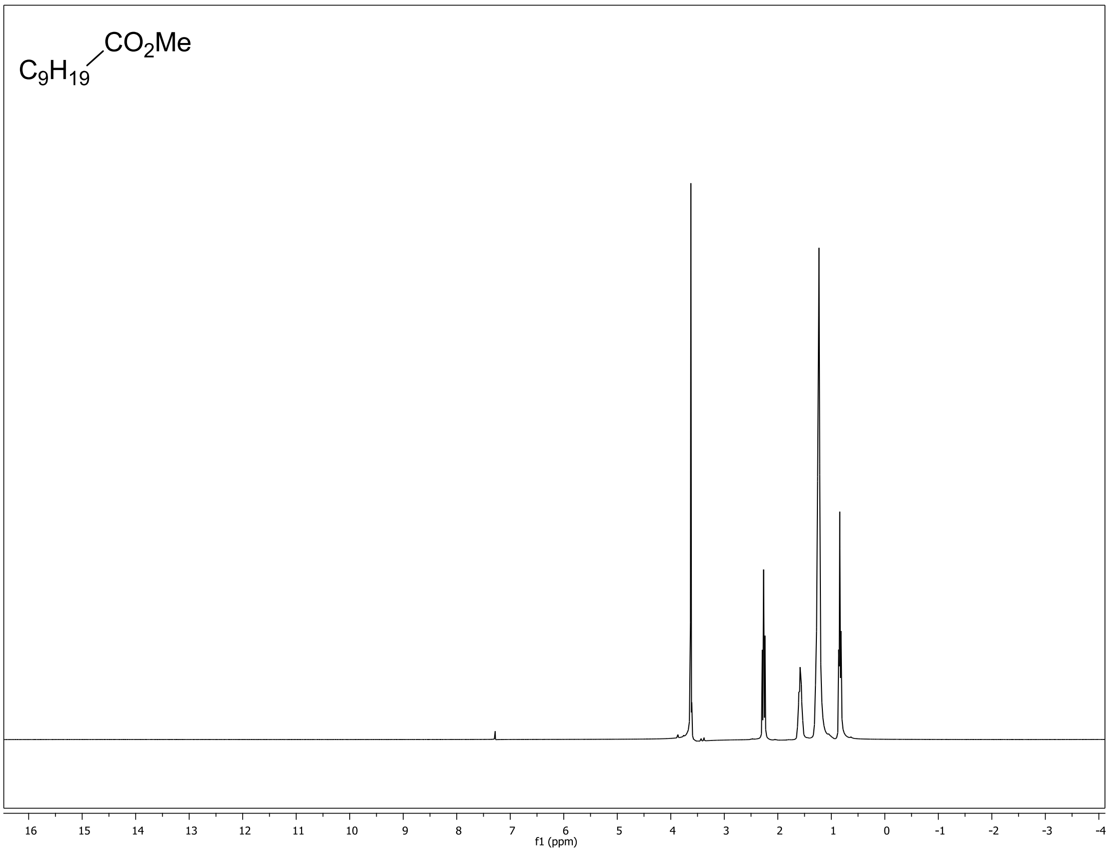
C_9H_{19}–CO_2Me
f1 (ppm)

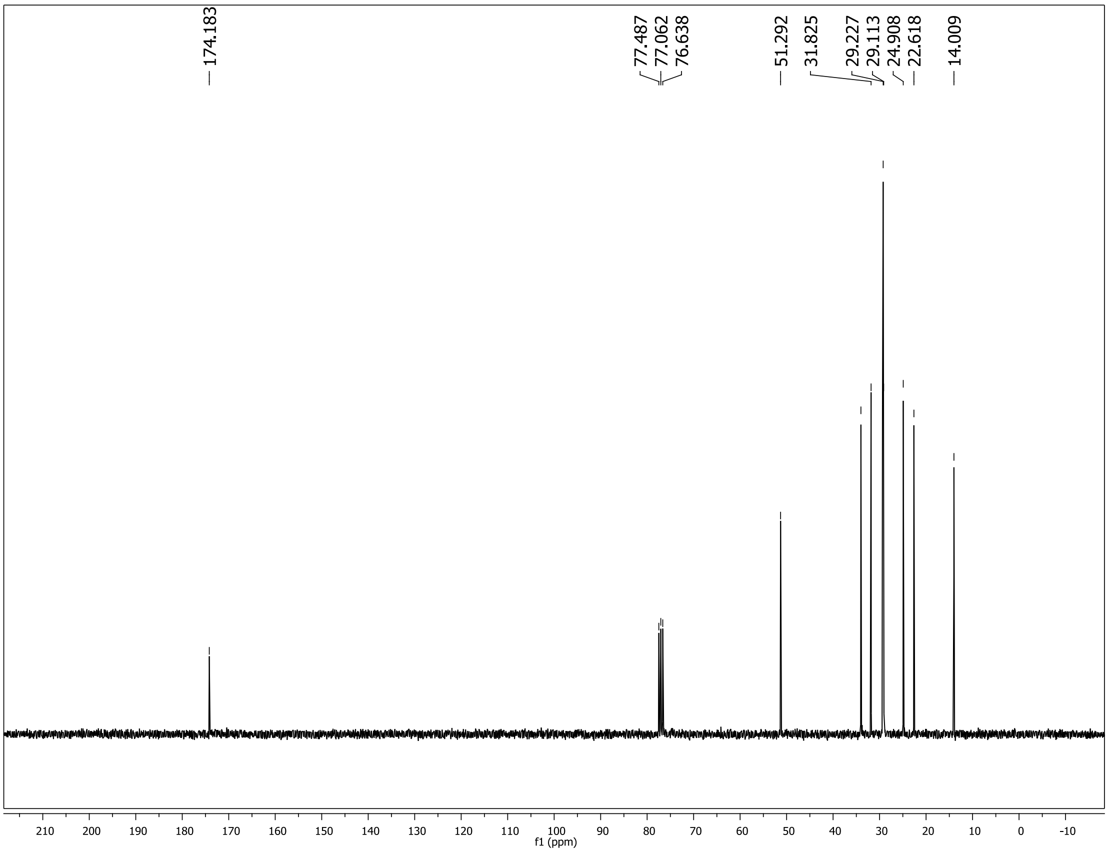
174.183
77.487
77.062
76.638
51.292
31.825
29.227
29.113
24.908
22.618
14.009
f1 (ppm)

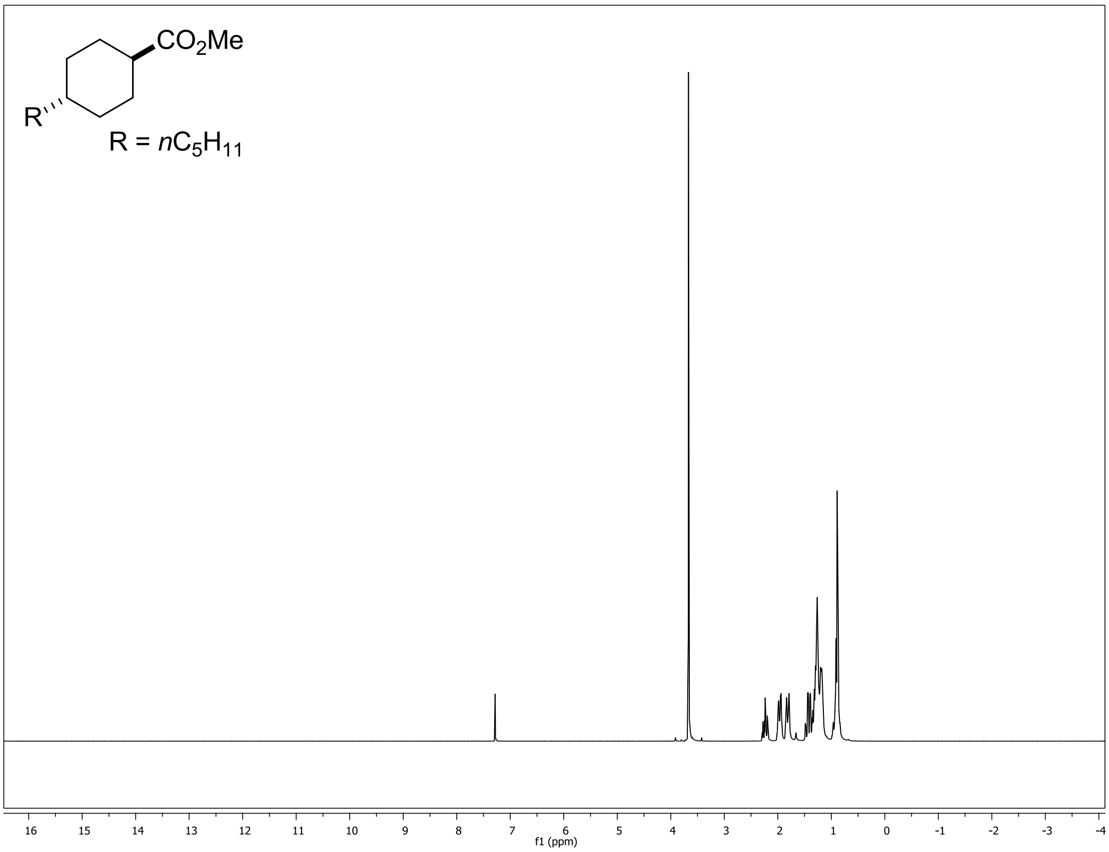
CO2Me
R
R = nC5H11
16
15
14
13
12
11
10
9
8
7
6
5
4
3
2
1
0
-1
-2
-3
-4
f1 (ppm)

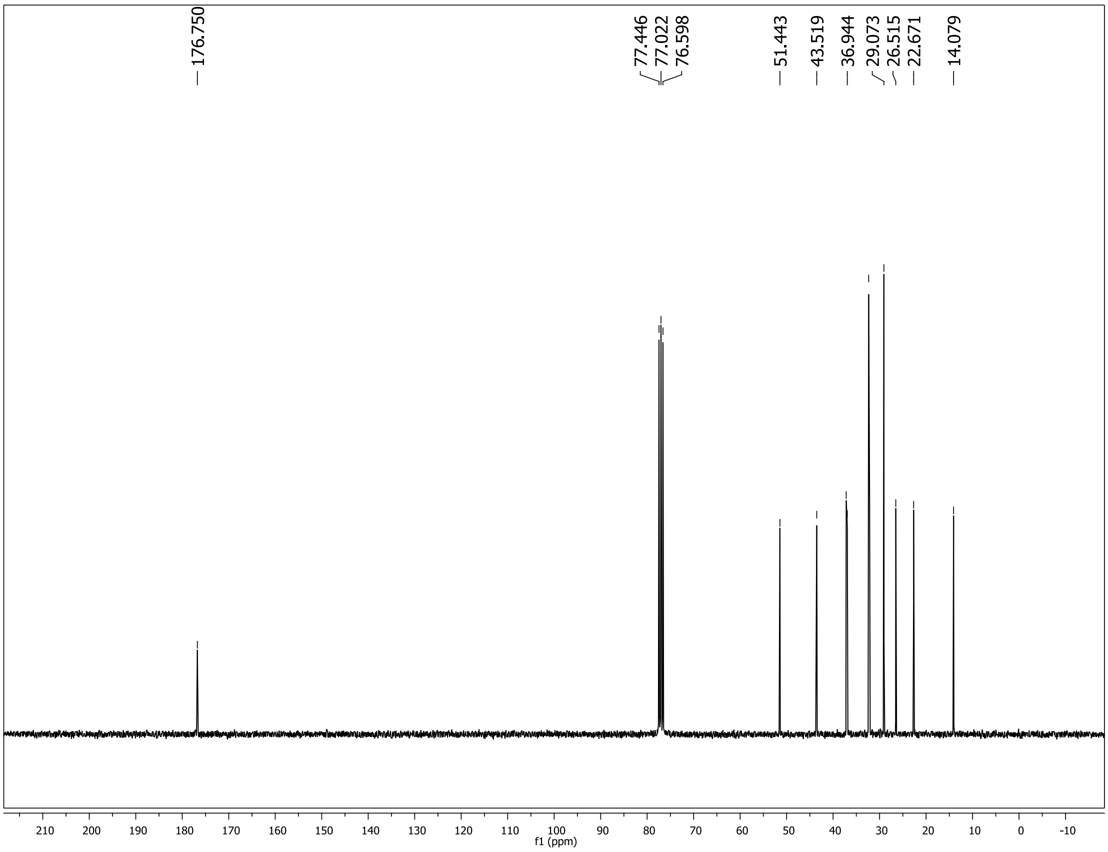
176.750
77.446
77.022
76.598
51.443
43.519
36.944
29.073
26.515
22.671
14.079
210
200
190
180
170
160
150
140
130
120
110
100
90
80
70
60
50
40
30
20
10
0
-10
f1 (ppm)

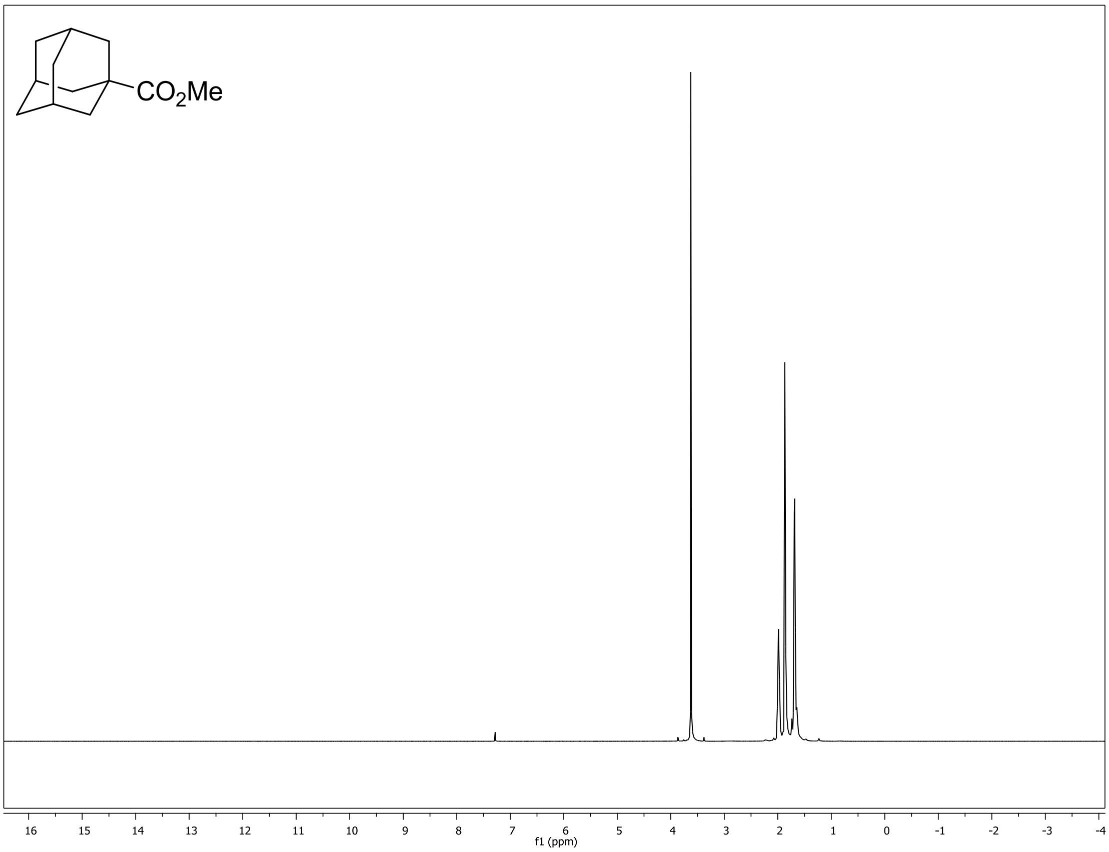
CO2Me
16 15 14 13 12 11 10 9 8 7 6 5 4 3 2 1 0 -1 -2 -3 -4
f1 (ppm)

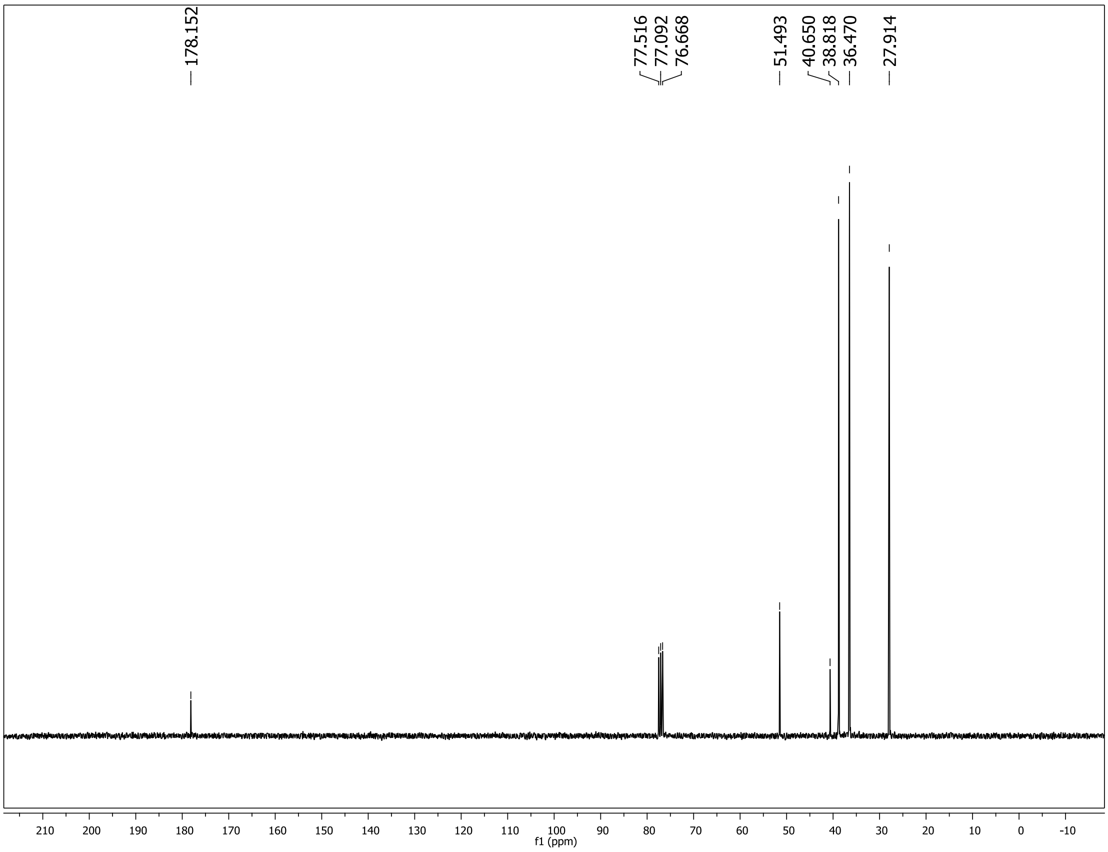
178.152
77.516
77.092
76.668
51.493
40.650
38.818
36.470
27.914
210 200 190 180 170 160 150 140 130 120 110 100 90 80 70 60 50 40 30 20 10 0 -10
f1 (ppm)

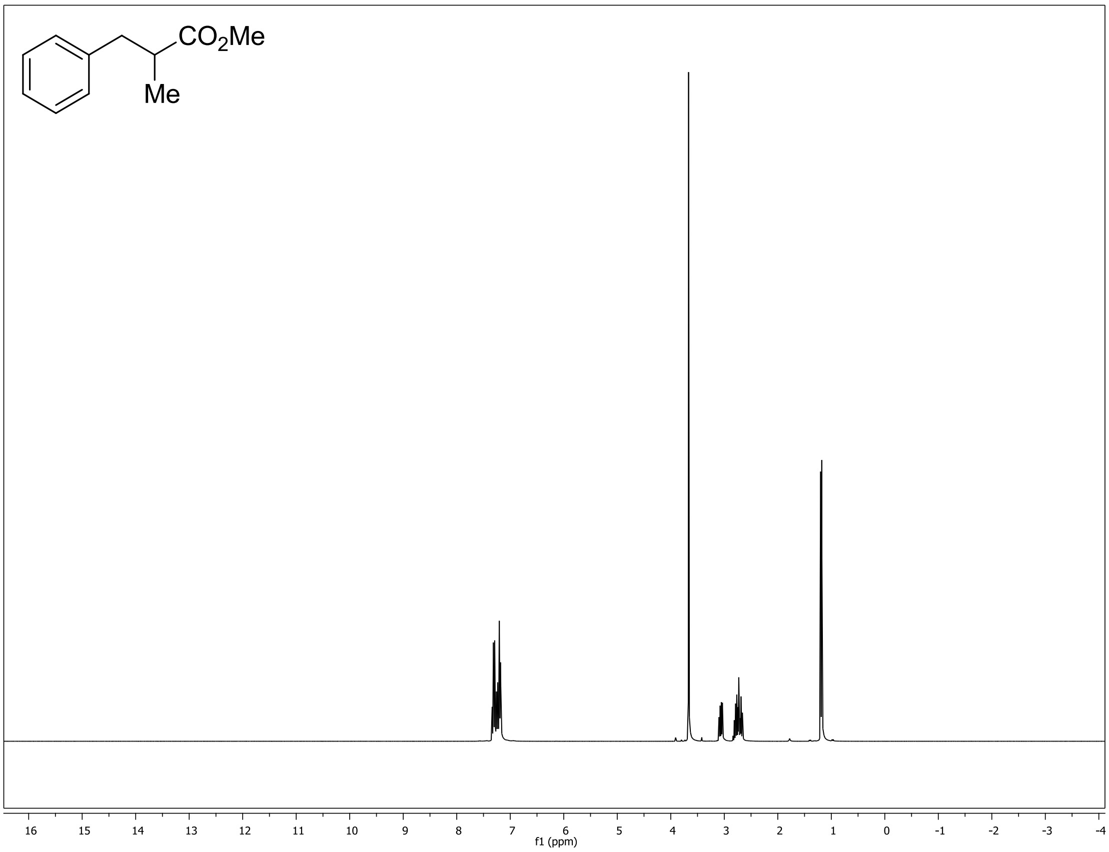
CO2Me
Me
f1 (ppm)

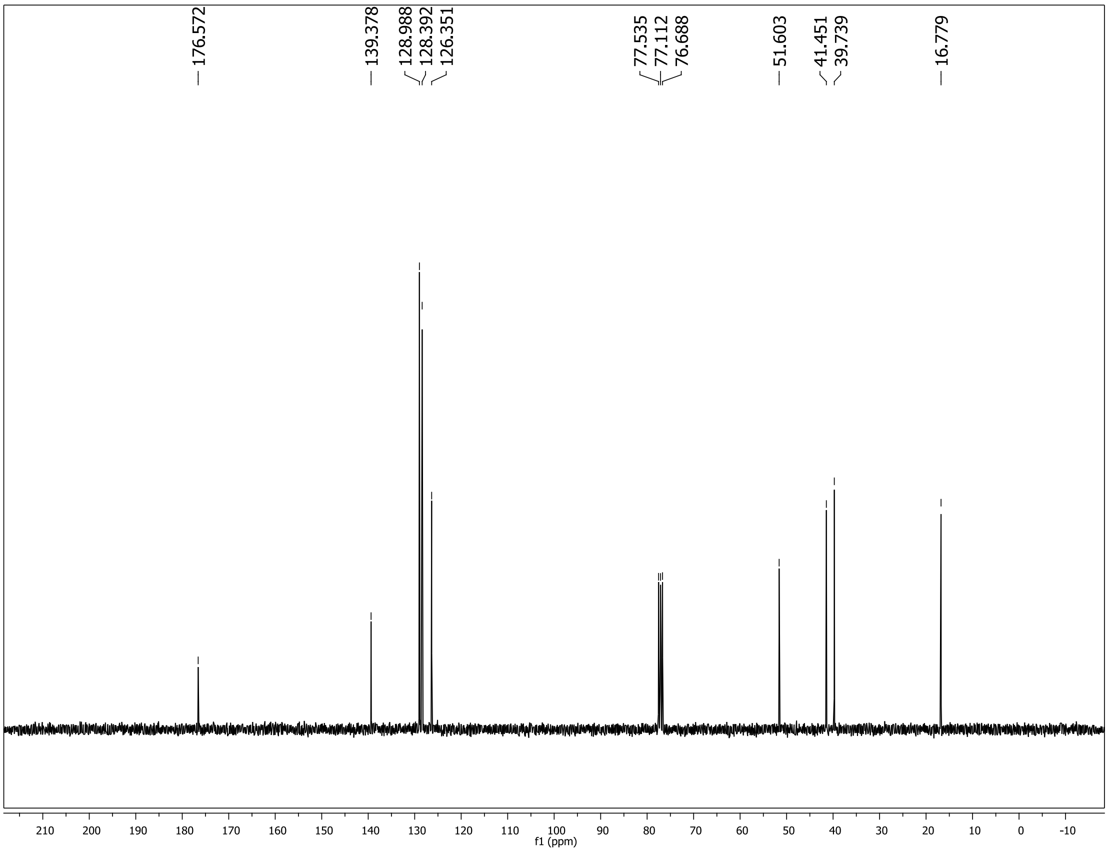
176.572
139.378
128.988
128.392
126.351
77.535
77.112
76.688
51.603
41.451
39.739
16.779
f1 (ppm)

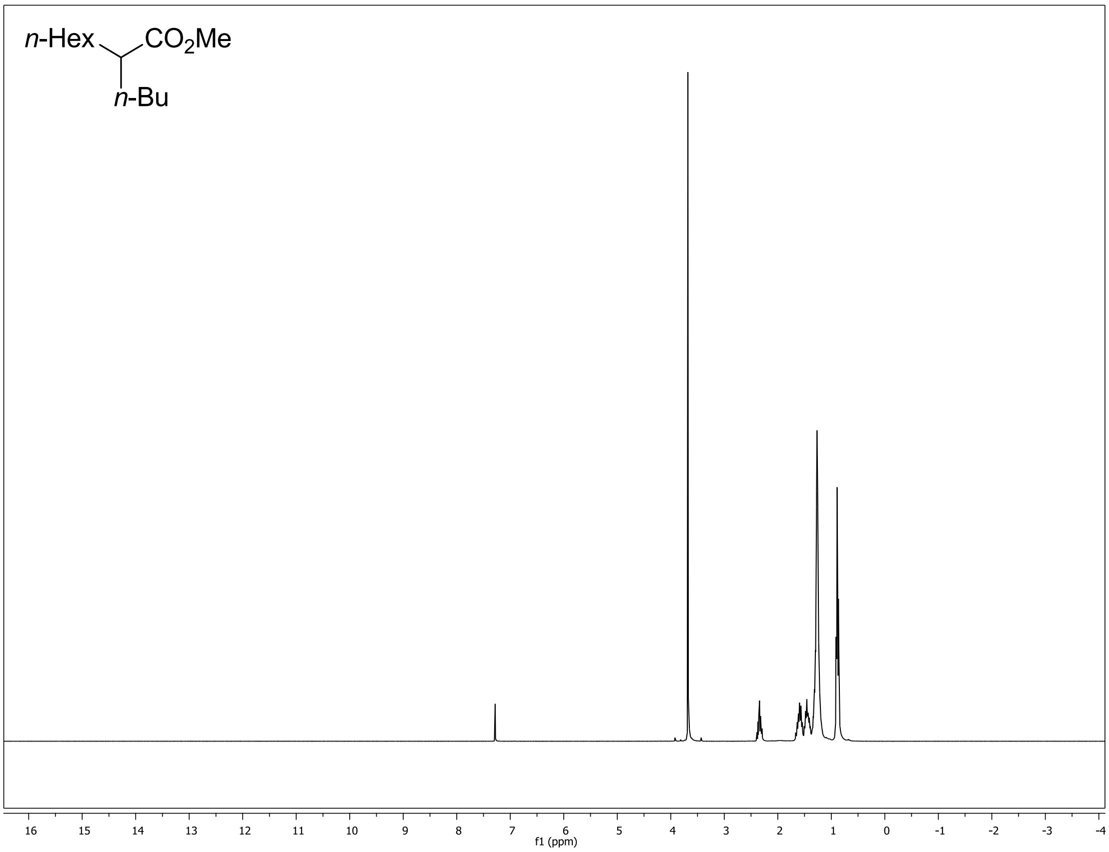
n-Hex
CO2Me
n-Bu
16 15 14 13 12 11 10 9 8 7 6 5 4 3 2 1 0 -1 -2 -3 -4
f1 (ppm)

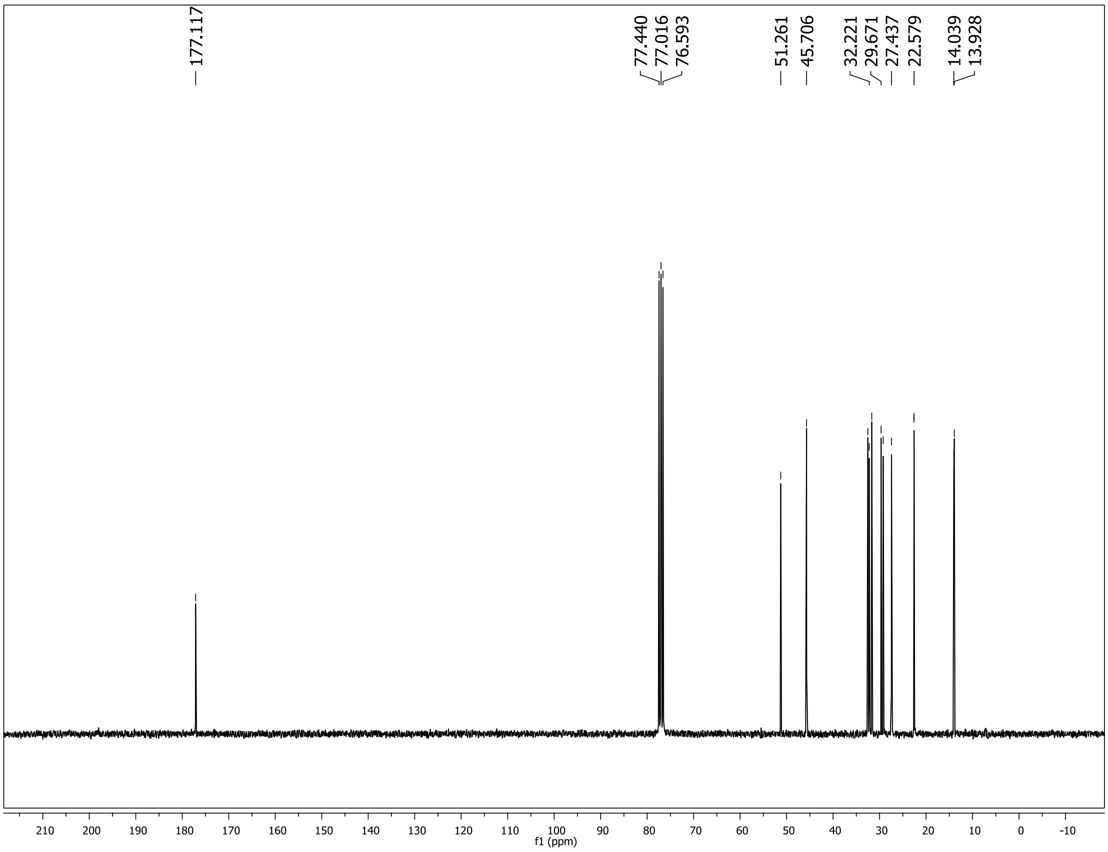
177.117
77.440
77.016
76.593
51.261
45.706
32.221
29.671
27.437
22.579
14.039
13.928
210 200 190 180 170 160 150 140 130 120 110 100 90 80 70 60 50 40 30 20 10 0 -10
f1 (ppm)

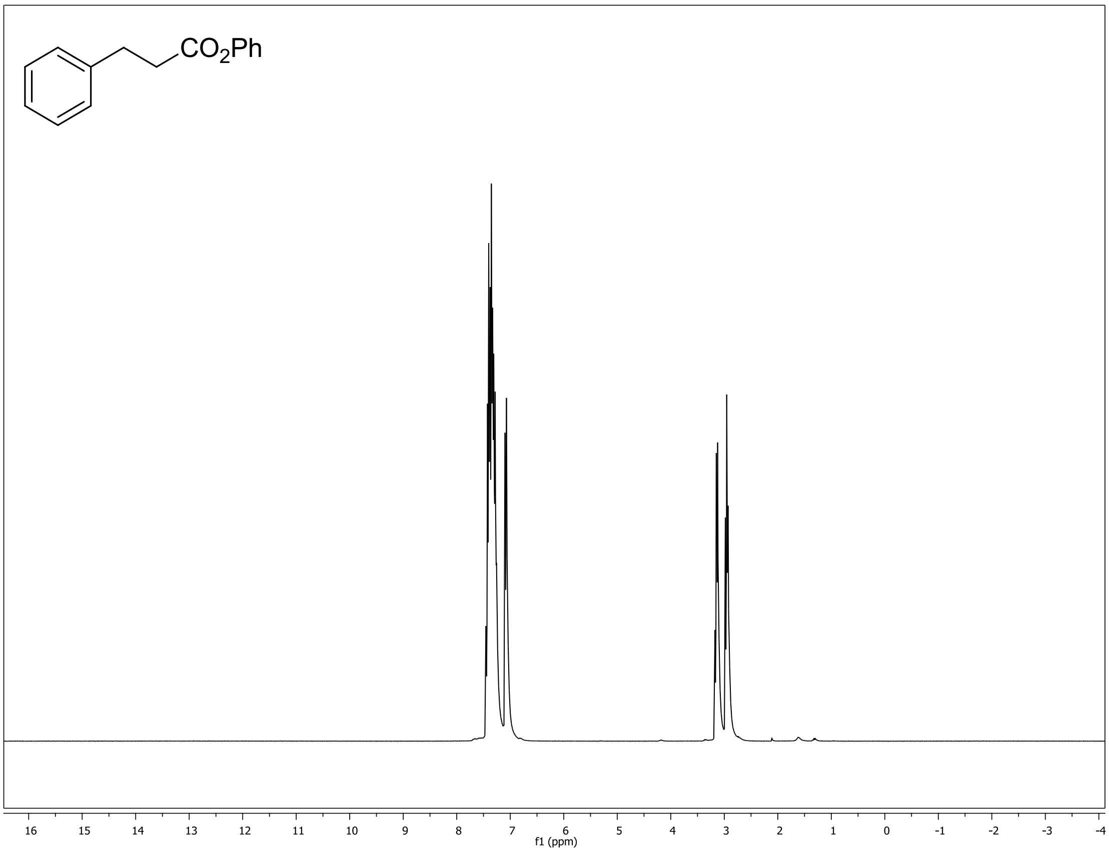
CO2Ph
16 15 14 13 12 11 10 9 8 7 6 5 4 3 2 1 0 -1 -2 -3 -4
f1 (ppm)

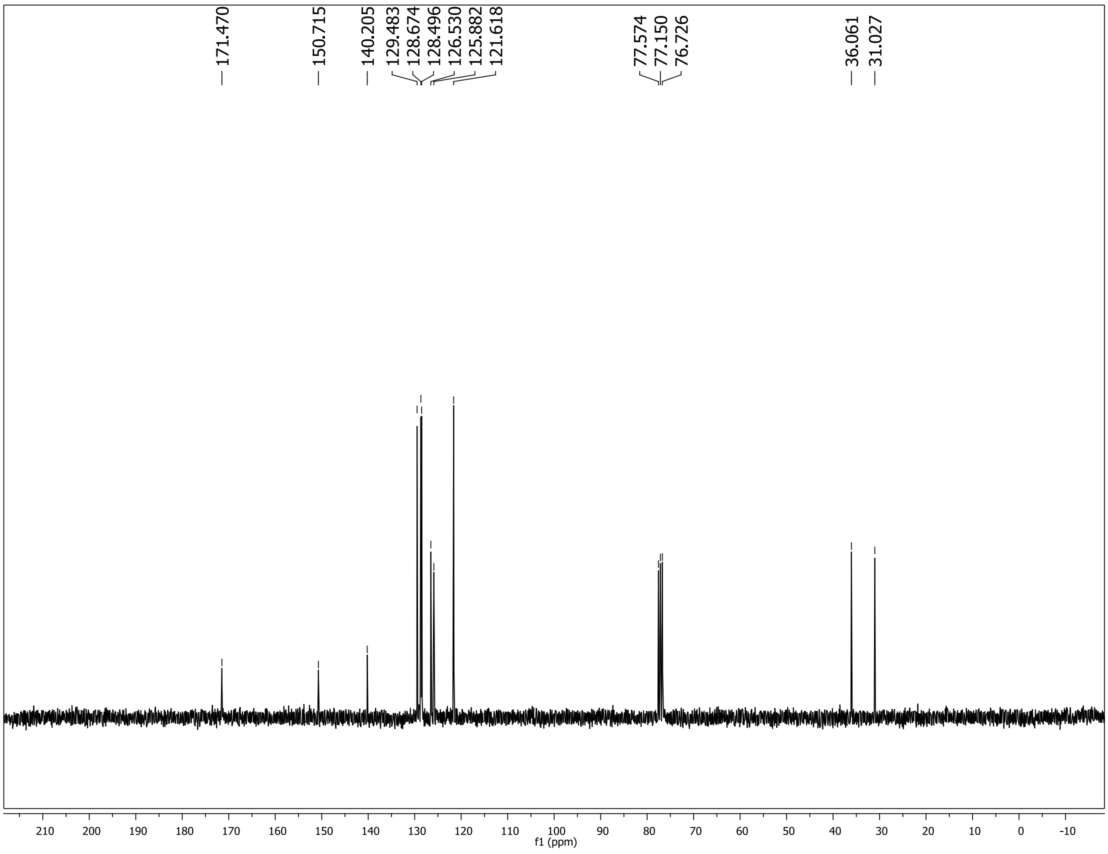
171.470
150.715
140.205
129.483
128.674
128.496
126.530
125.882
121.618
77.574
77.150
76.726
36.061
31.027
210 200 190 180 170 160 150 140 130 120 110 100 90 80 70 60 50 40 30 20 10 0 -10
f1 (ppm)

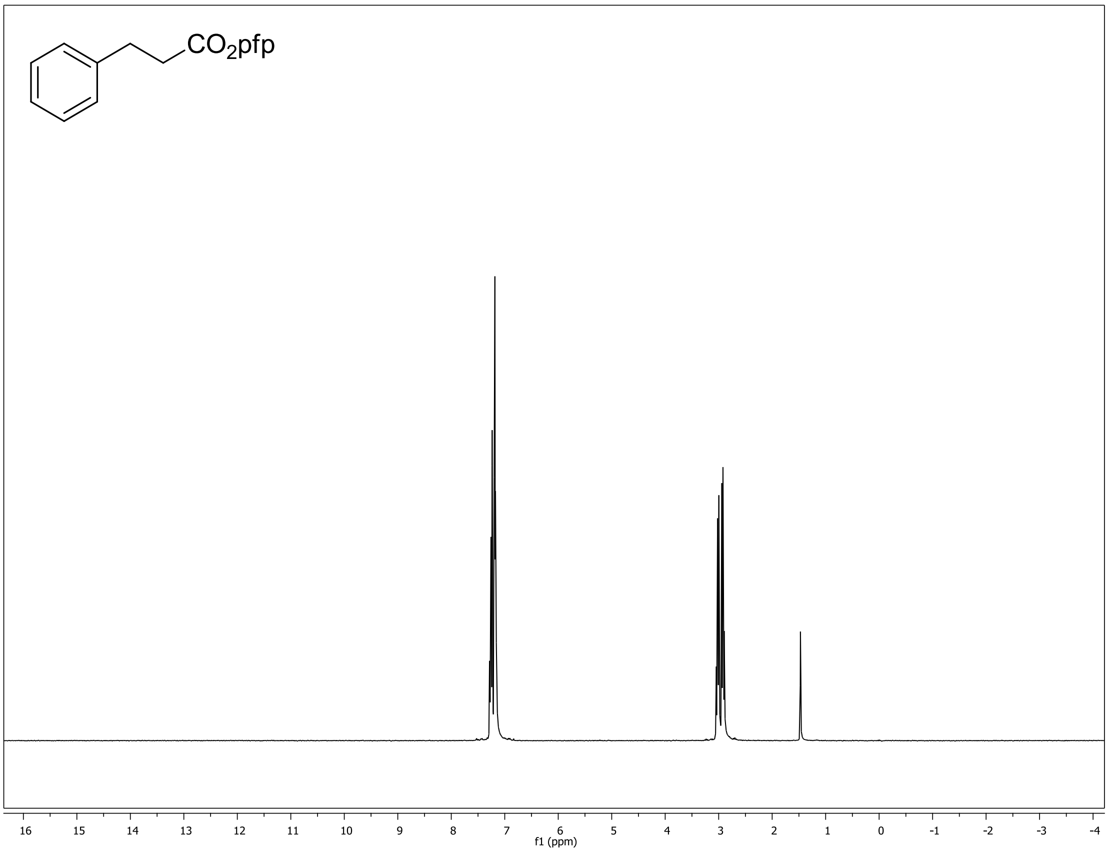
CO2pfp
16 15 14 13 12 11 10 9 8 7 6 5 4 3 2 1 0 -1 -2 -3 -4
f1 (ppm)

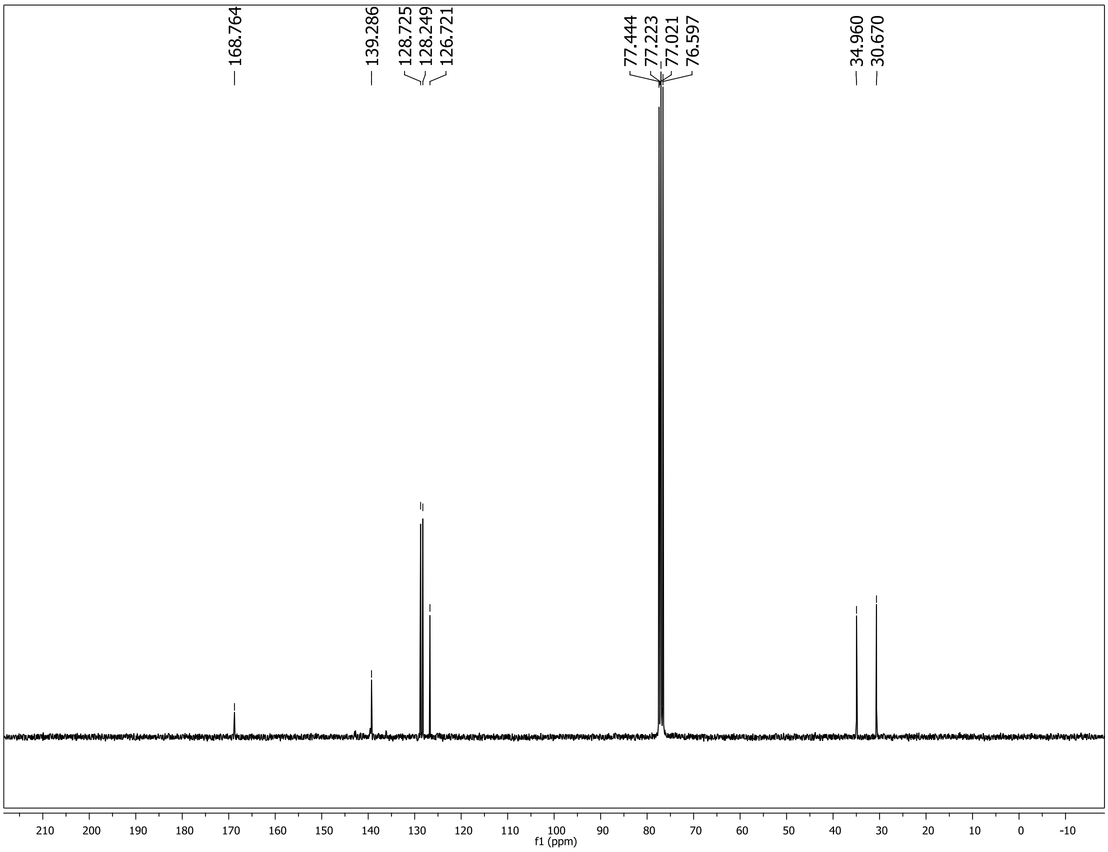
168.764
139.286
128.725
128.249
126.721
77.444
77.223
77.021
76.597
34.960
30.670
210 200 190 180 170 160 150 140 130 120 110 100 90 80 70 60 50 40 30 20 10 0 -10
f1 (ppm)

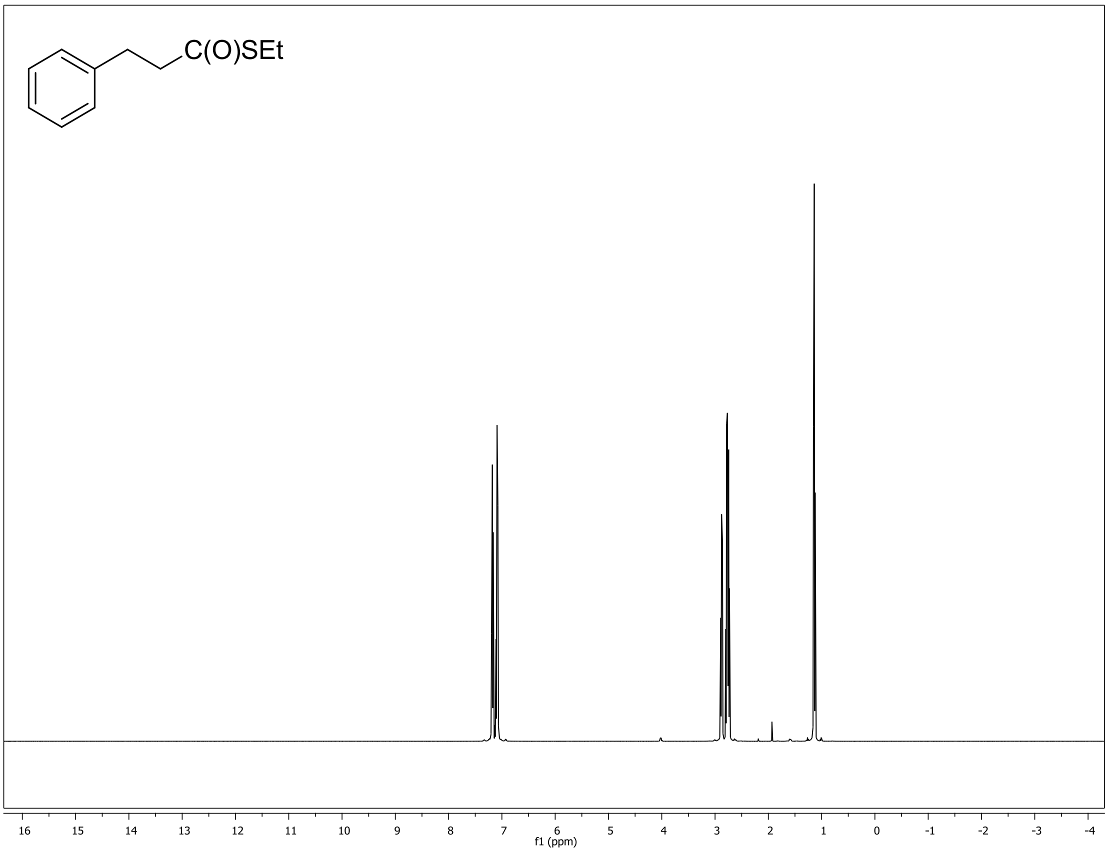
C(O)SEt
16 15 14 13 12 11 10 9 8 7 6 5 4 3 2 1 0 -1 -2 -3 -4
f1 (ppm)

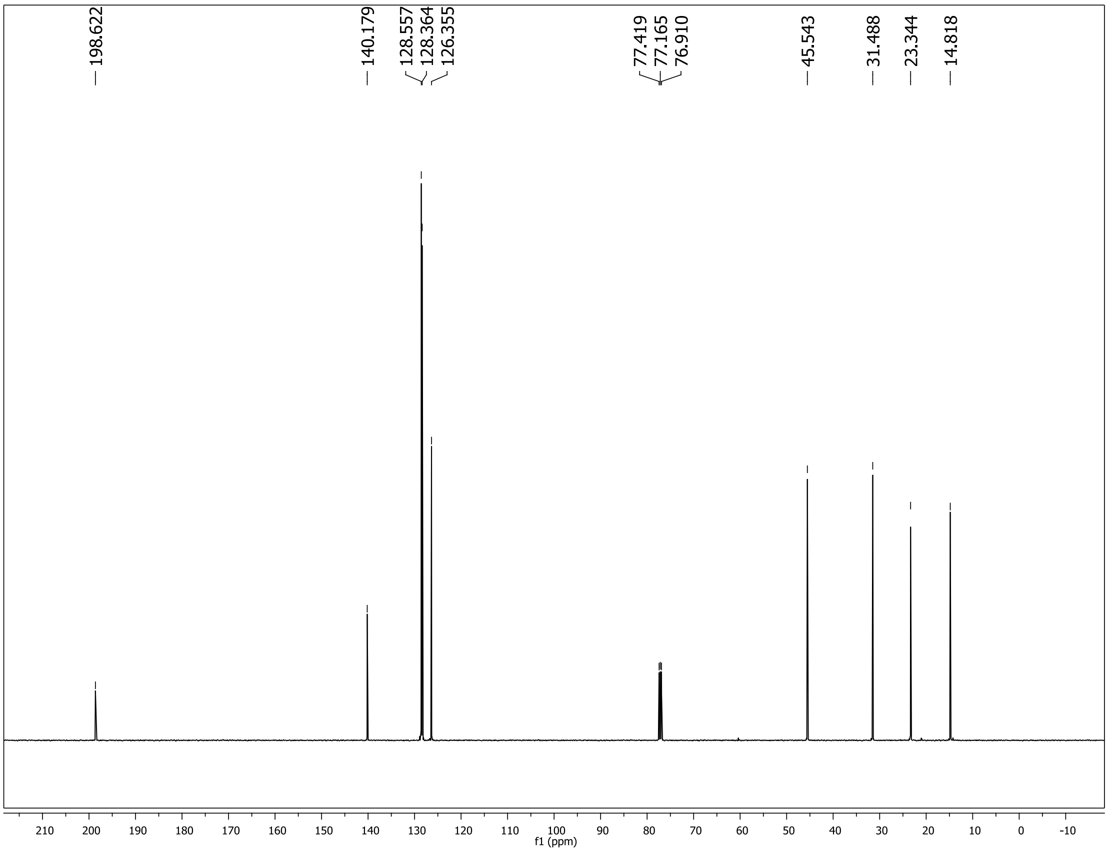
198.622
140.179
128.557
128.364
126.355
77.419
77.165
76.910
45.543
31.488
23.344
14.818
210 200 190 180 170 160 150 140 130 120 110 100 90 80 70 60 50 40 30 20 10 0 -10
f1 (ppm)

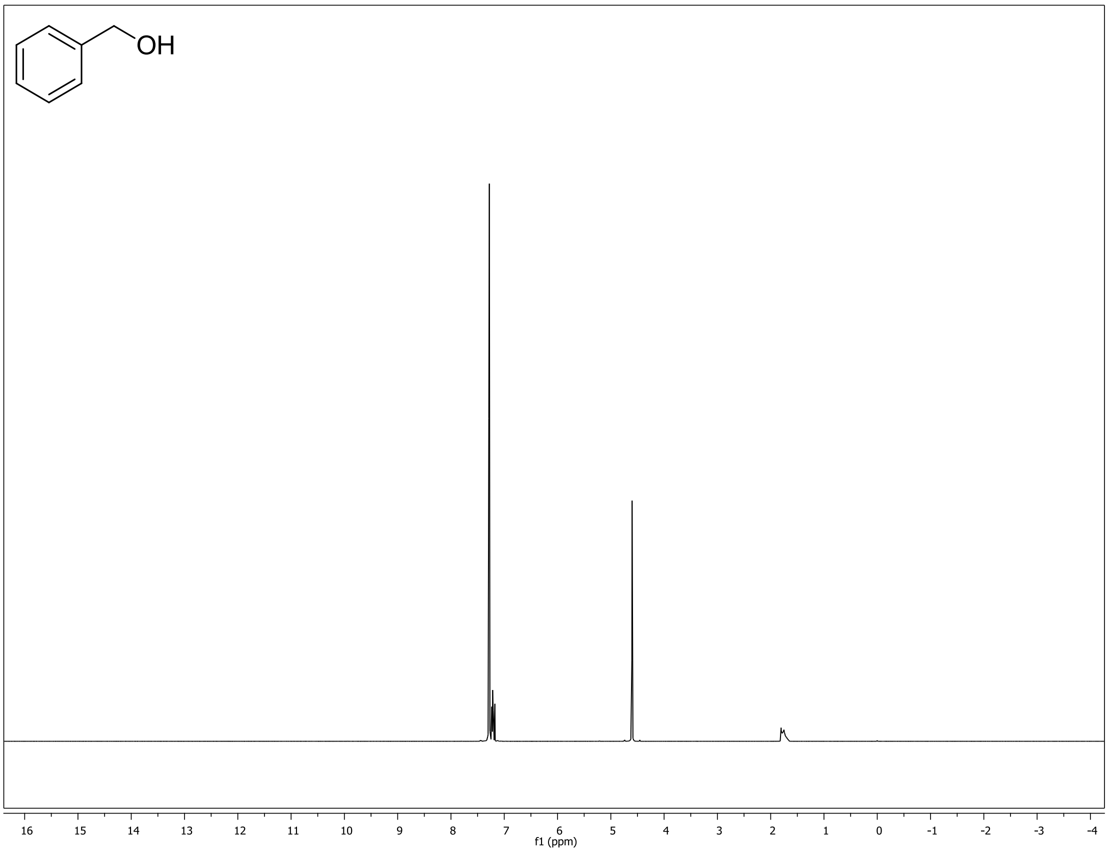
OH
16
15
14
13
12
11
10
9
8
7
6
5
4
3
2
1
0
-1
-2
-3
-4
f1 (ppm)

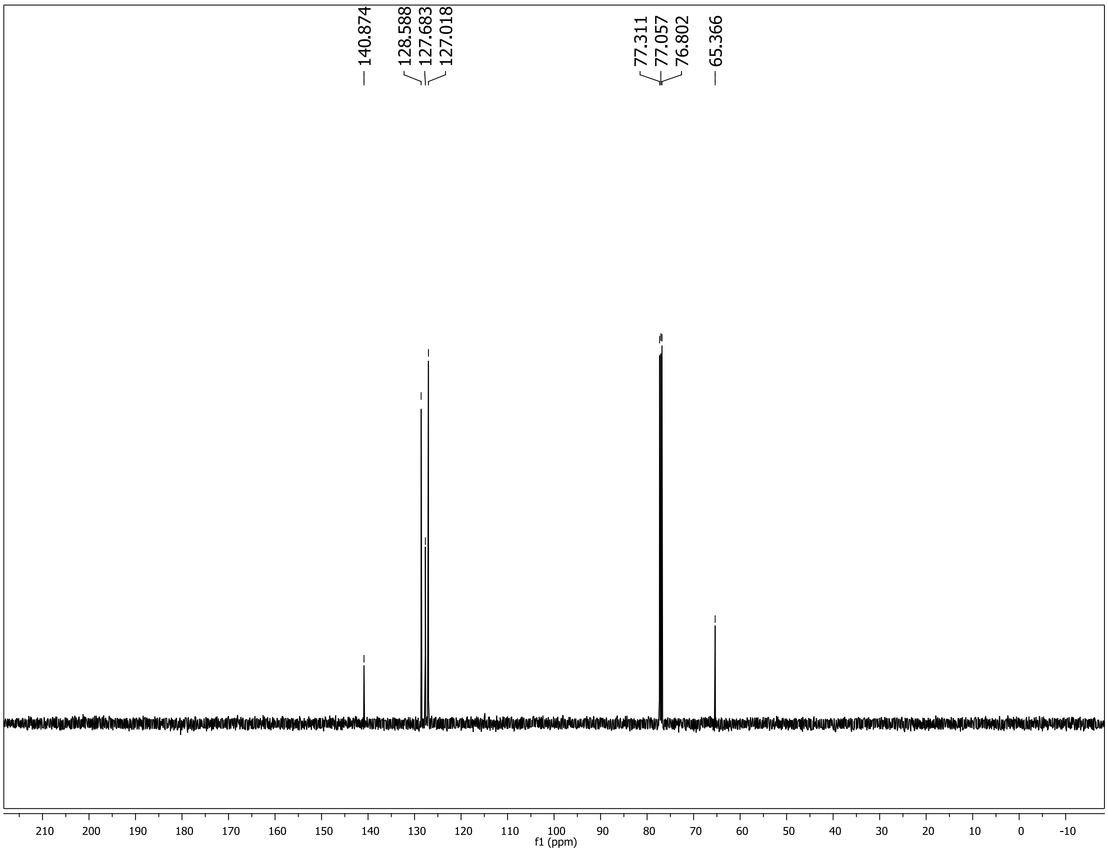
140.874
128.588
127.683
127.018
77.311
77.057
76.802
65.366
210
200
190
180
170
160
150
140
130
120
110
100
90
80
70
60
50
40
30
20
10
0
-10
f1 (ppm)

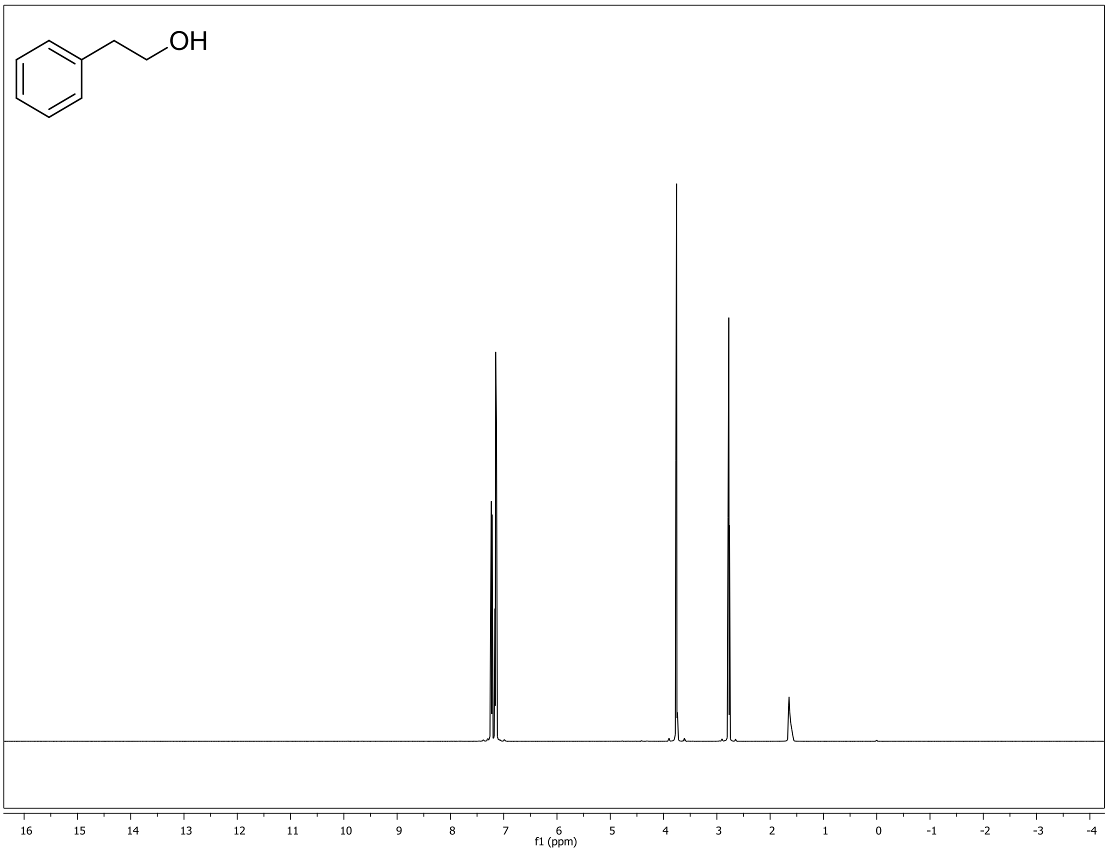
OH
16 15 14 13 12 11 10 9 8 7 6 5 4 3 2 1 0 -1 -2 -3 -4
f1 (ppm)

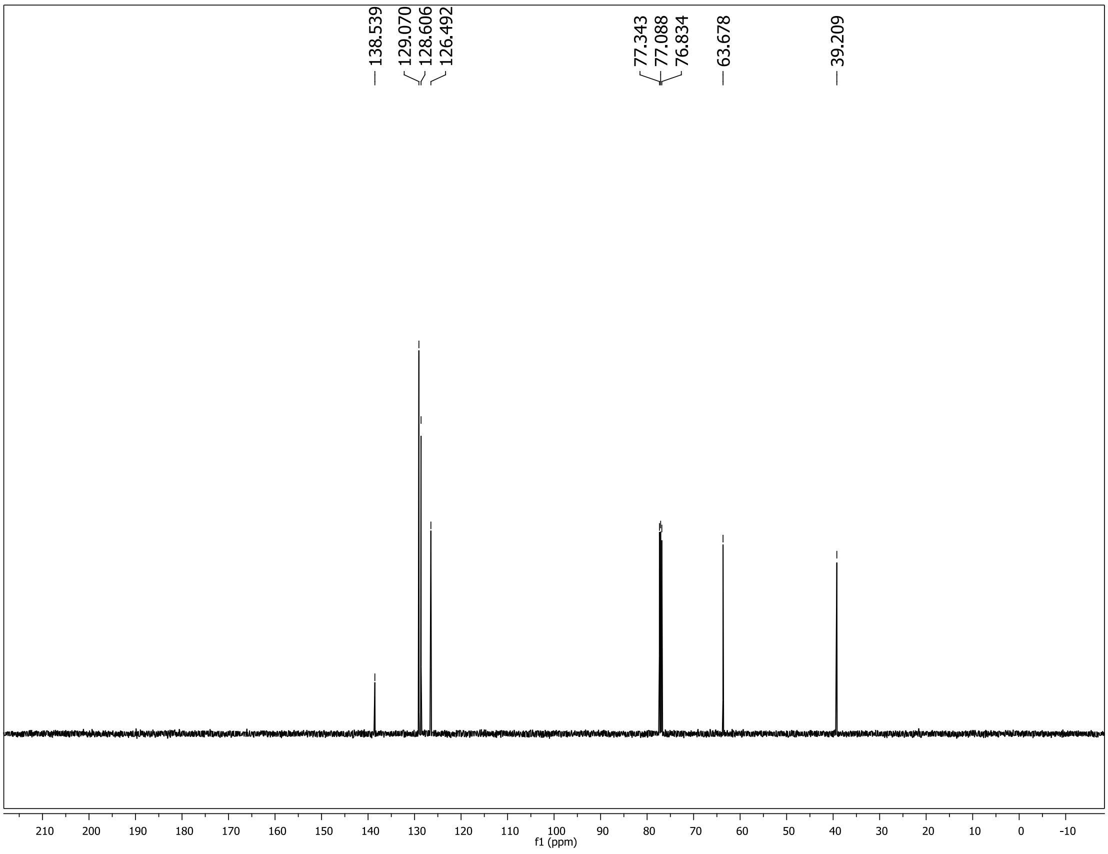
138.539
129.070
128.606
126.492
77.343
77.088
76.834
63.678
39.209
210 200 190 180 170 160 150 140 130 120 110 100 90 80 70 60 50 40 30 20 10 0 -10
f1 (ppm)

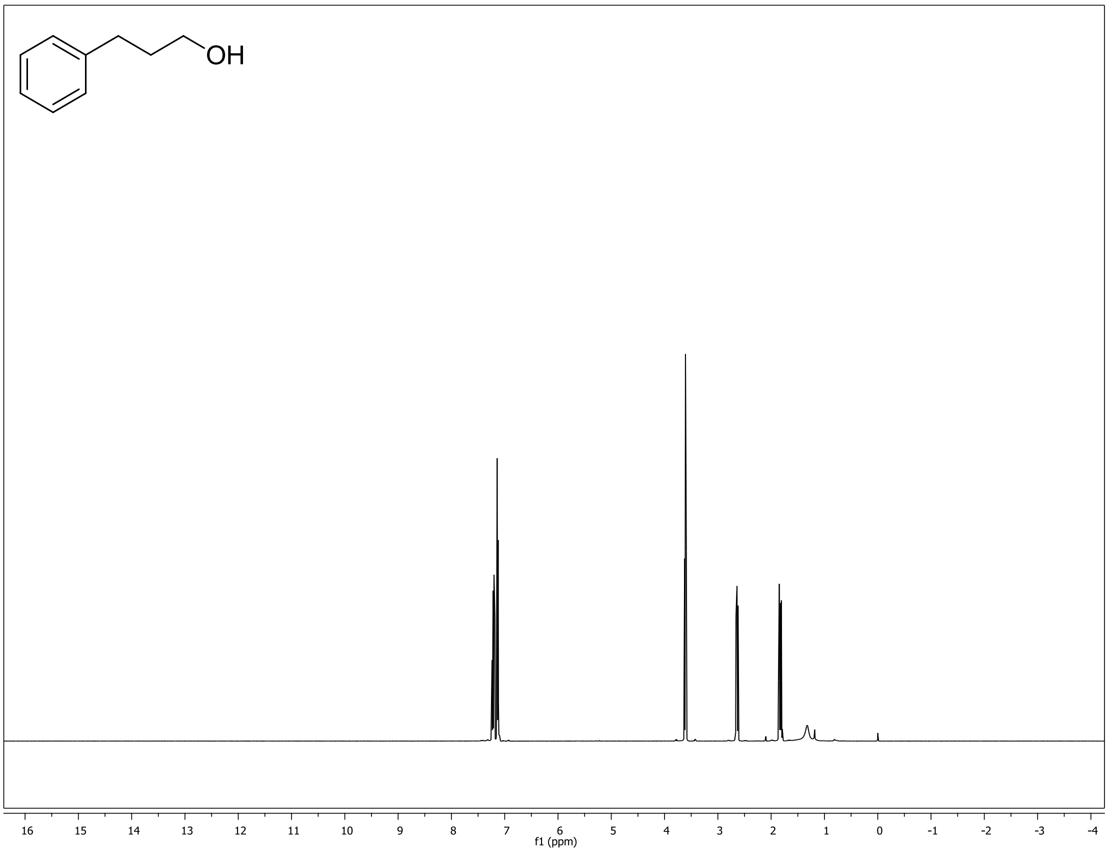
OH
16
15
14
13
12
11
10
9
8
7
6
5
4
3
2
1
0
-1
-2
-3
-4
f1 (ppm)

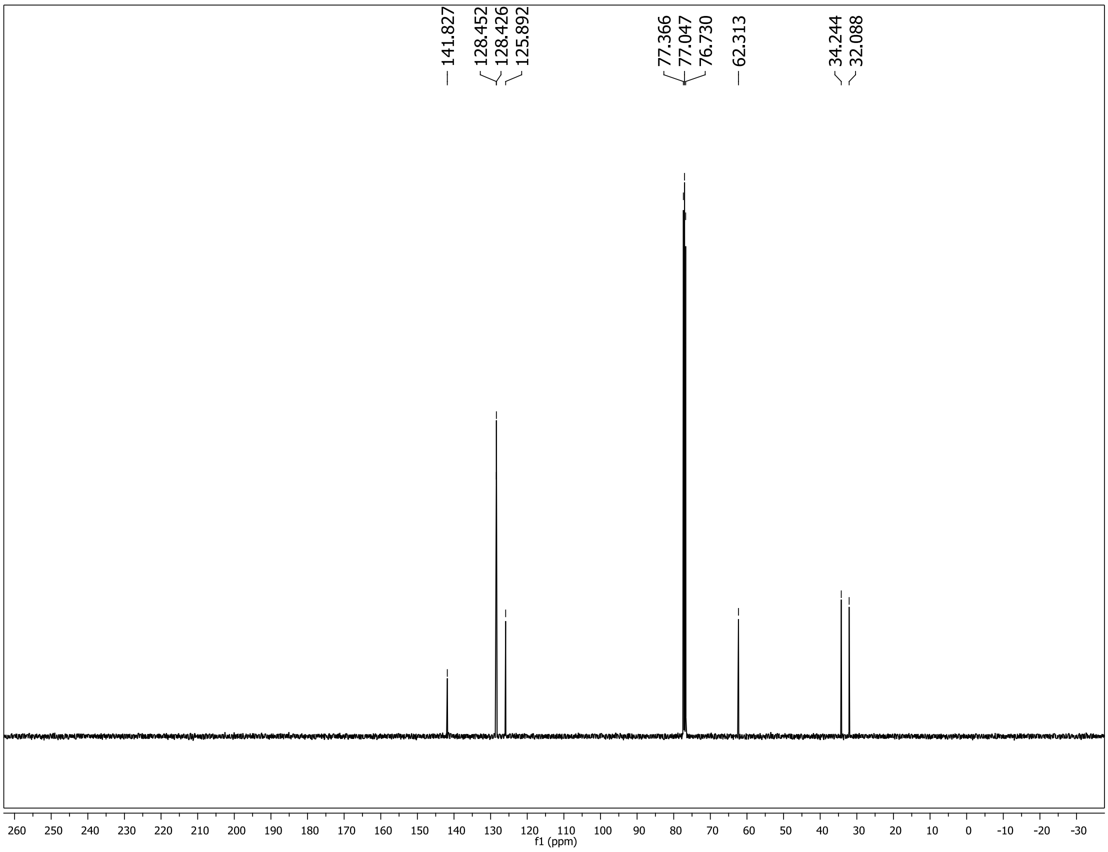
141.827
128.452
128.426
125.892
77.366
77.047
76.730
62.313
34.244
32.088
260
250
240
230
220
210
200
190
180
170
160
150
140
130
120
110
100
90
80
70
60
50
40
30
20
10
0
-10
-20
-30
f1 (ppm)

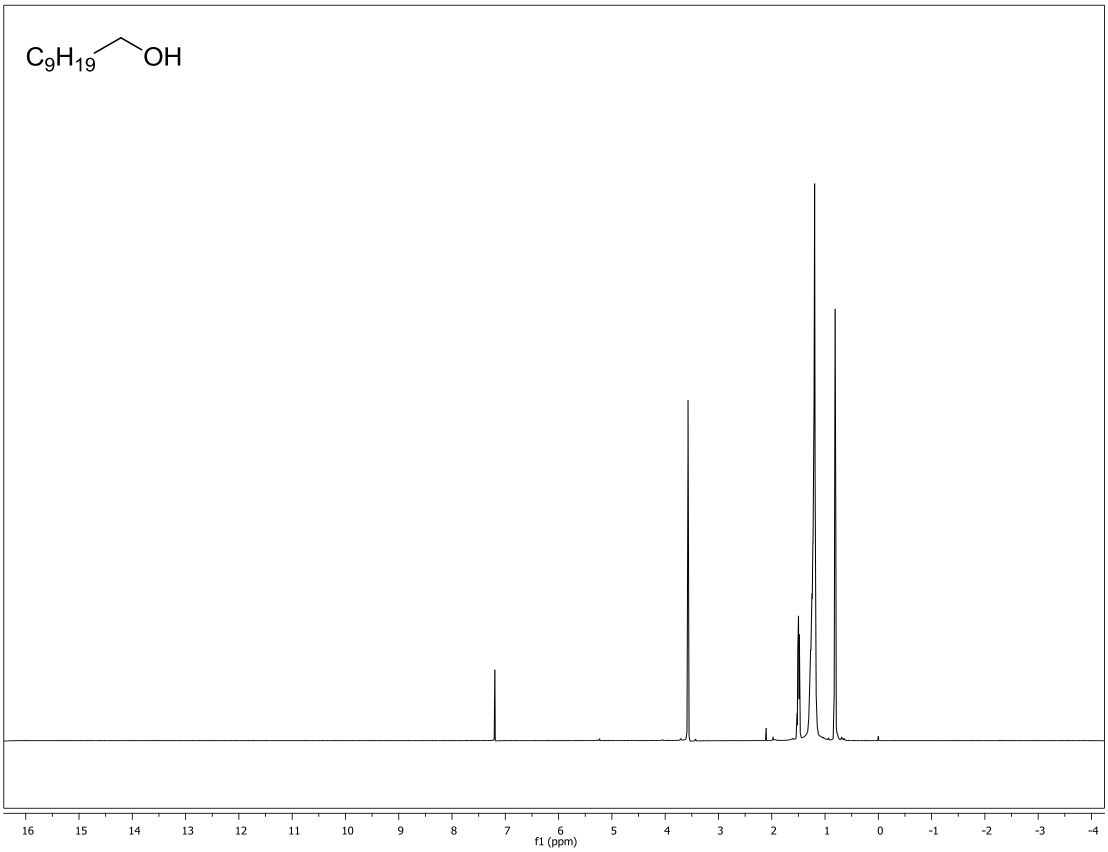
C9H19 OH
16 15 14 13 12 11 10 9 8 7 6 5 4 3 2 1 0 -1 -2 -3 -4
f1 (ppm)

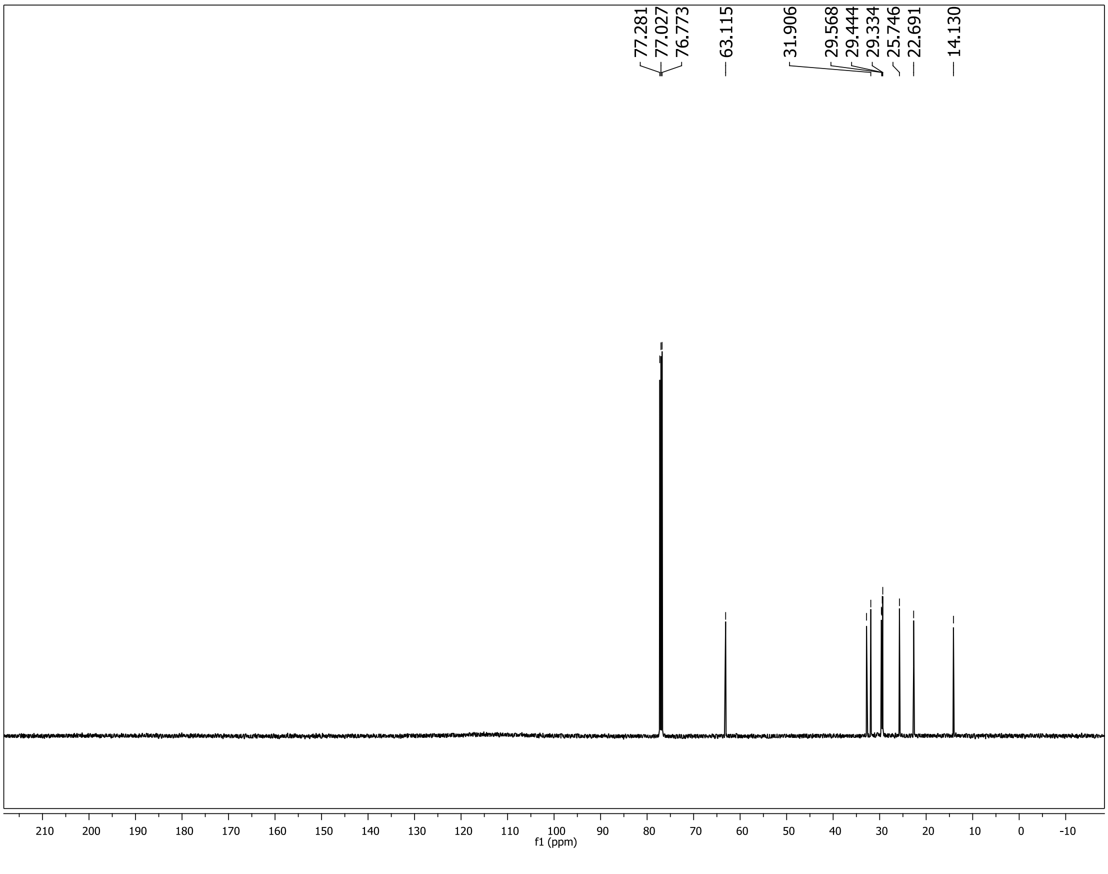
77.281
77.027
76.773
63.115
31.906
29.568
29.444
29.334
25.746
22.691
14.130
210 200 190 180 170 160 150 140 130 120 110 100 90 80 70 60 50 40 30 20 10 0 -10
f1 (ppm)

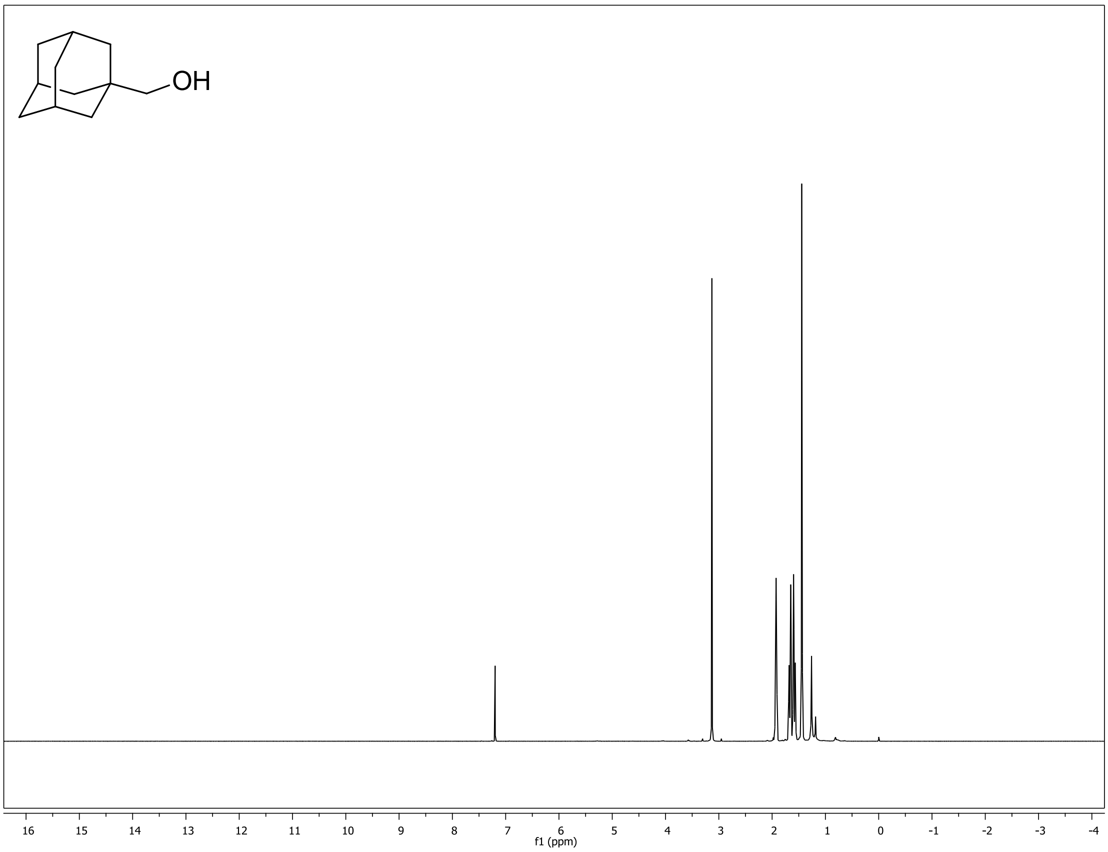
OH
16
15
14
13
12
11
10
9
8
7
6
5
4
3
2
1
0
-1
-2
-3
-4
f1 (ppm)

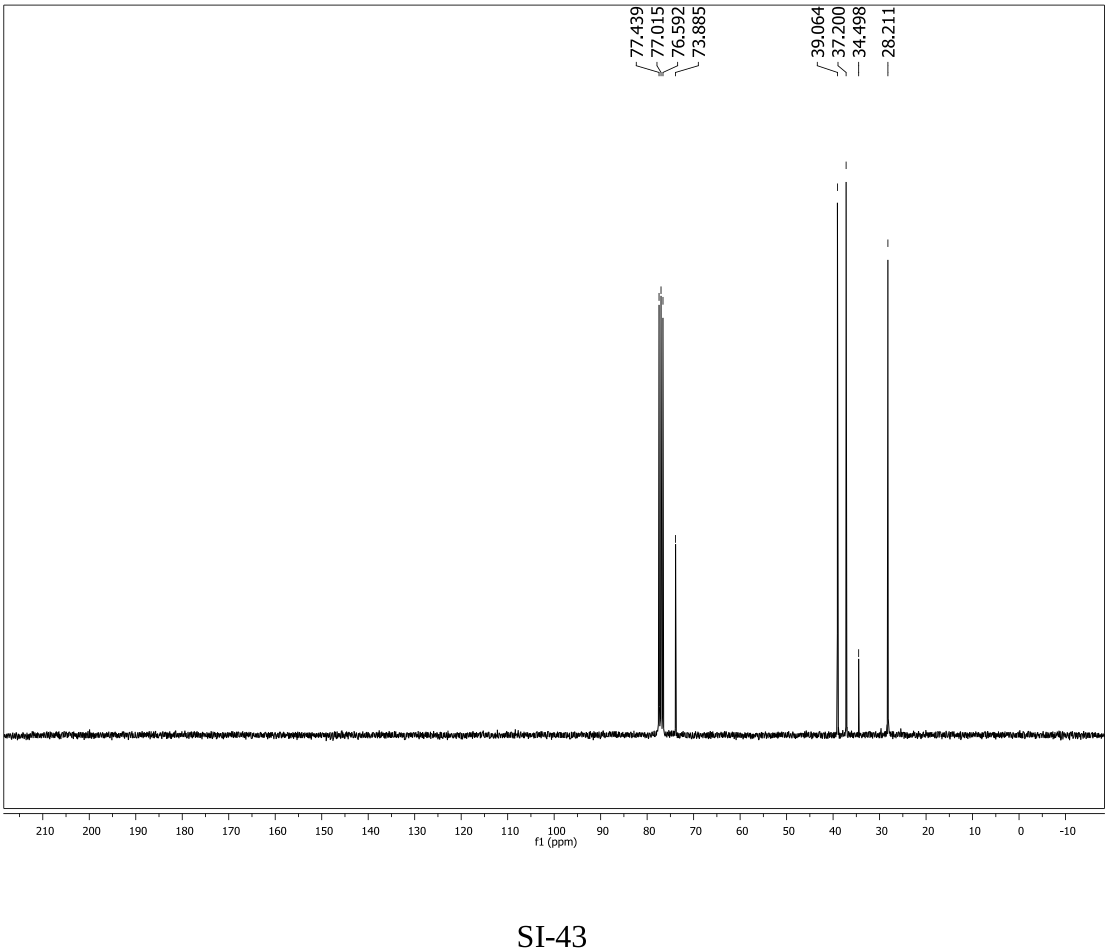
77.439
77.015
76.592
73.885
39.064
37.200
34.498
28.211
210
200
190
180
170
160
150
140
130
120
110
100
90
80
70
60
50
40
30
20
10
0
-10
f1 (ppm)

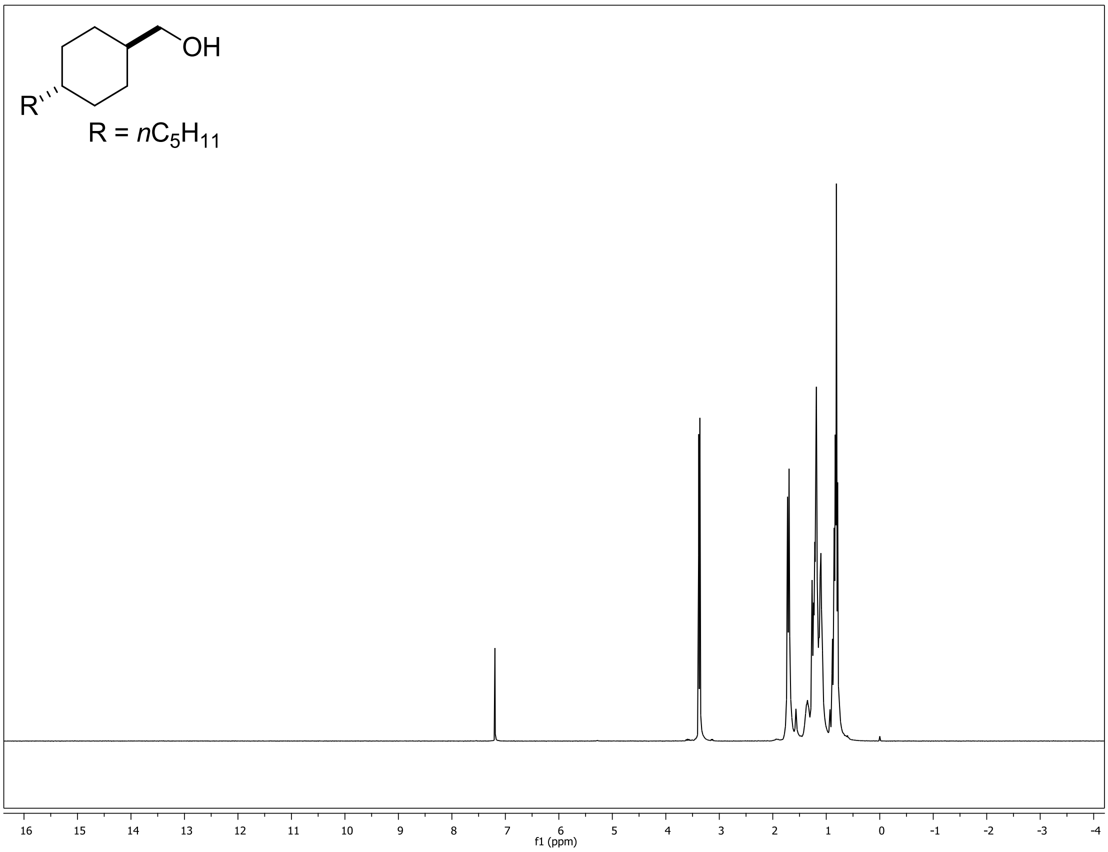
OH
R
R = nC$_5$H$_{11}$
f1 (ppm)

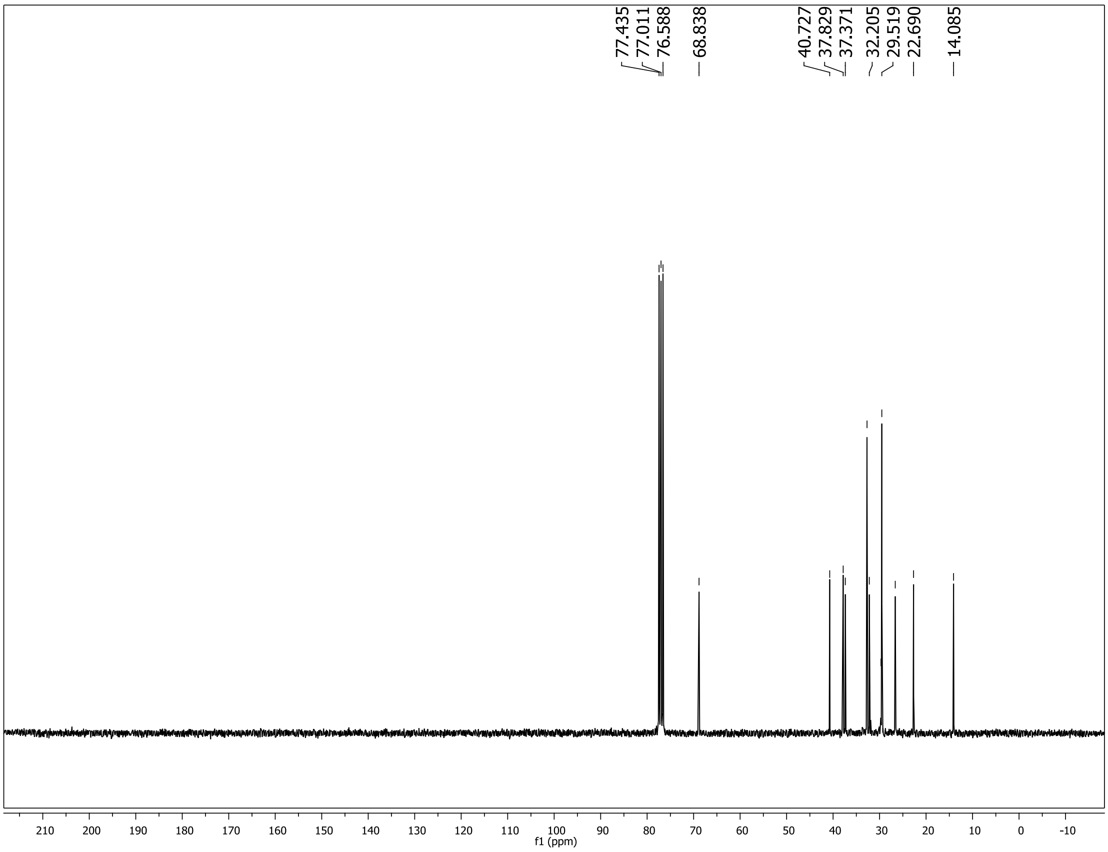
77.435
77.011
76.588
68.838
40.727
37.829
37.371
32.205
29.519
22.690
14.085
f1 (ppm)

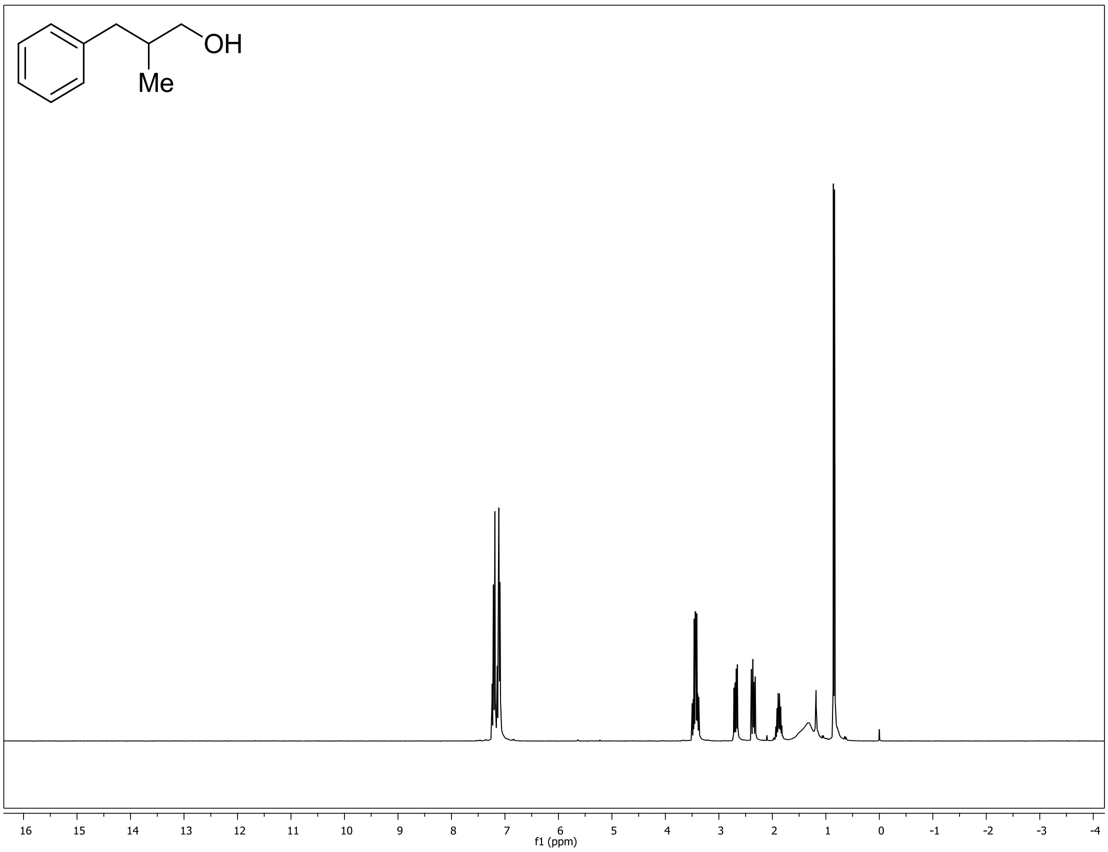
OH
Me
16 15 14 13 12 11 10 9 8 7 6 5 4 3 2 1 0 -1 -2 -3 -4
f1 (ppm)

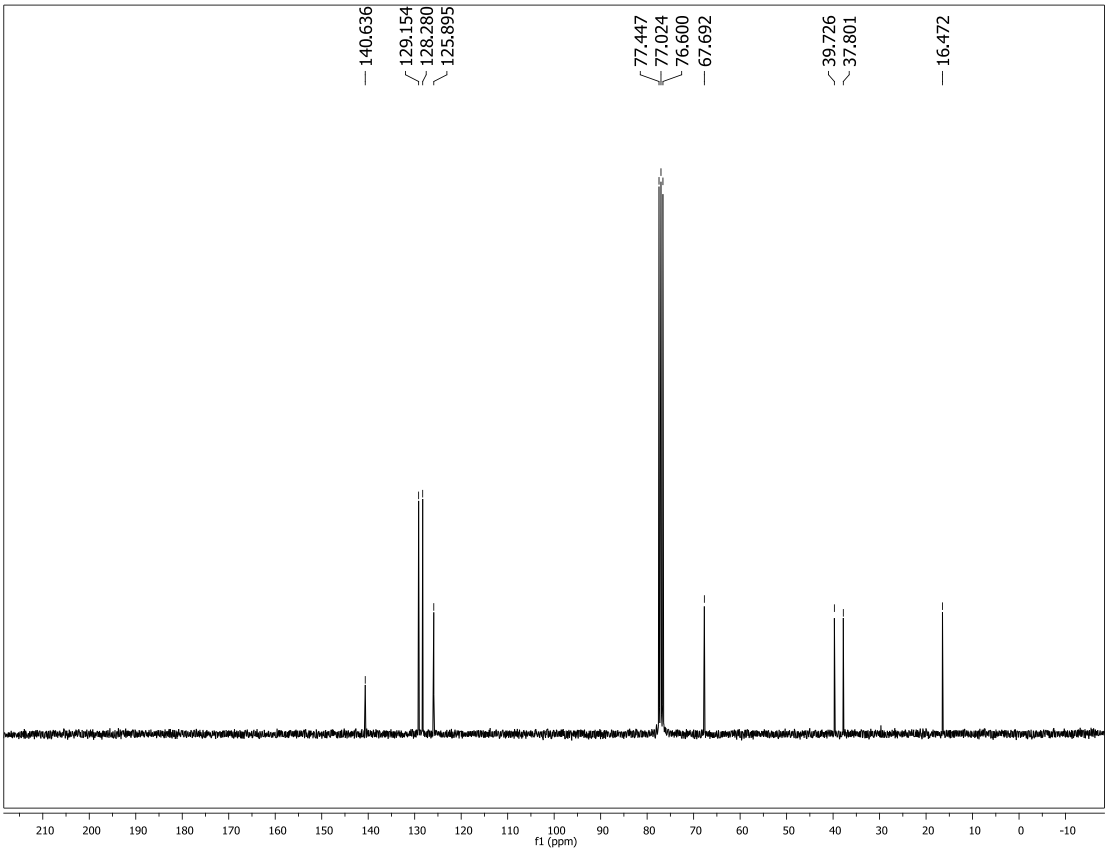
140.636
129.154
128.280
125.895
77.447
77.024
76.600
67.692
39.726
37.801
16.472
210 200 190 180 170 160 150 140 130 120 110 100 90 80 70 60 50 40 30 20 10 0 -10
f1 (ppm)

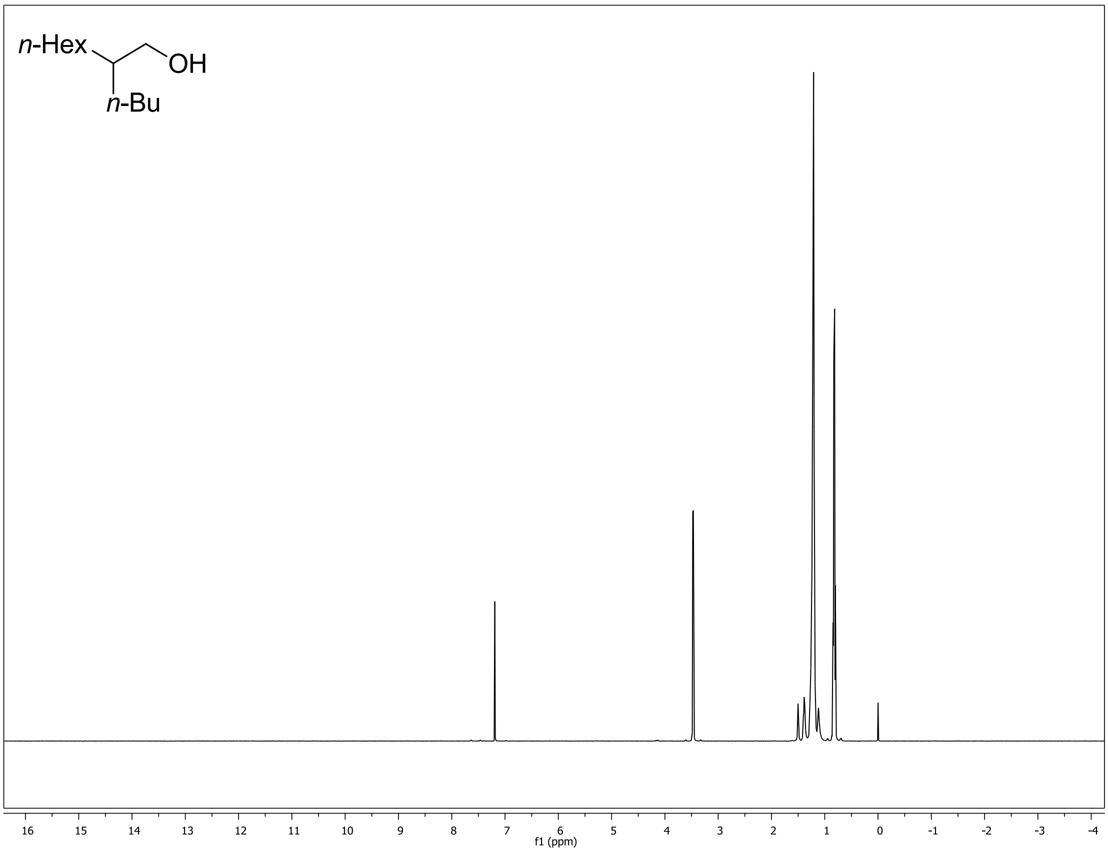
n-Hex
OH
n-Bu
16
15
14
13
12
11
10
9
8
7
6
5
4
3
2
1
0
-1
-2
-3
-4
f1 (ppm)

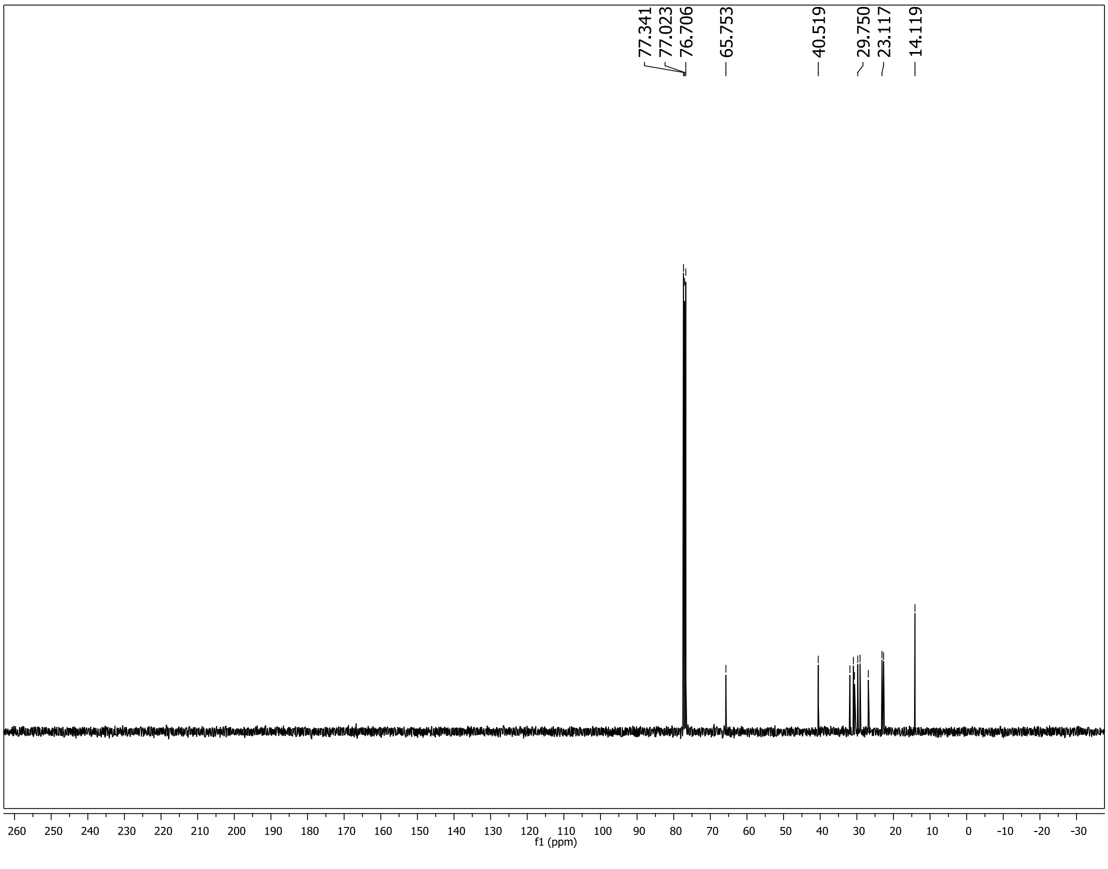
77.341
77.023
76.706
65.753
40.519
29.750
23.117
14.119
260
250
240
230
220
210
200
190
180
170
160
150
140
130
120
110
100
90
80
70
60
50
40
30
20
10
0
-10
-20
-30
f1 (ppm)

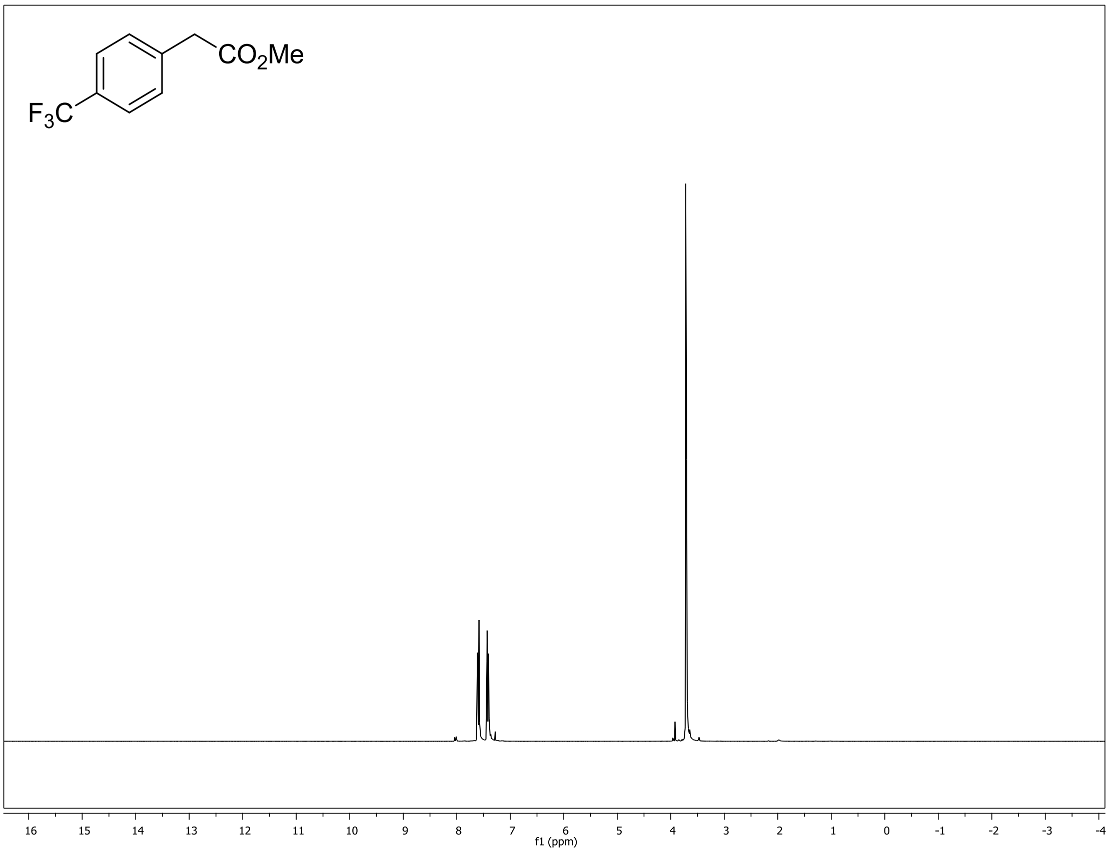
CO_2Me
F_3C
16 15 14 13 12 11 10 9 8 7 6 5 4 3 2 1 0 -1 -2 -3 -4
f1 (ppm)

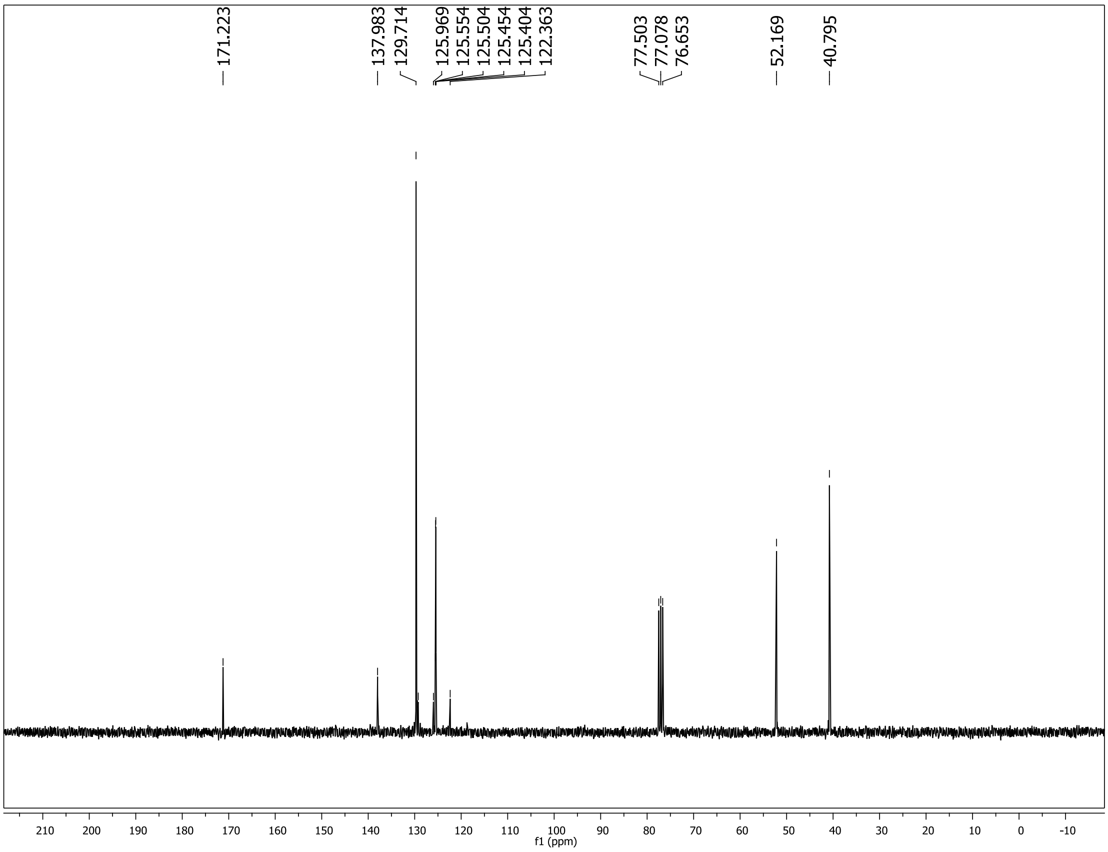
171.223
137.983
129.714
125.969
125.554
125.504
125.454
125.404
122.363
77.503
77.078
76.653
52.169
40.795
210 200 190 180 170 160 150 140 130 120 110 100 90 80 70 60 50 40 30 20 10 0 -10
f1 (ppm)

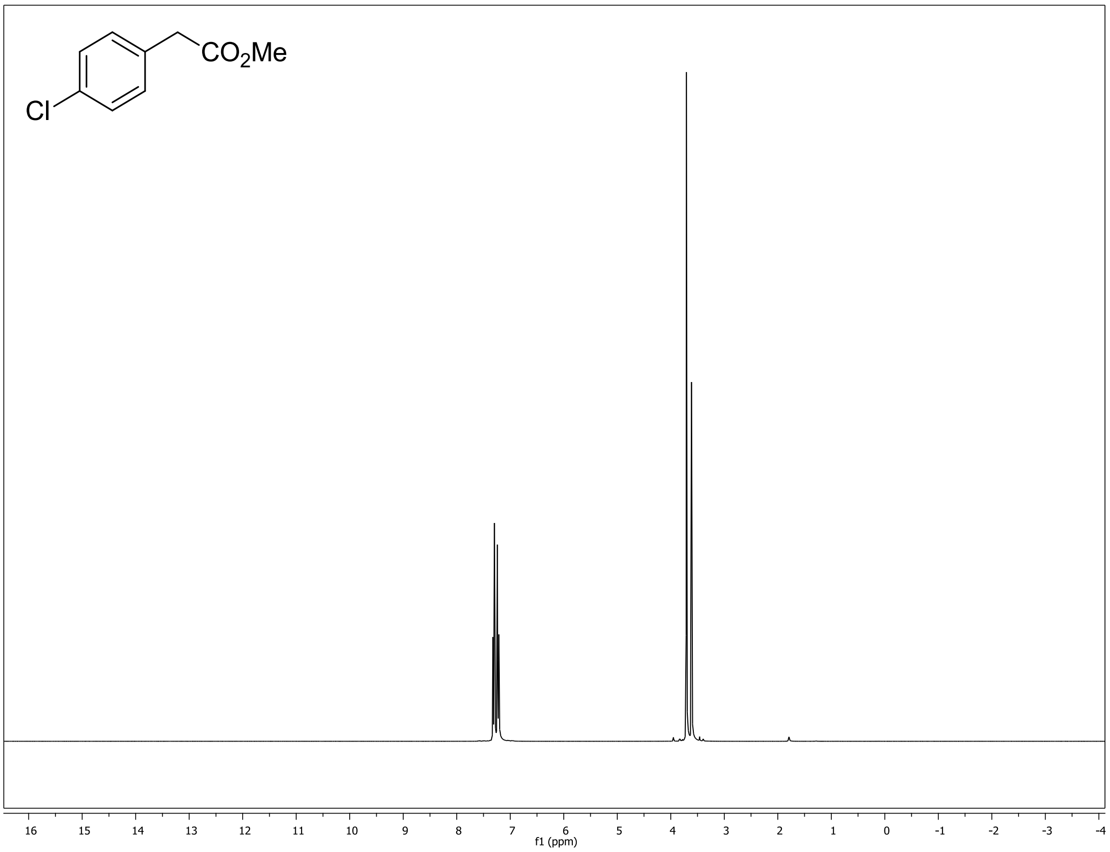
CO2Me
Cl
16 15 14 13 12 11 10 9 8 7 6 5 4 3 2 1 0 -1 -2 -3 -4
f1 (ppm)

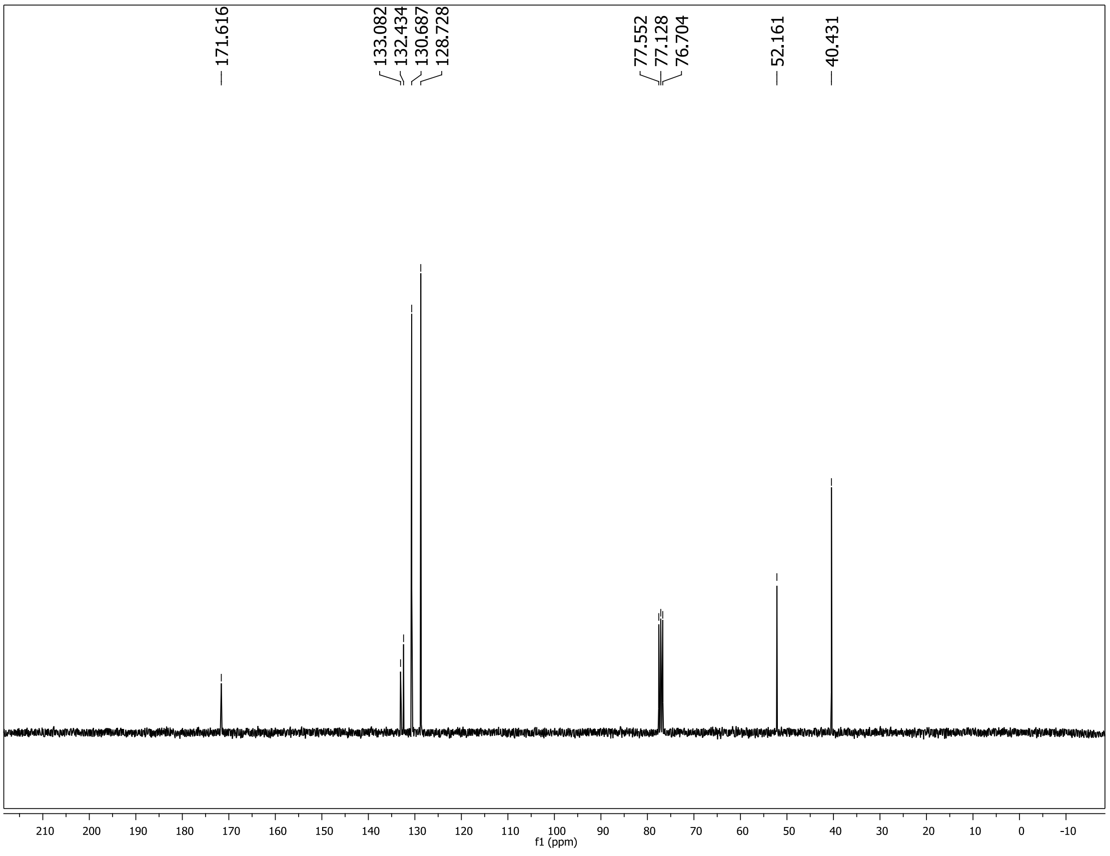
171.616
133.082
132.434
130.687
128.728
77.552
77.128
76.704
52.161
40.431
210 200 190 180 170 160 150 140 130 120 110 100 90 80 70 60 50 40 30 20 10 0 -10
f1 (ppm)

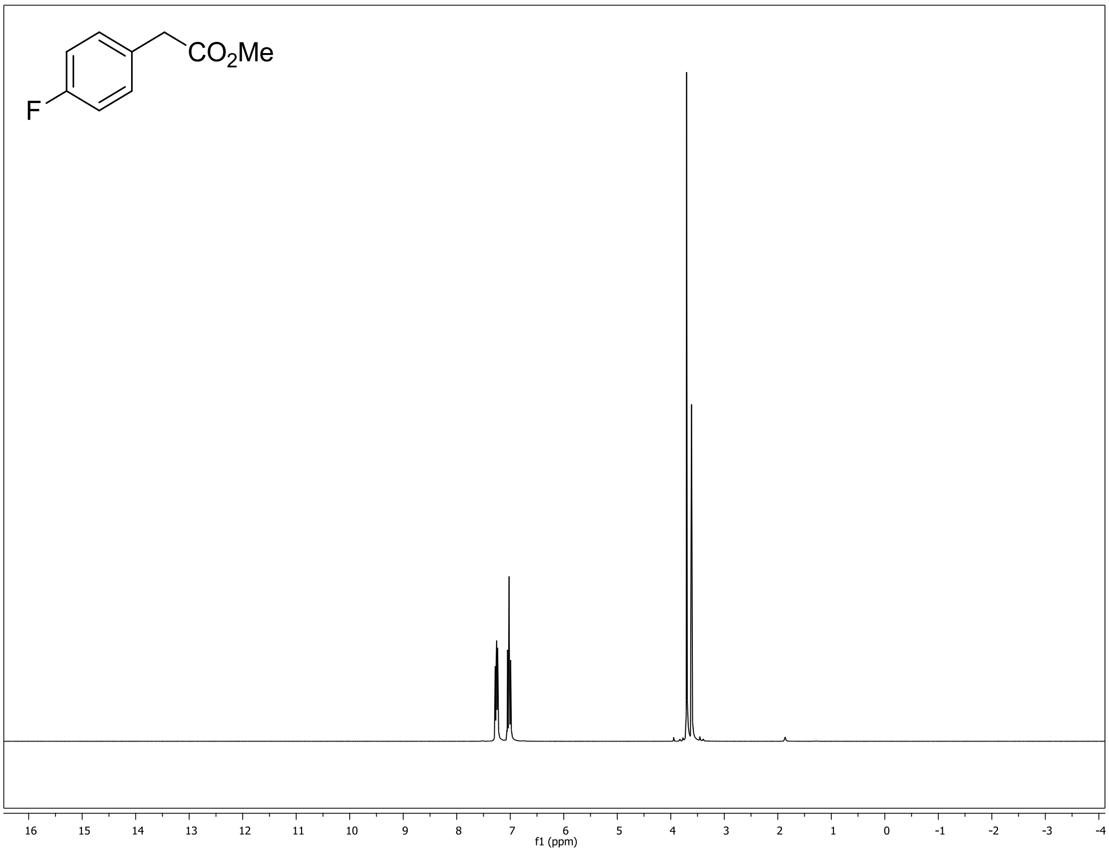
CO2Me
F
16
15
14
13
12
11
10
9
8
7
6
5
4
3
2
1
0
-1
-2
-3
-4
f1 (ppm)

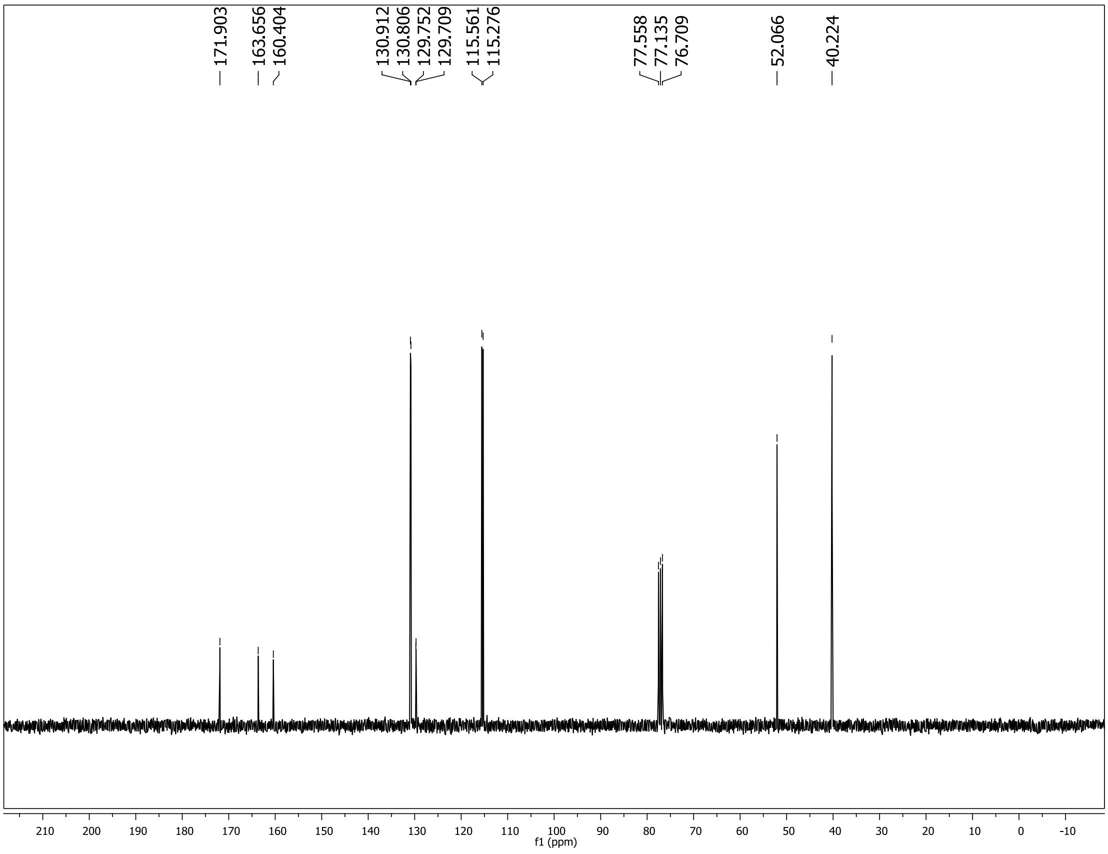
171.903
163.656
160.404
130.912
130.806
129.752
129.709
115.561
115.276
77.558
77.135
76.709
52.066
40.224
210
200
190
180
170
160
150
140
130
120
110
100
90
80
70
60
50
40
30
20
10
0
-10
f1 (ppm)

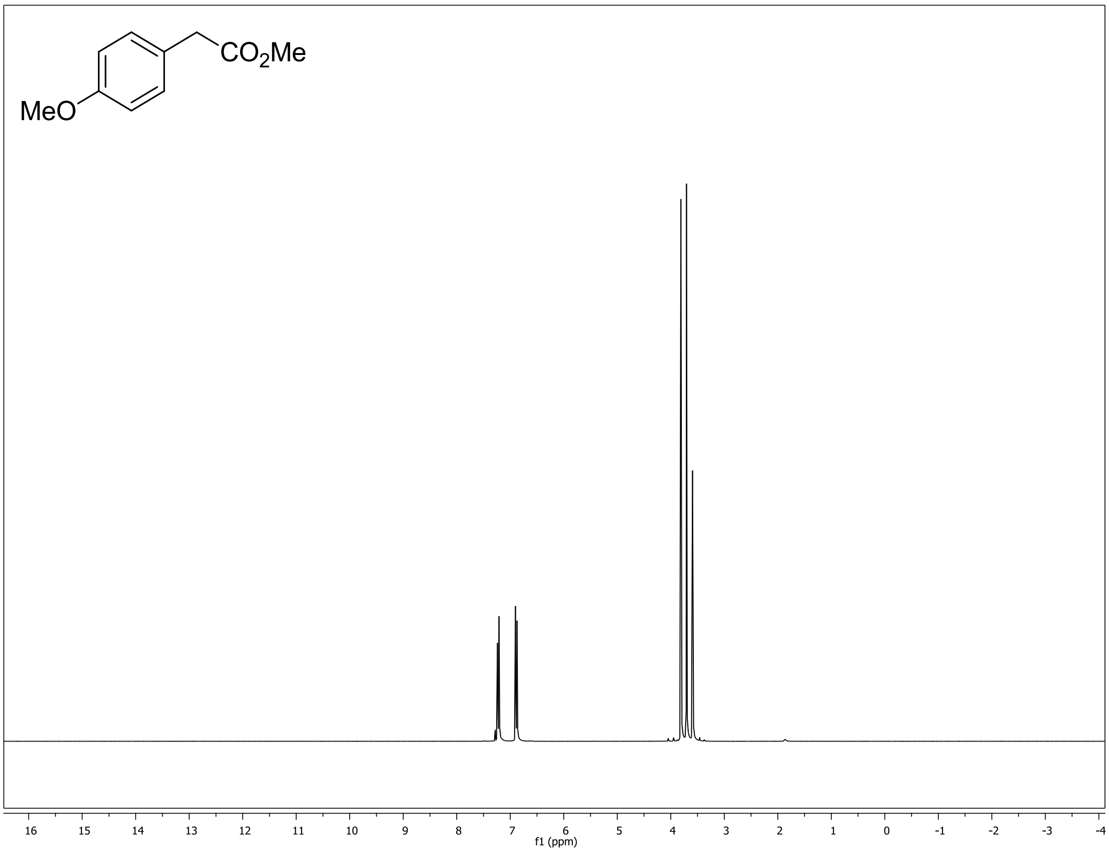
CO2Me
MeO
f1 (ppm)

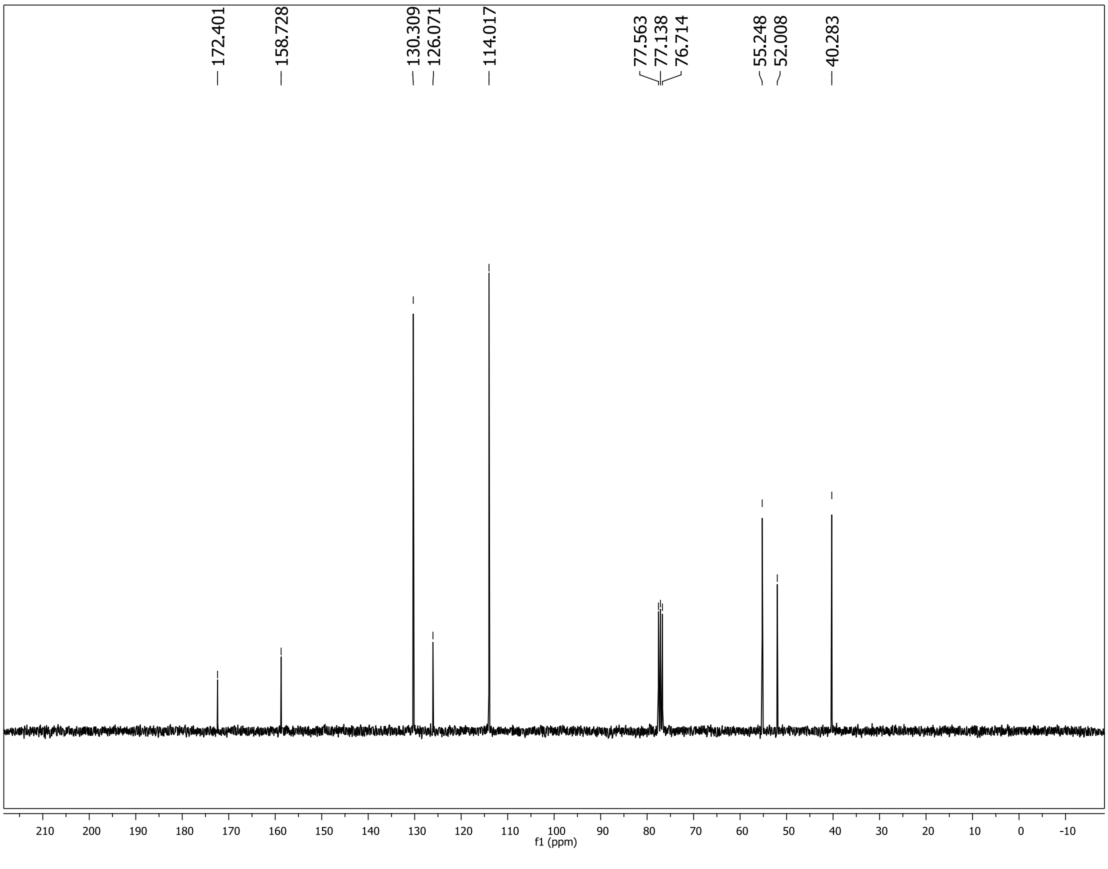
172.401
158.728
130.309
126.071
114.017
77.563
77.138
76.714
55.248
52.008
40.283
f1 (ppm)

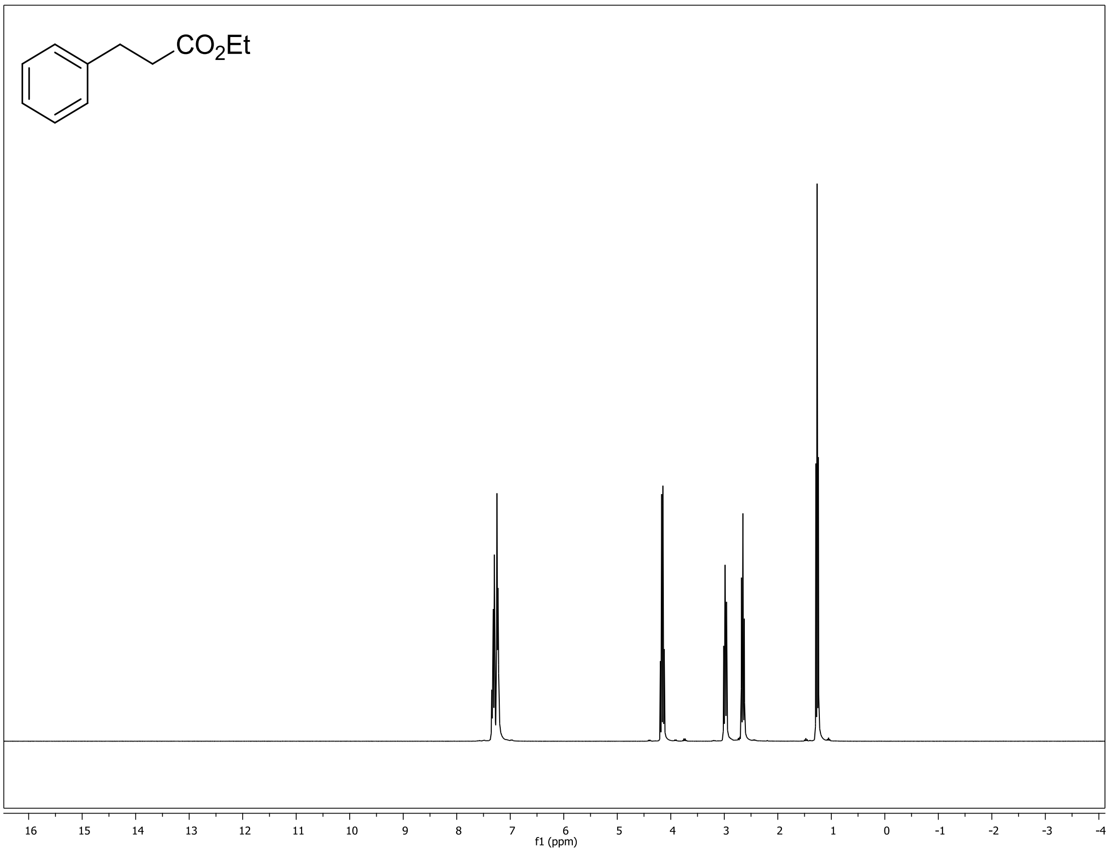
CO2Et
16 15 14 13 12 11 10 9 8 7 6 5 4 3 2 1 0 -1 -2 -3 -4
f1 (ppm)

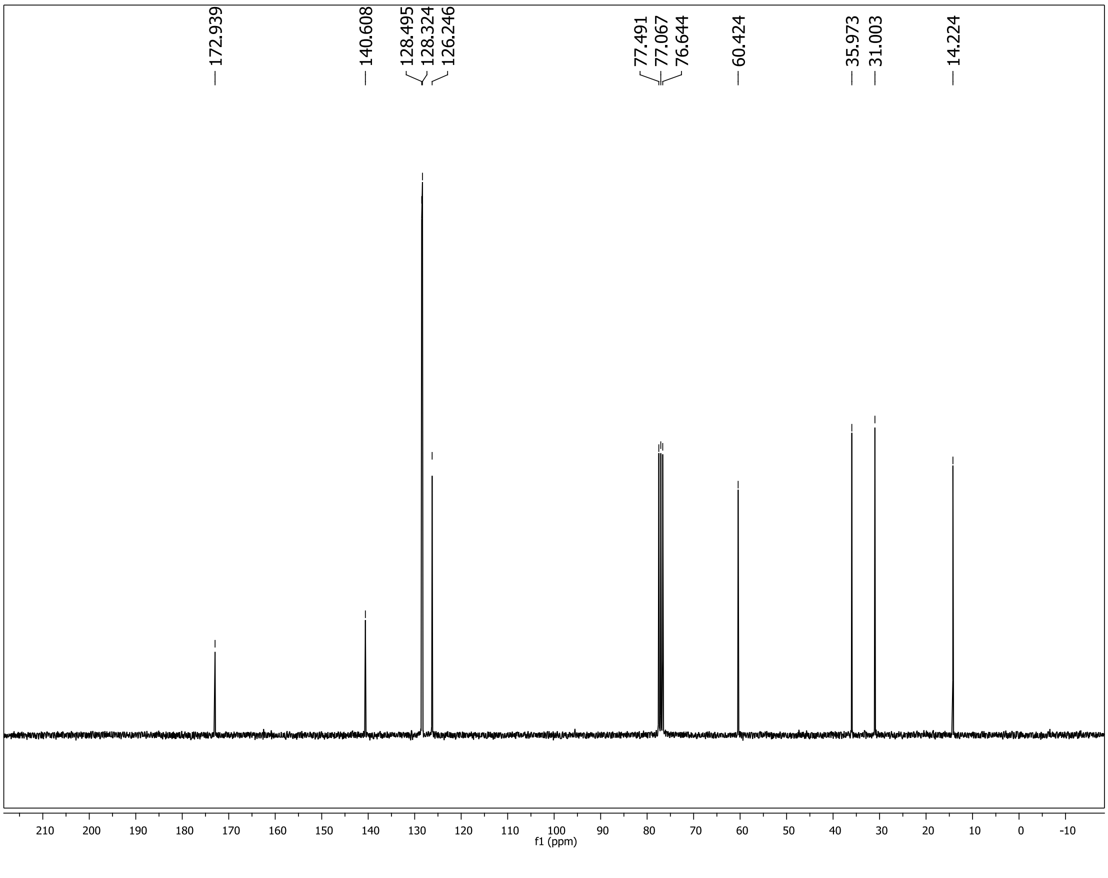
172.939
140.608
128.495
128.324
126.246
77.491
77.067
76.644
60.424
35.973
31.003
14.224
210 200 190 180 170 160 150 140 130 120 110 100 90 80 70 60 50 40 30 20 10 0 -10
f1 (ppm)

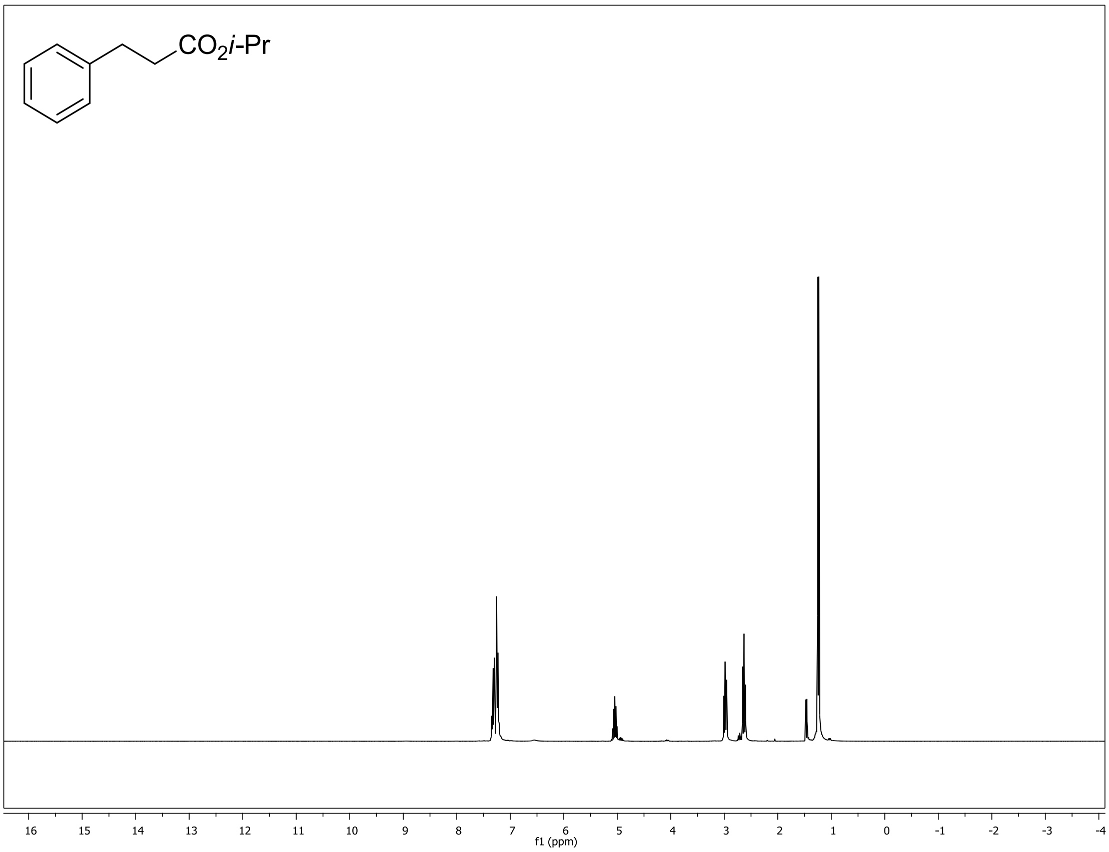
CO2i-Pr
16 15 14 13 12 11 10 9 8 7 6 5 4 3 2 1 0 -1 -2 -3 -4
f1 (ppm)

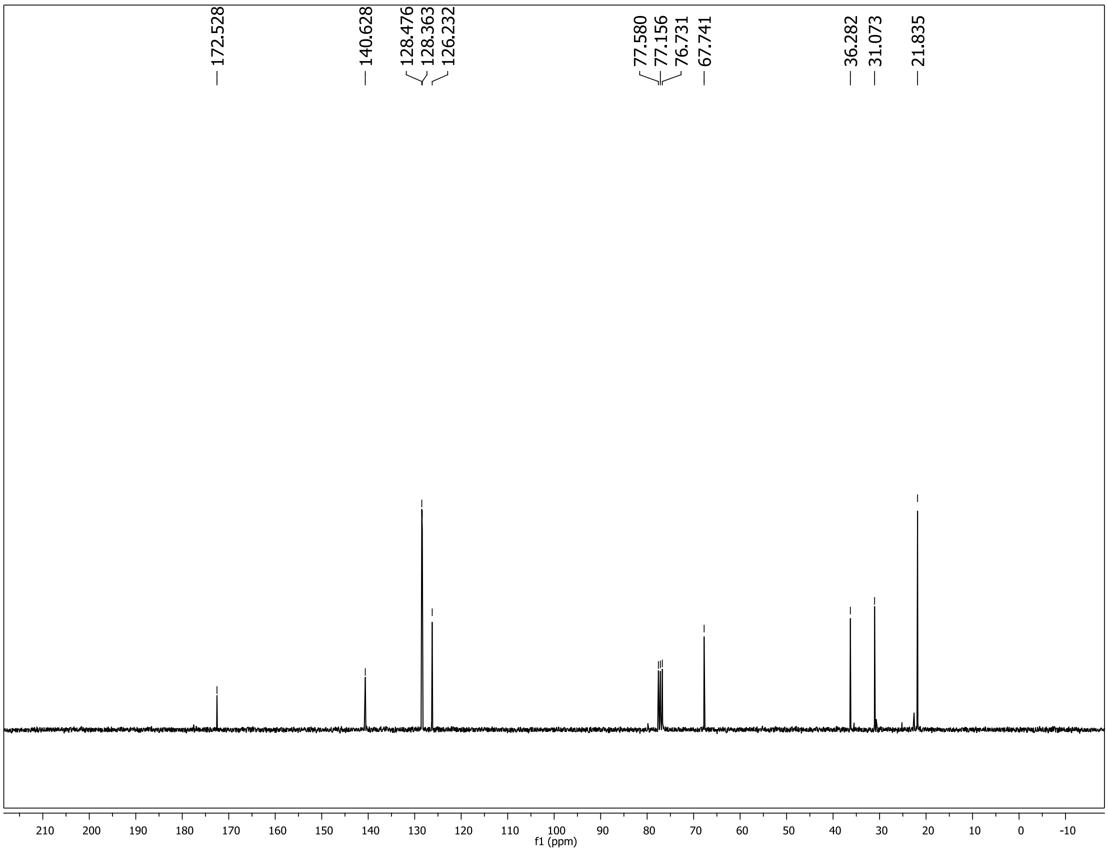
172.528
140.628
128.476
128.363
126.232
77.580
77.156
76.731
67.741
36.282
31.073
21.835
210 200 190 180 170 160 150 140 130 120 110 100 90 80 70 60 50 40 30 20 10 0 -10
f1 (ppm)

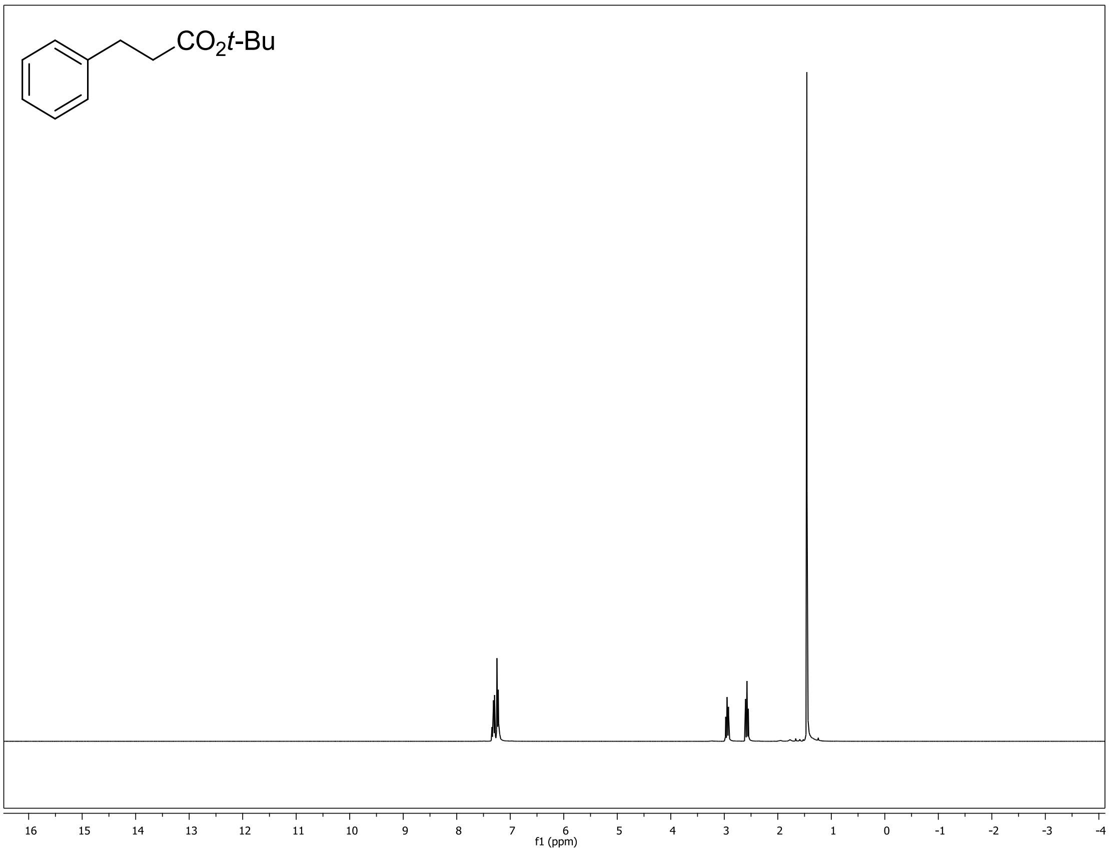

CO2t-Bu
f1 (ppm)

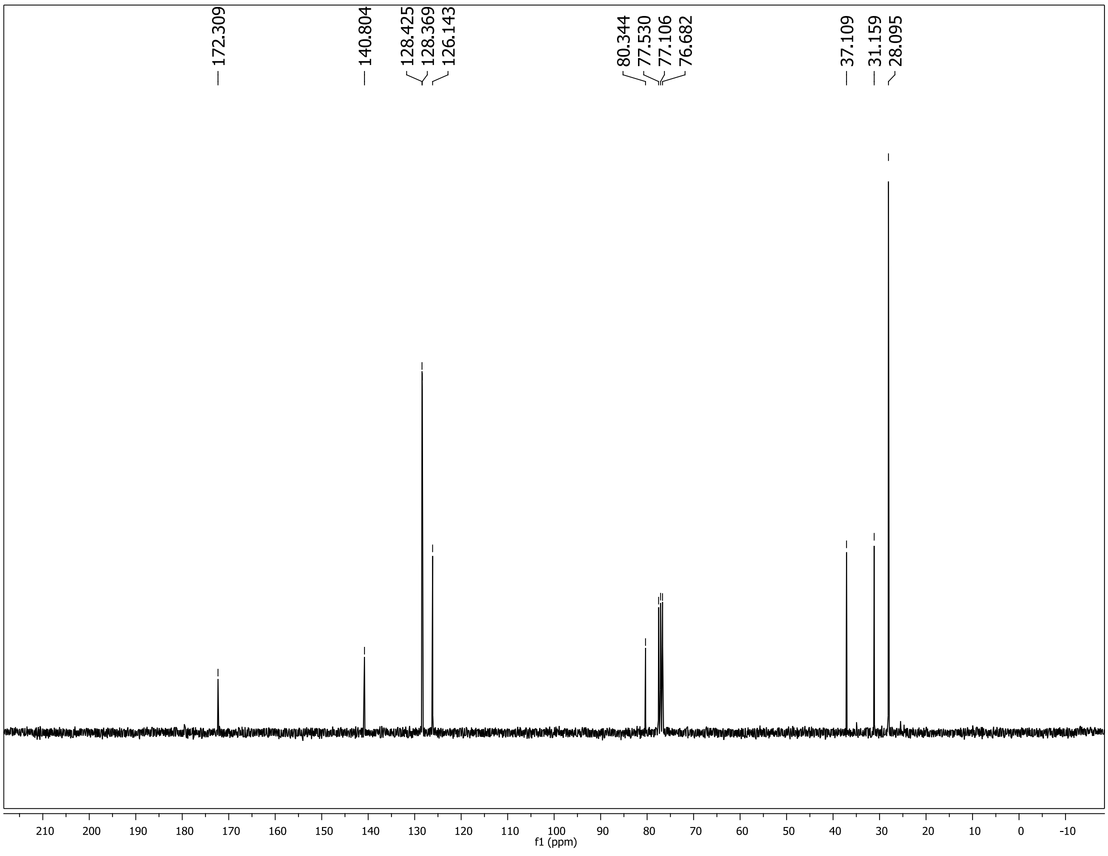

172.309
140.804
128.425
128.369
126.143
80.344
77.530
77.106
76.682
37.109
31.159
28.095
f1 (ppm)

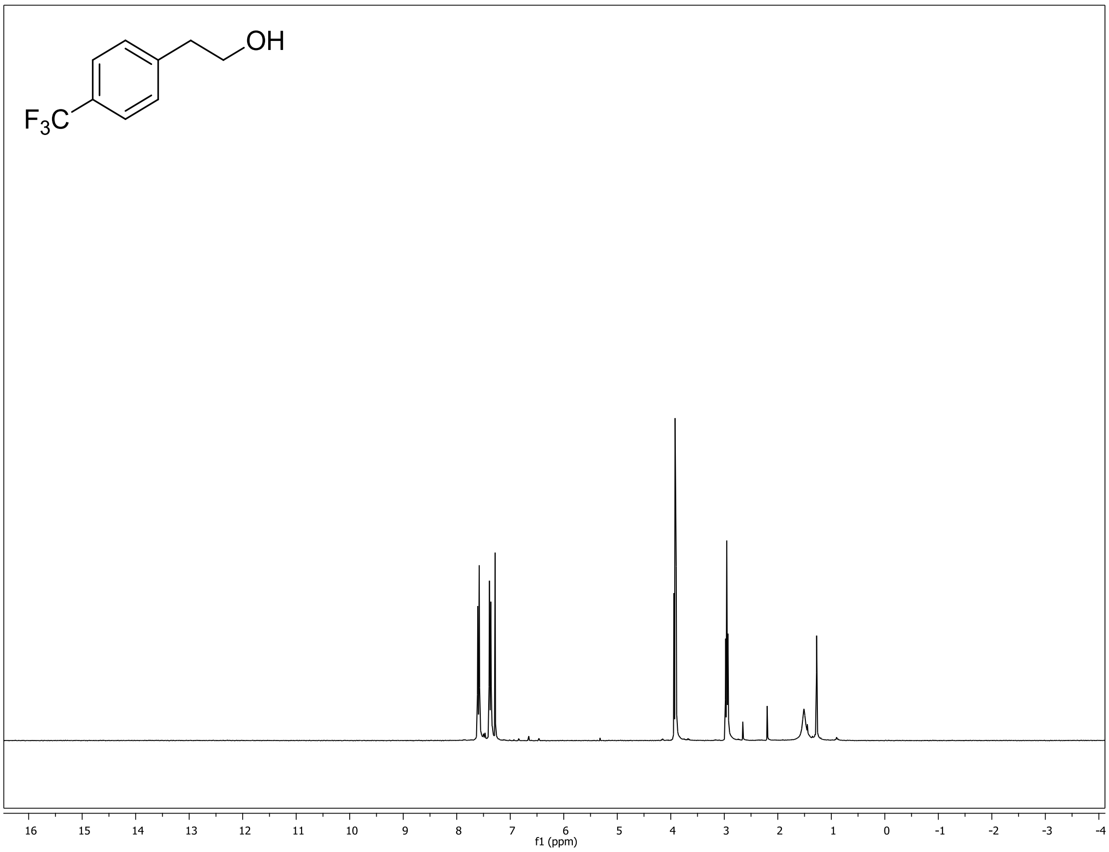
OH
F_3C
16 15 14 13 12 11 10 9 8 7 6 5 4 3 2 1 0 -1 -2 -3 -4
f1 (ppm)

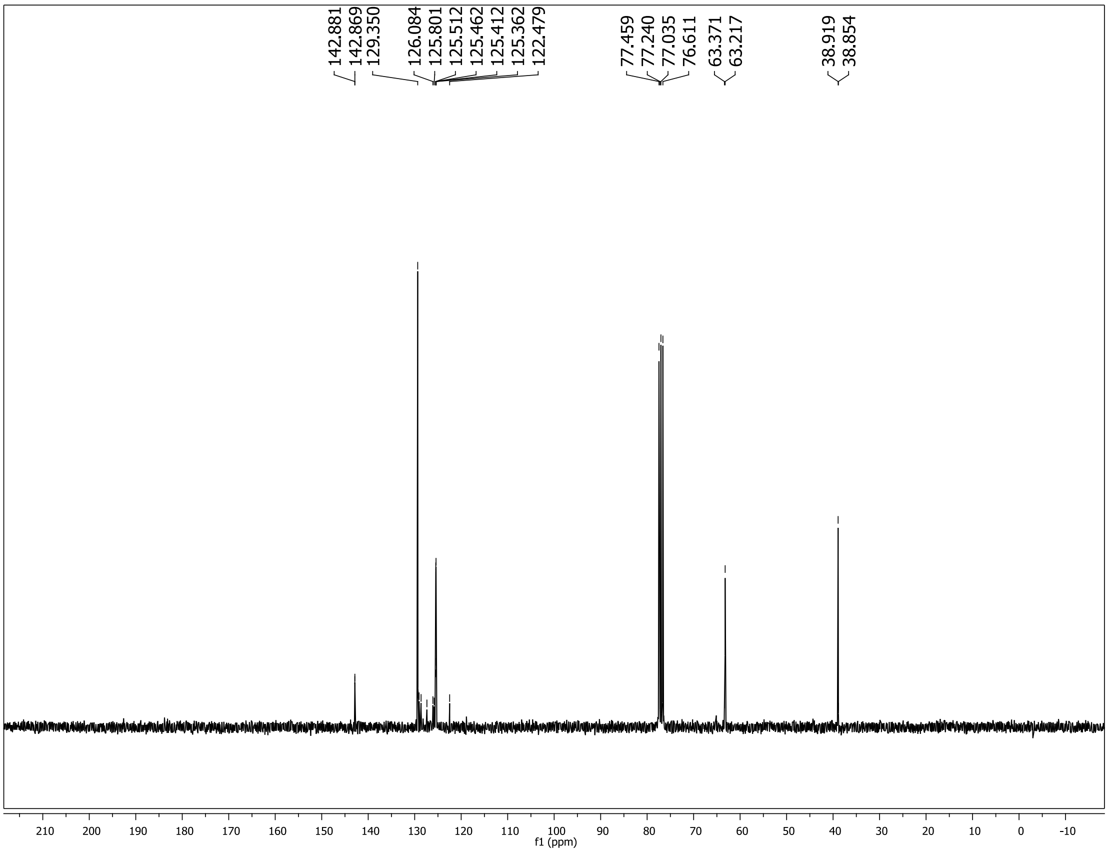
142.881
142.869
129.350
126.084
125.801
125.512
125.462
125.412
125.362
122.479
77.459
77.240
77.035
76.611
63.371
63.217
38.919
38.854
210 200 190 180 170 160 150 140 130 120 110 100 90 80 70 60 50 40 30 20 10 0 -10
f1 (ppm)

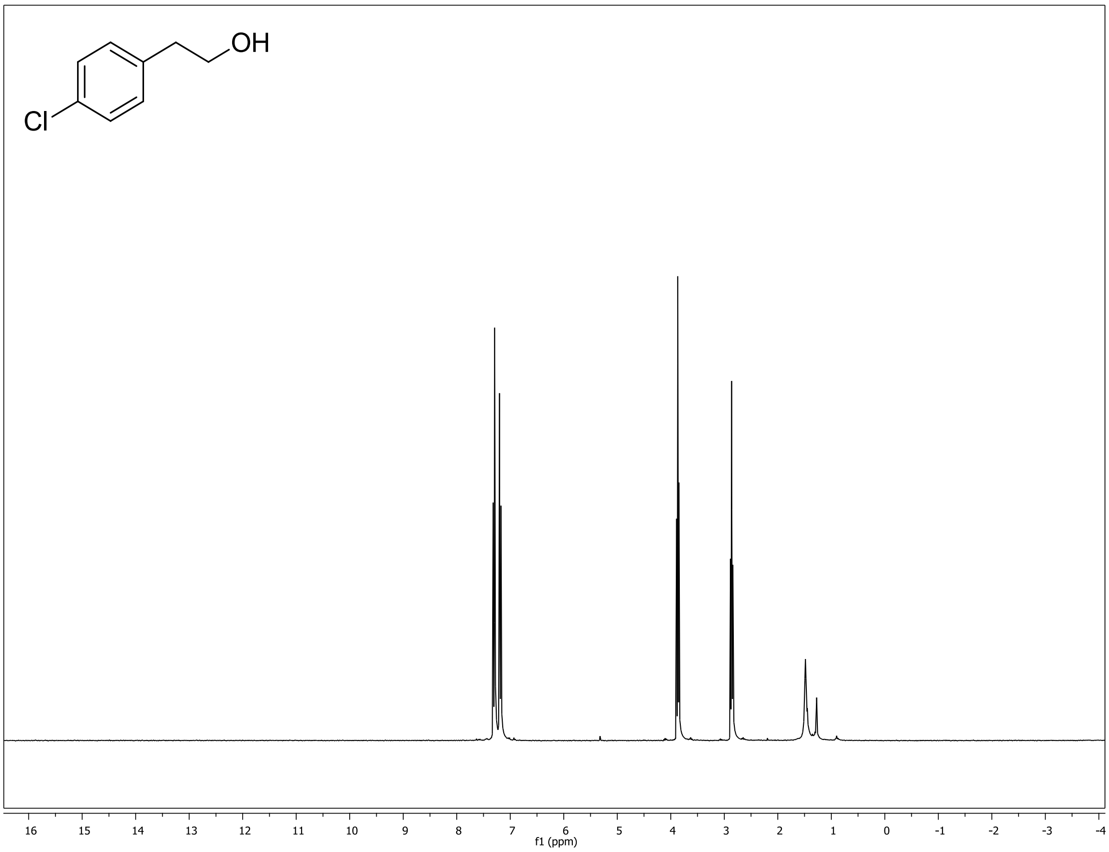
OH
Cl
f1 (ppm)

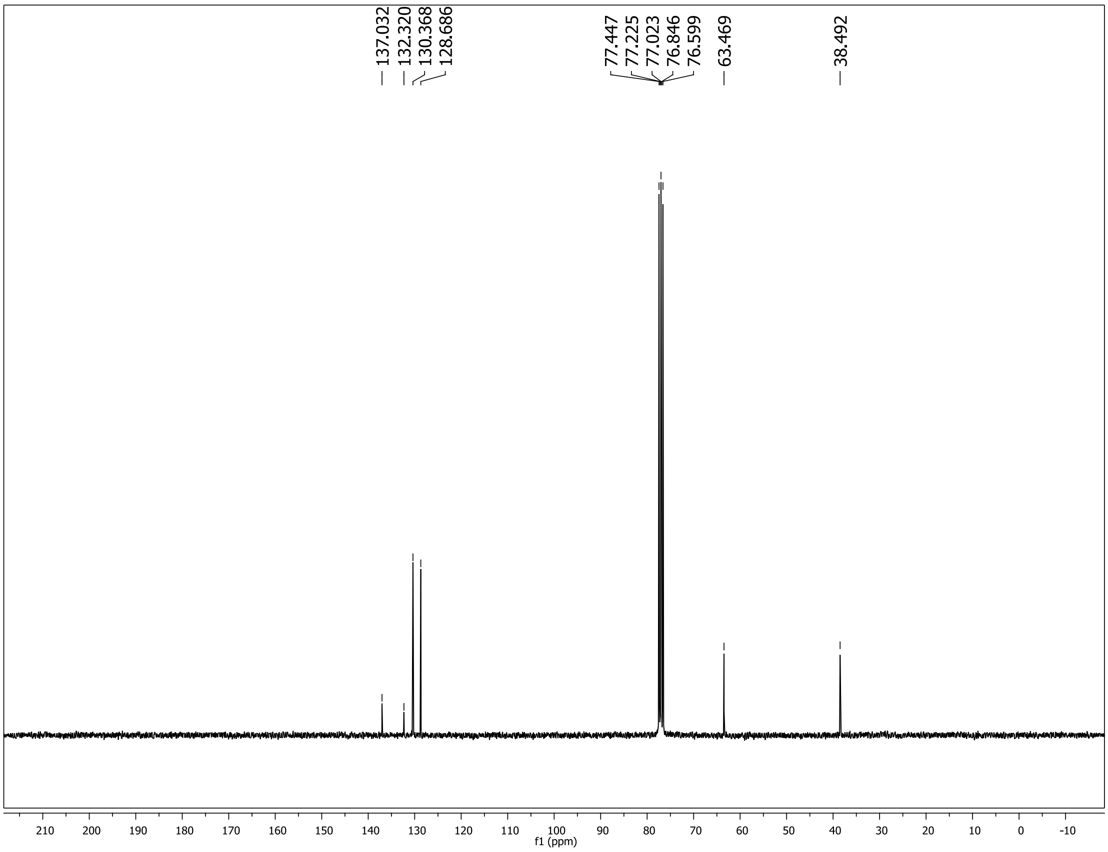
137.032
132.320
130.368
128.686
77.447
77.225
77.023
76.846
76.599
63.469
38.492
f1 (ppm)

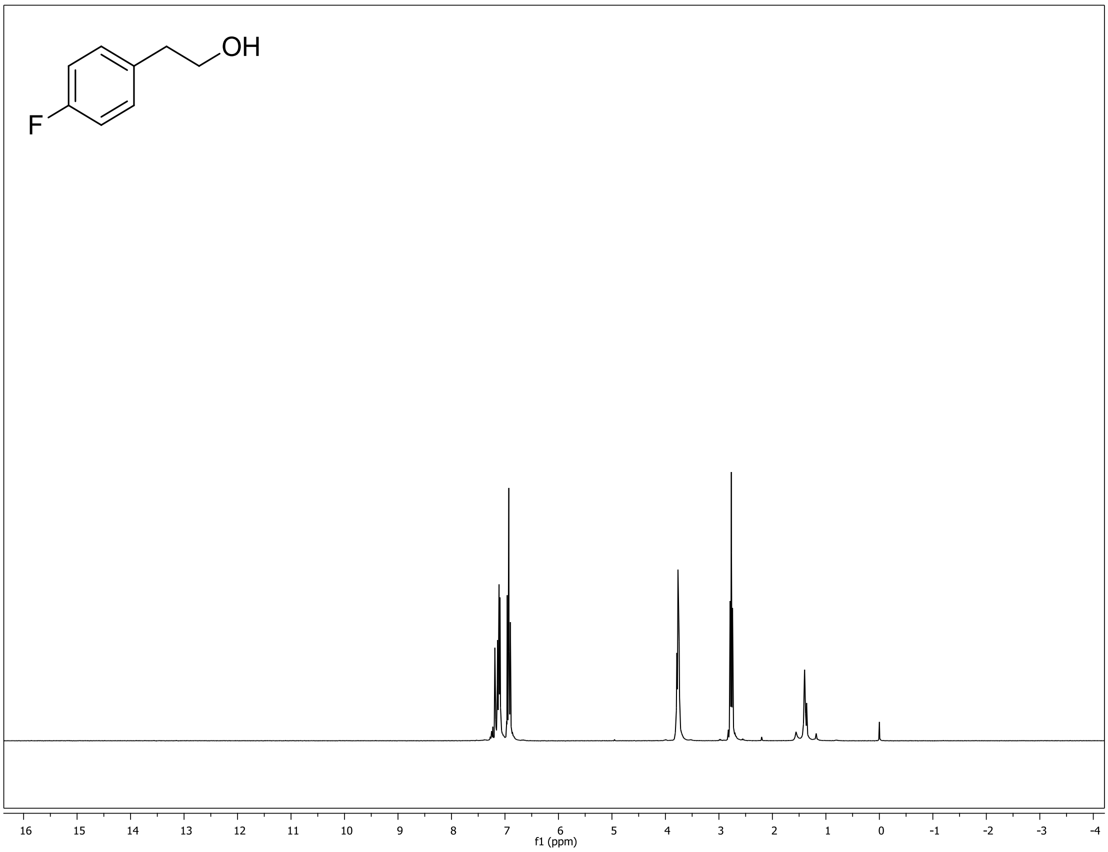
OH
F
f1 (ppm)

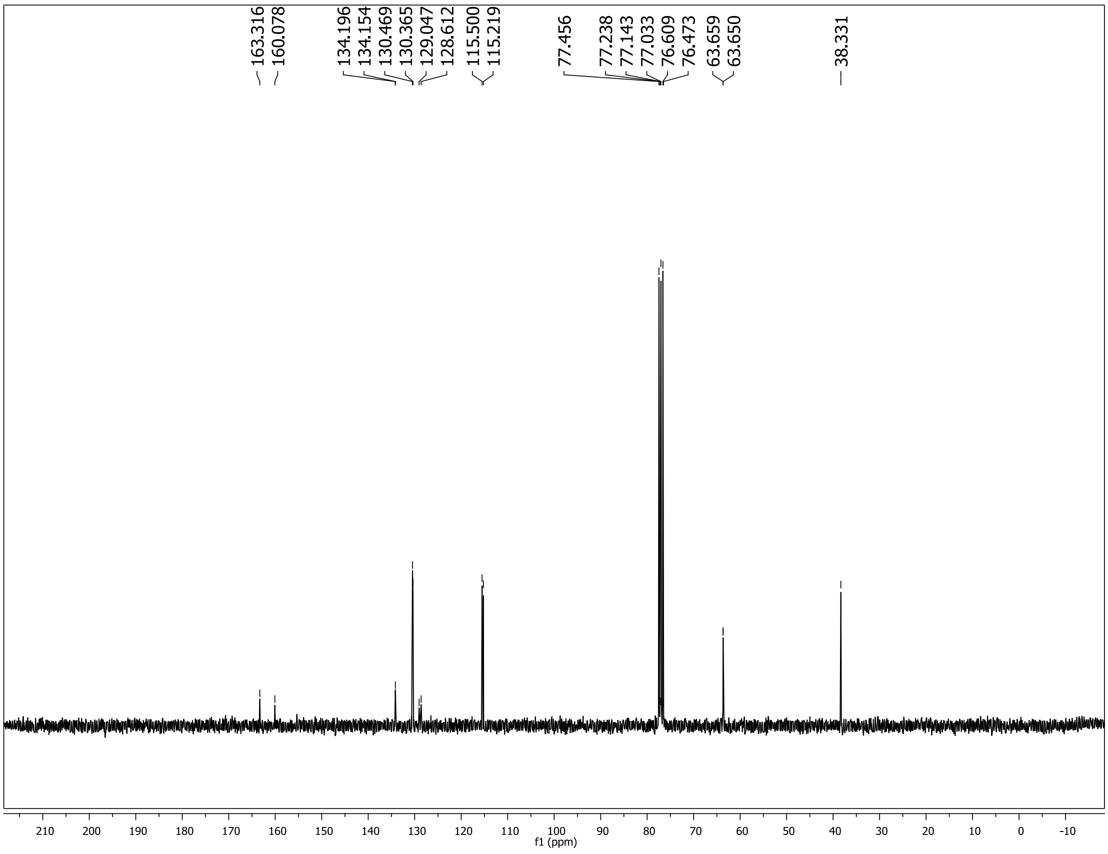
163.316
160.078
134.196
134.154
130.469
130.365
129.047
128.612
115.500
115.219
77.456
77.238
77.143
77.033
76.609
76.473
63.659
63.650
38.331
f1 (ppm)

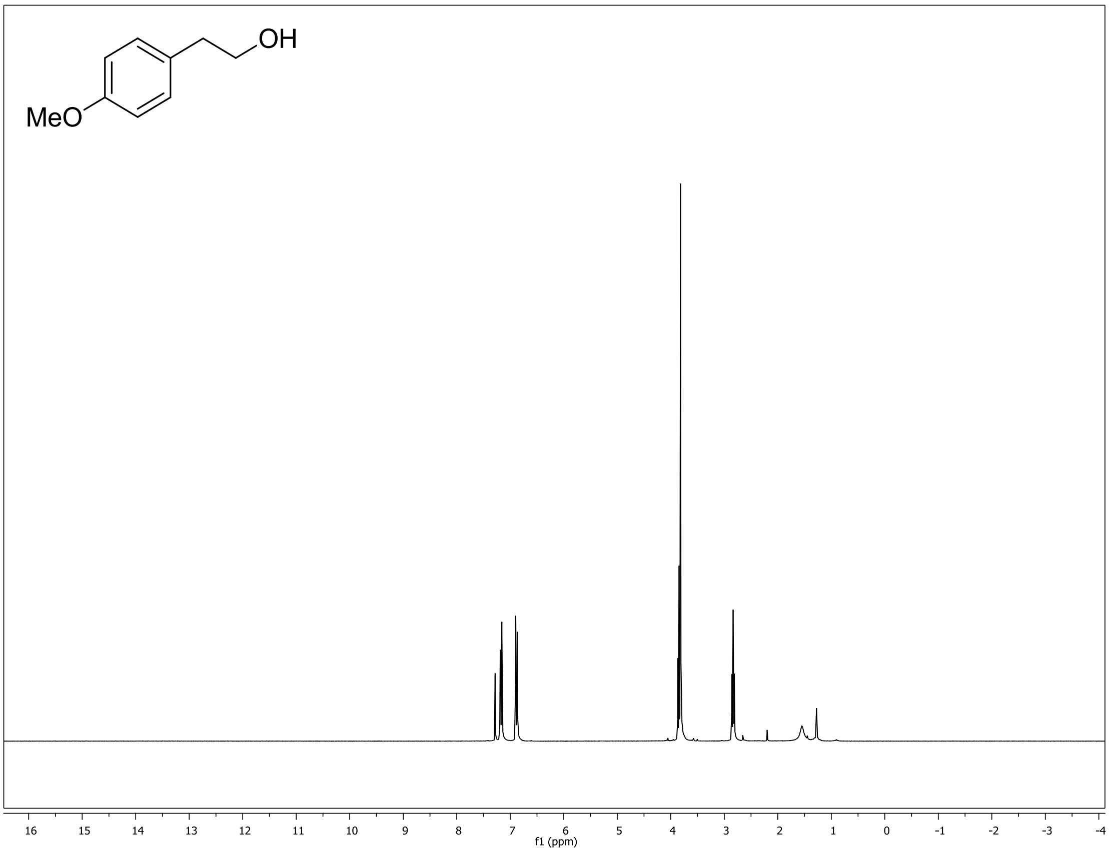
OH
MeO
16 15 14 13 12 11 10 9 8 7 6 5 4 3 2 1 0 -1 -2 -3 -4
f1 (ppm)

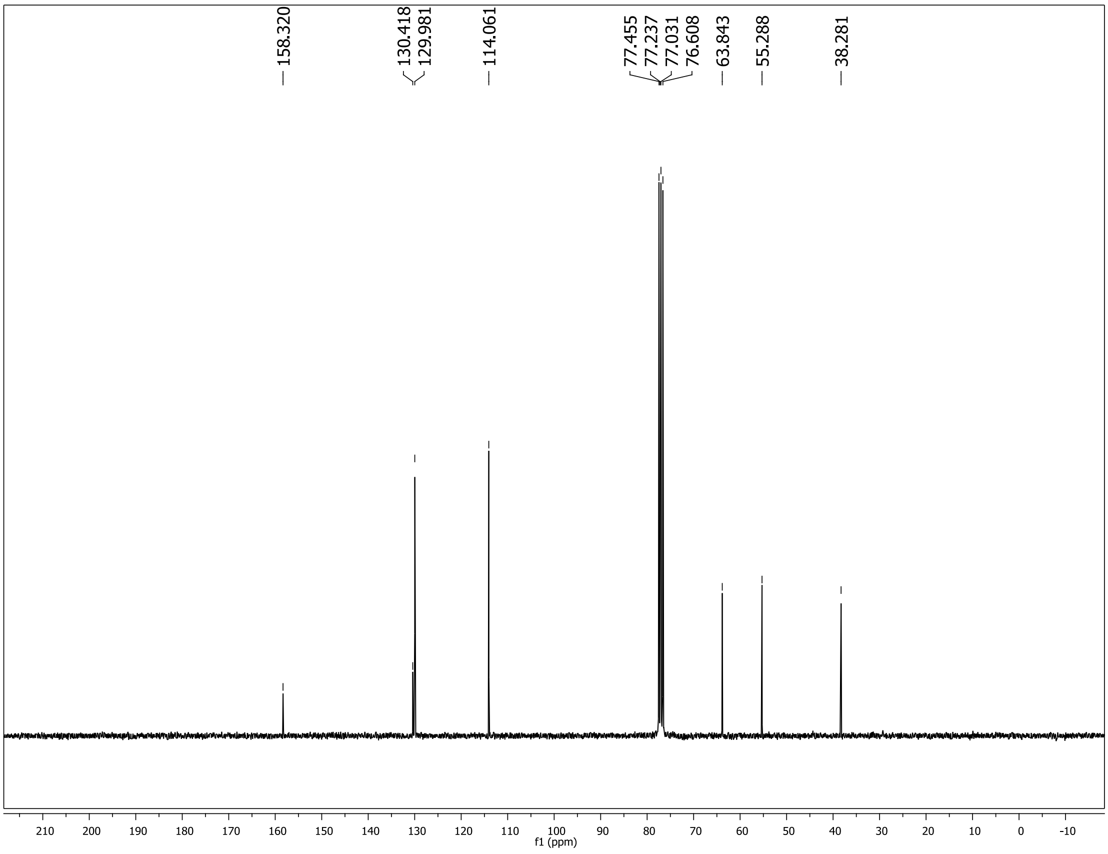
158.320
130.418
129.981
114.061
77.455
77.237
77.031
76.608
63.843
55.288
38.281
210 200 190 180 170 160 150 140 130 120 110 100 90 80 70 60 50 40 30 20 10 0 -10
f1 (ppm)

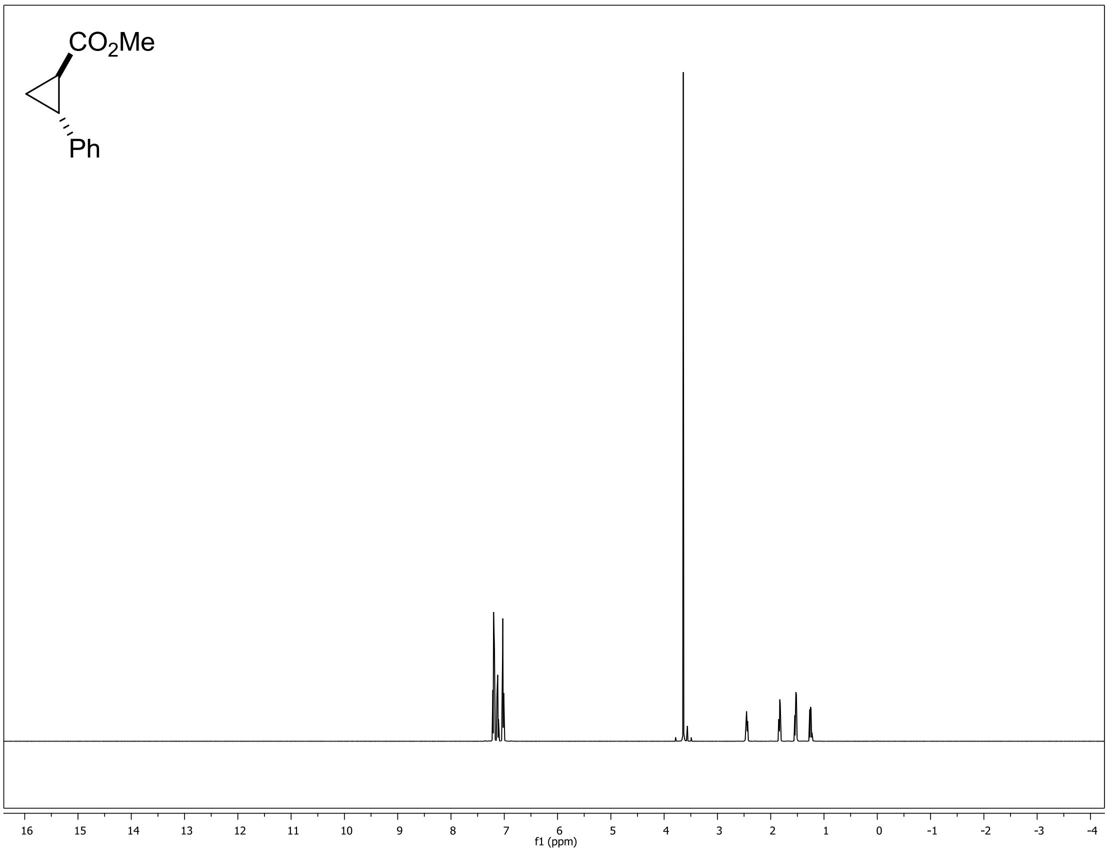
CO2Me
Ph
16 15 14 13 12 11 10 9 8 7 6 5 4 3 2 1 0 -1 -2 -3 -4
f1 (ppm)

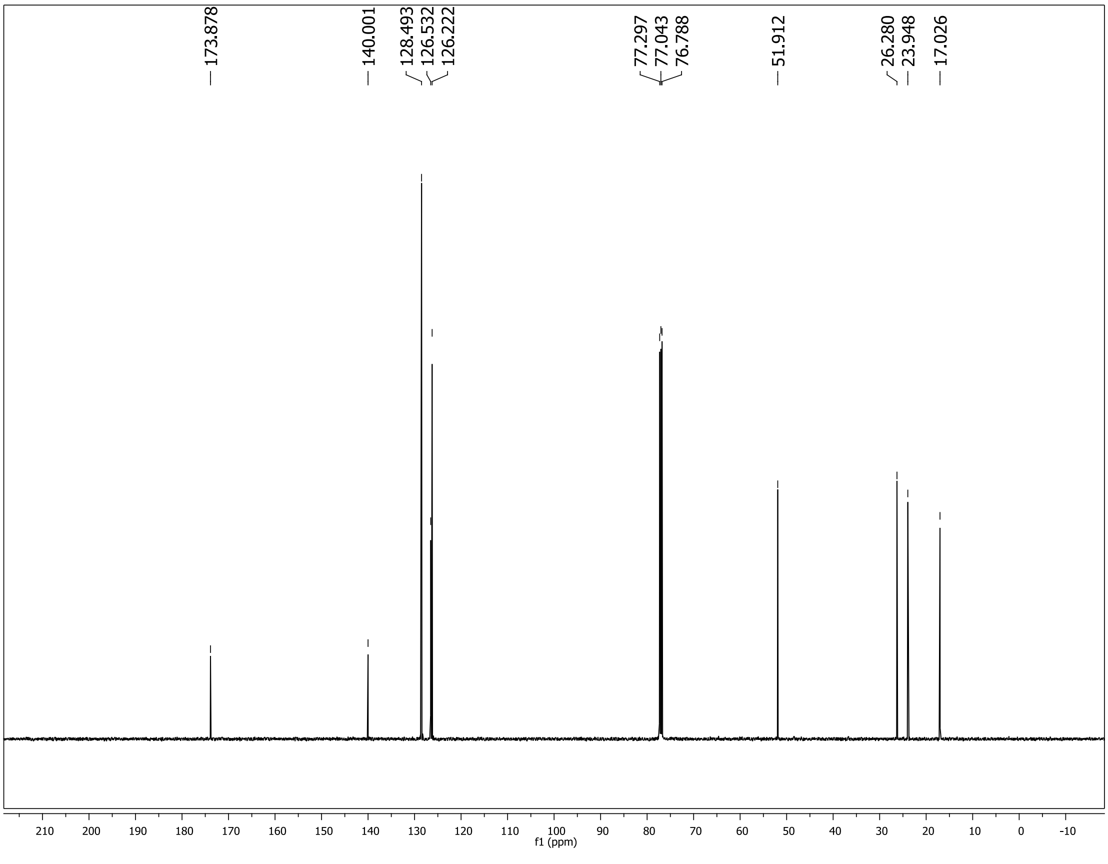
173.878
140.001
128.493
126.532
126.222
77.297
77.043
76.788
51.912
26.280
23.948
17.026
210 200 190 180 170 160 150 140 130 120 110 100 90 80 70 60 50 40 30 20 10 0 -10
f1 (ppm)

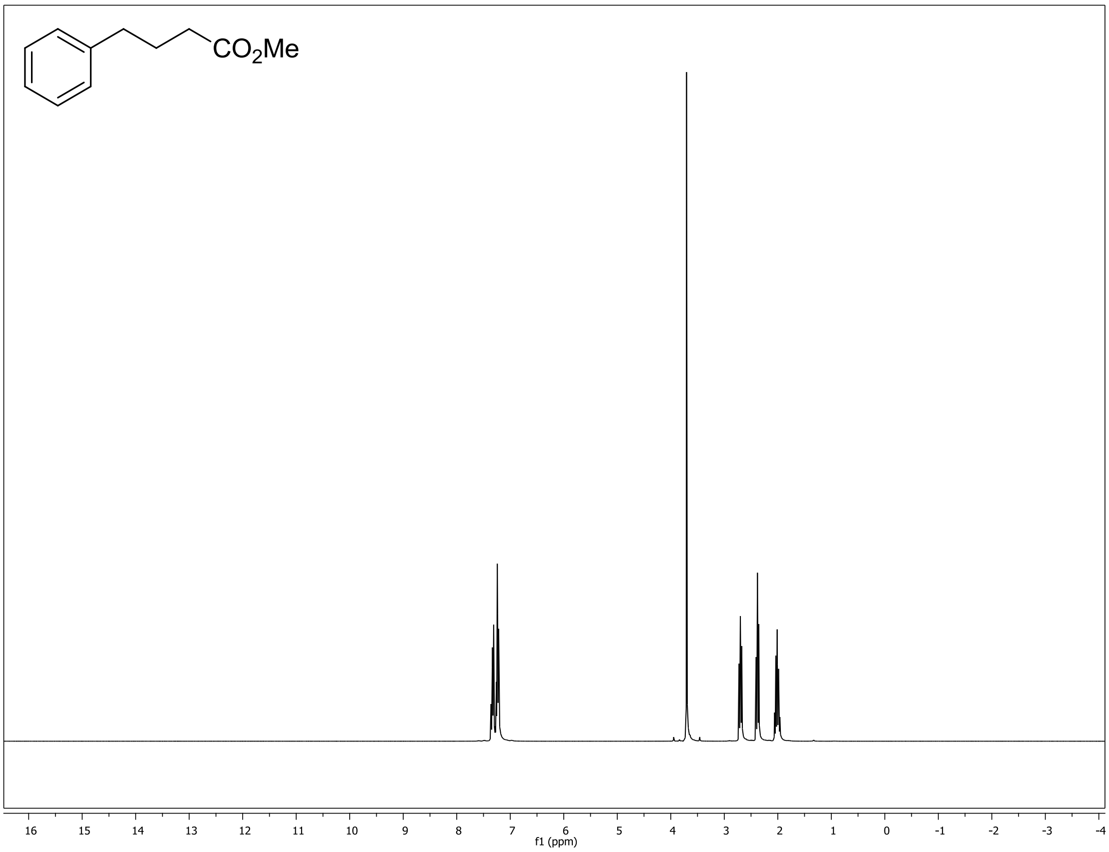
CO2Me
16
15
14
13
12
11
10
9
8
7
6
5
4
3
2
1
0
-1
-2
-3
-4
f1 (ppm)

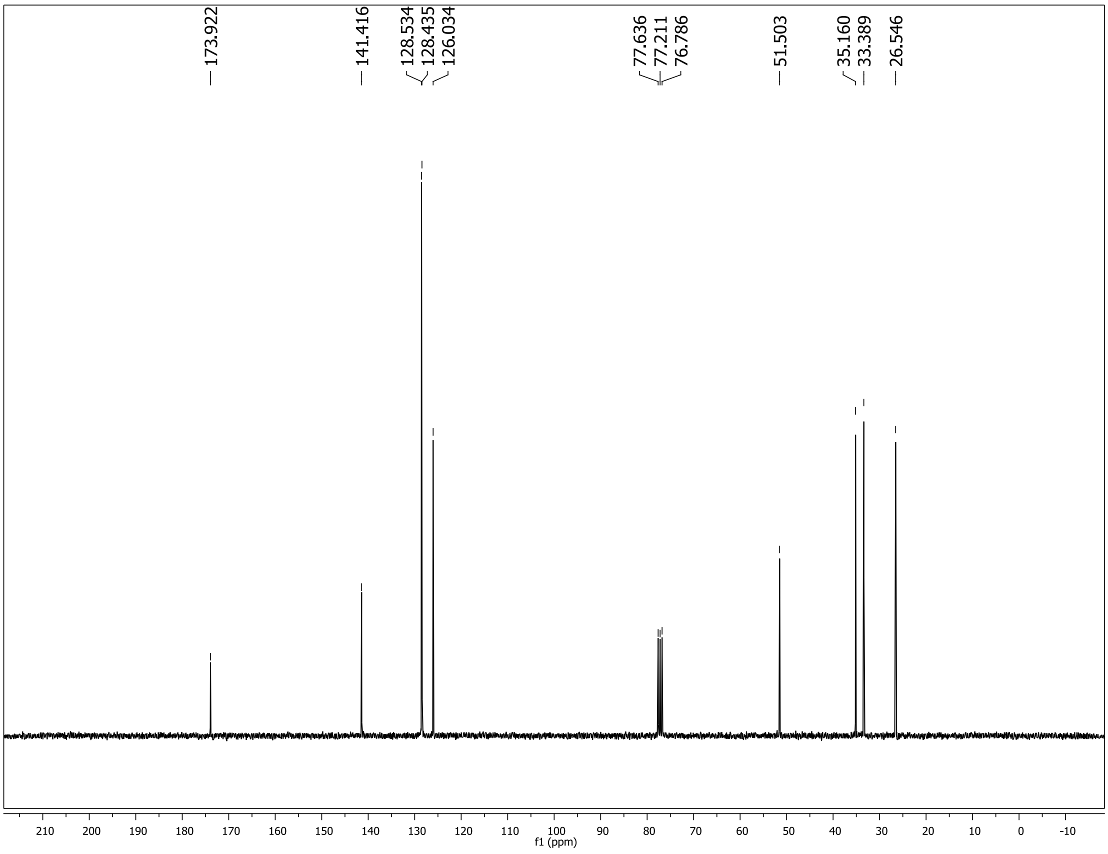
173.922
141.416
128.534
128.435
126.034
77.636
77.211
76.786
51.503
35.160
33.389
26.546
210
200
190
180
170
160
150
140
130
120
110
100
90
80
70
60
50
40
30
20
10
0
-10
f1 (ppm)

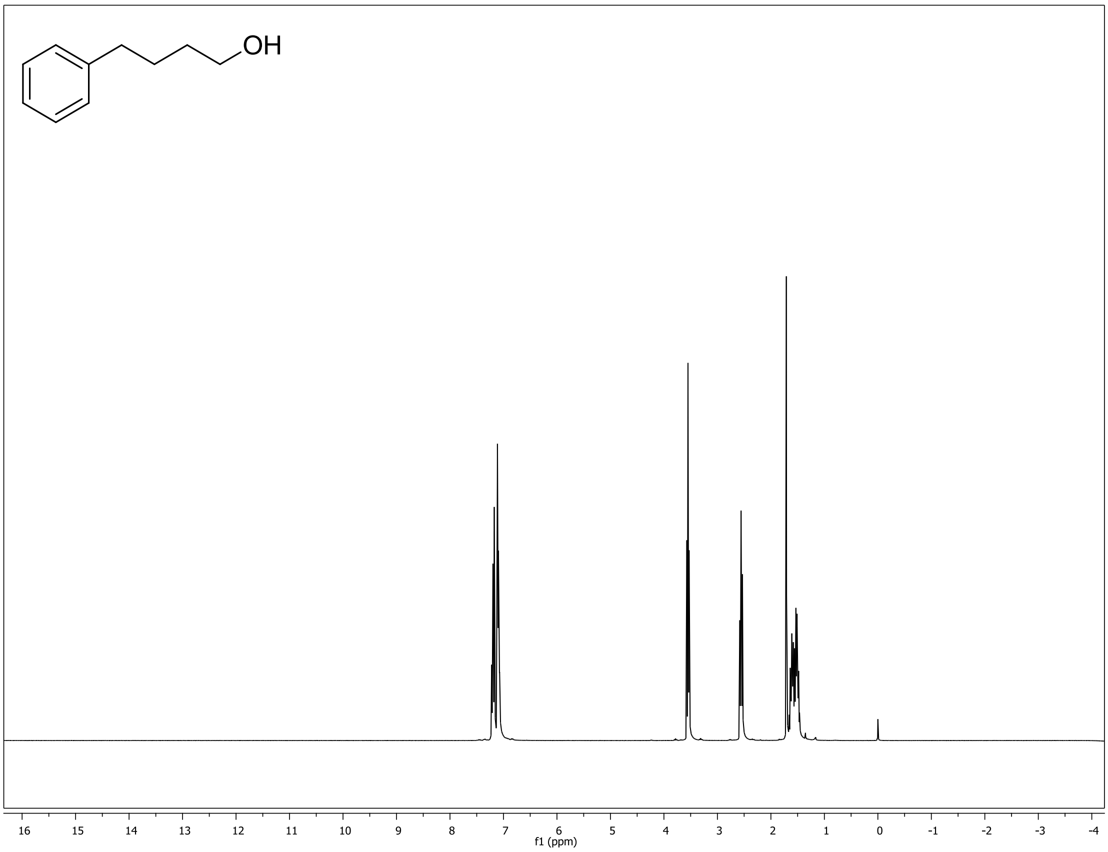
OH
16
15
14
13
12
11
10
9
8
7
6
5
4
3
2
1
0
-1
-2
-3
-4
f1 (ppm)

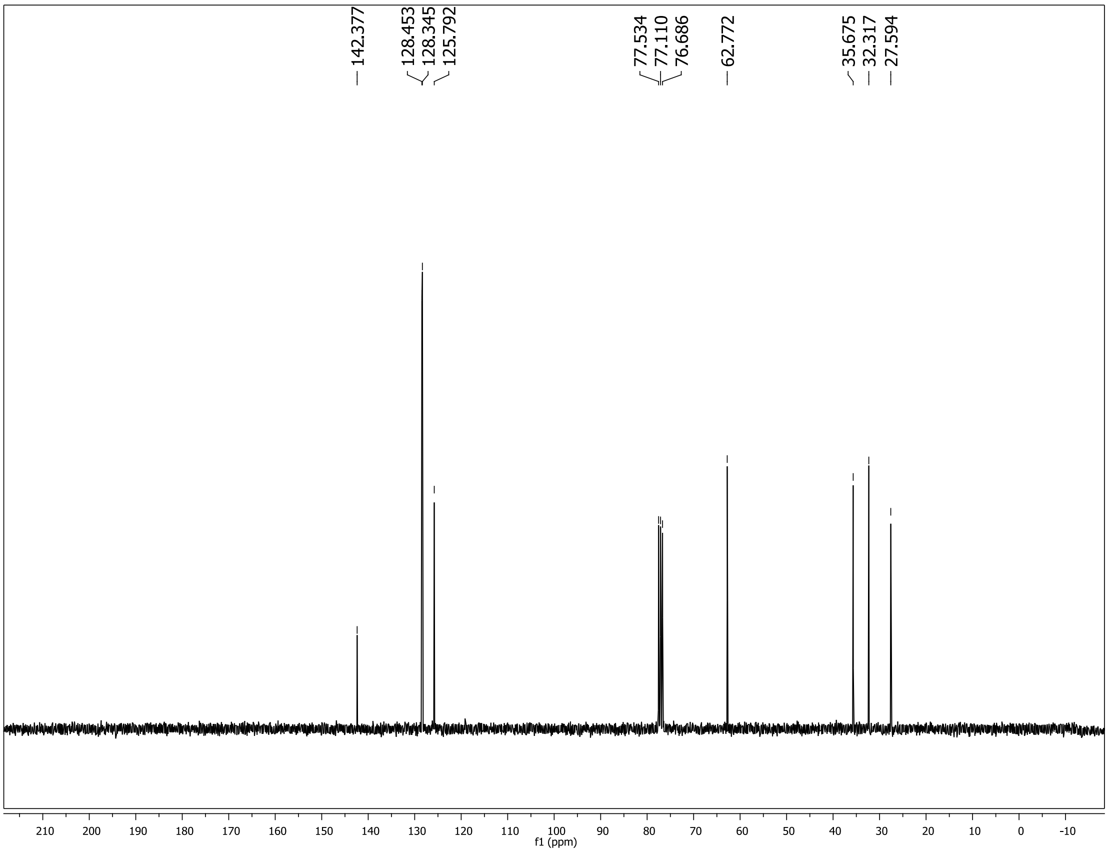
142.377
128.453
128.345
125.792
77.534
77.110
76.686
62.772
35.675
32.317
27.594
210
200
190
180
170
160
150
140
130
120
110
100
90
80
70
60
50
40
30
20
10
0
-10
f1 (ppm)

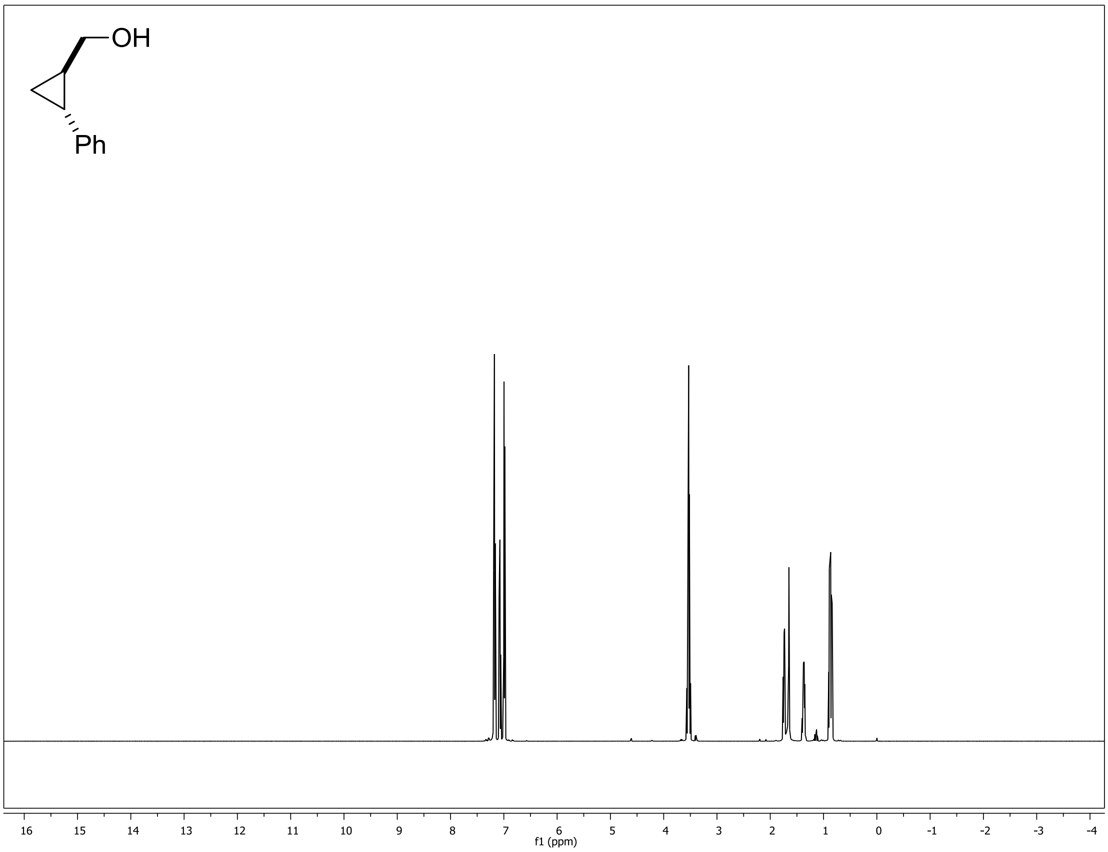
OH
Ph
16
15
14
13
12
11
10
9
8
7
6
5
4
3
2
1
0
-1
-2
-3
-4
f1 (ppm)

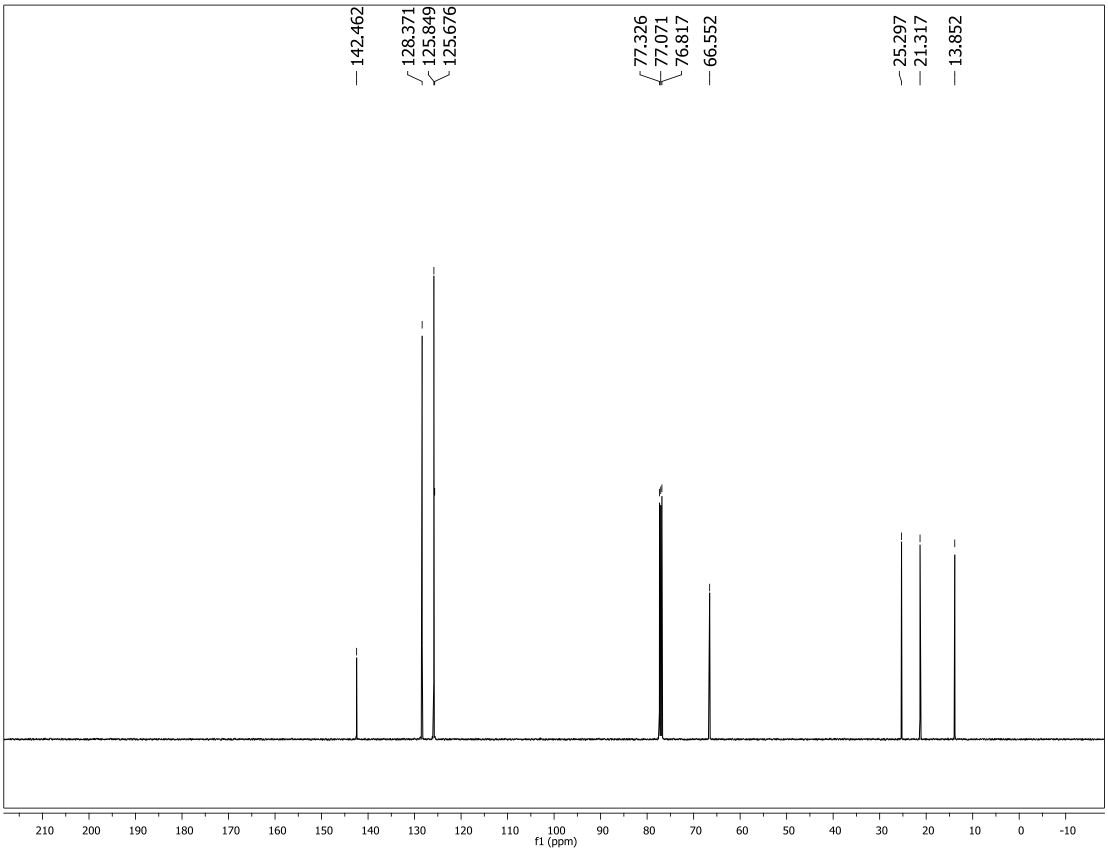
142.462
128.371
125.849
125.676
77.326
77.071
76.817
66.552
25.297
21.317
13.852
210
200
190
180
170
160
150
140
130
120
110
100
90
80
70
60
50
40
30
20
10
0
-10
f1 (ppm)

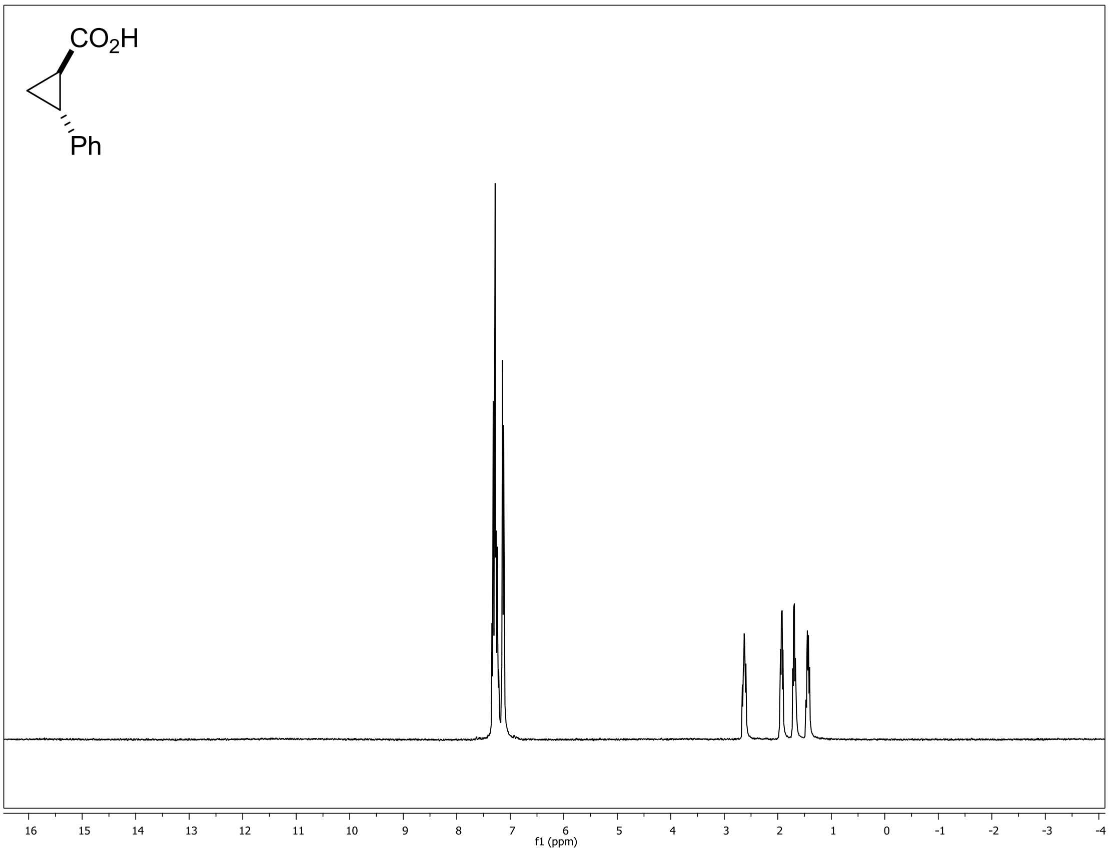
CO2H
Ph
16 15 14 13 12 11 10 9 8 7 6 5 4 3 2 1 0 -1 -2 -3 -4
f1 (ppm)

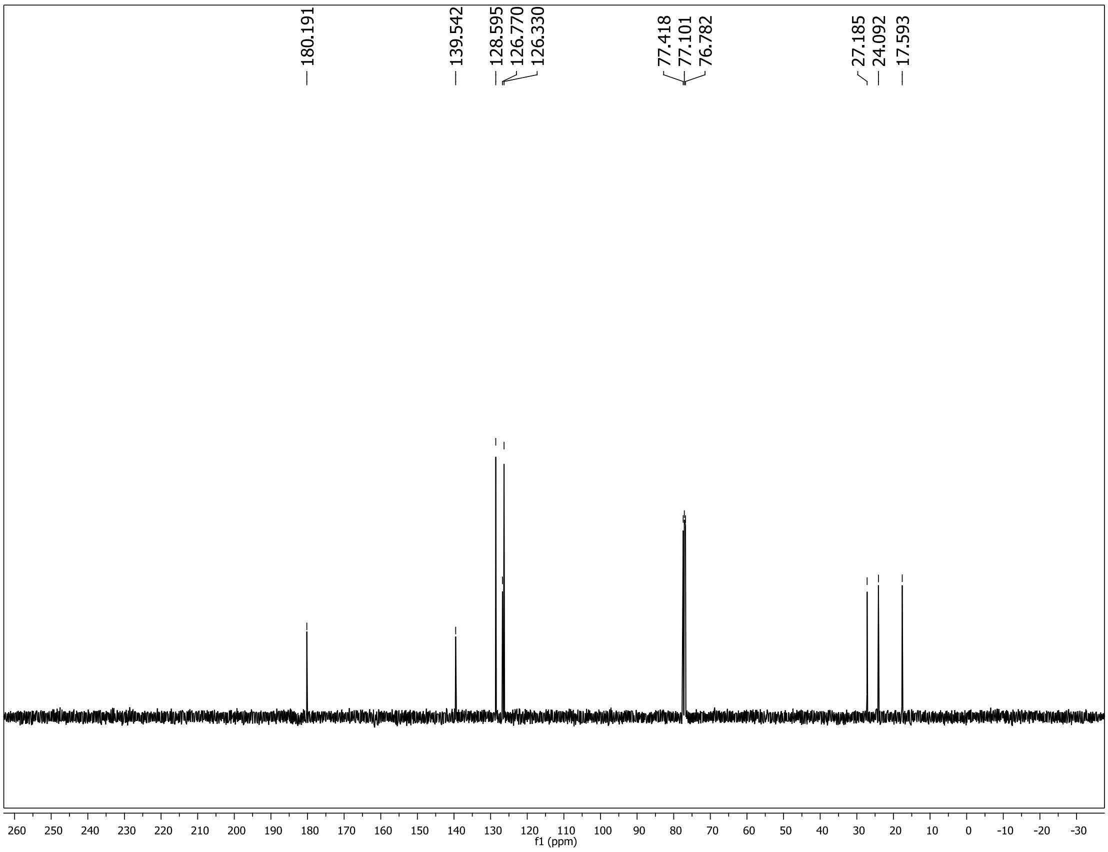
180.191
139.542
128.595
126.770
126.330
77.418
77.101
76.782
27.185
24.092
17.593
260 250 240 230 220 210 200 190 180 170 160 150 140 130 120 110 100 90 80 70 60 50 40 30 20 10 0 -10 -20 -30
f1 (ppm)

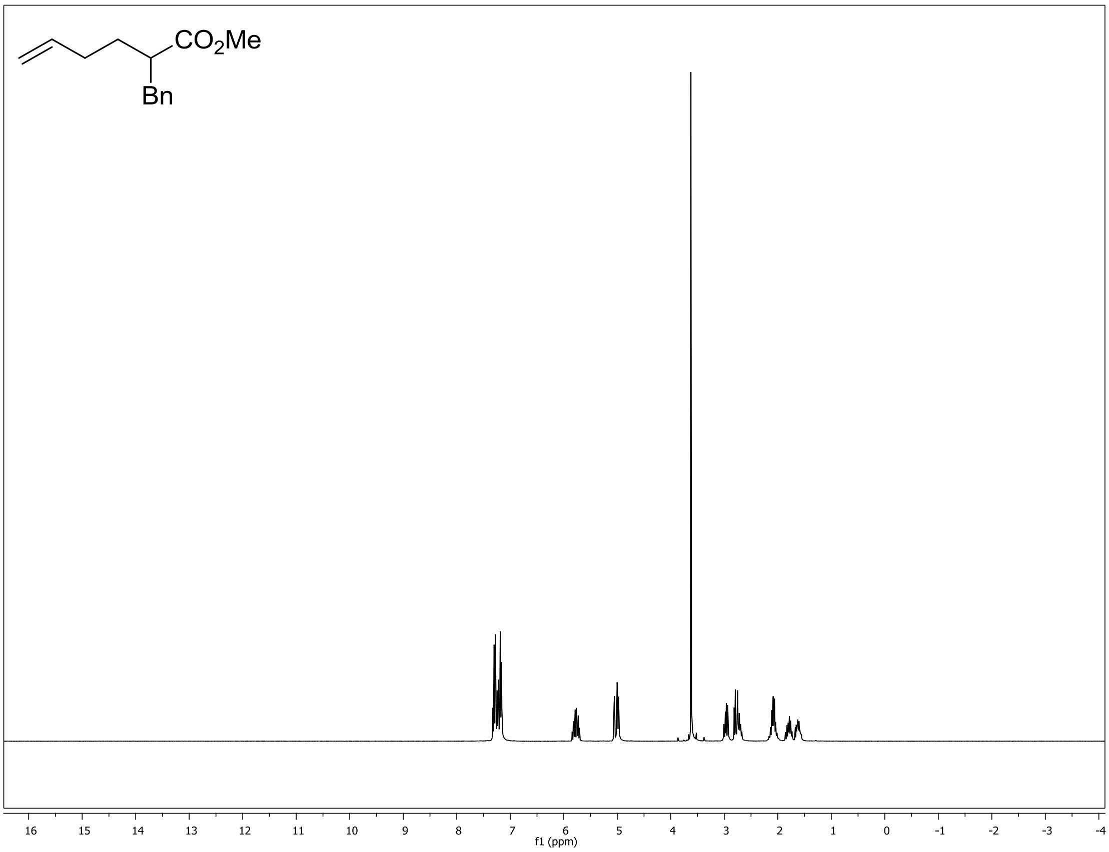
CO2Me
Bn
16 15 14 13 12 11 10 9 8 7 6 5 4 3 2 1 0 -1 -2 -3 -4
f1 (ppm)

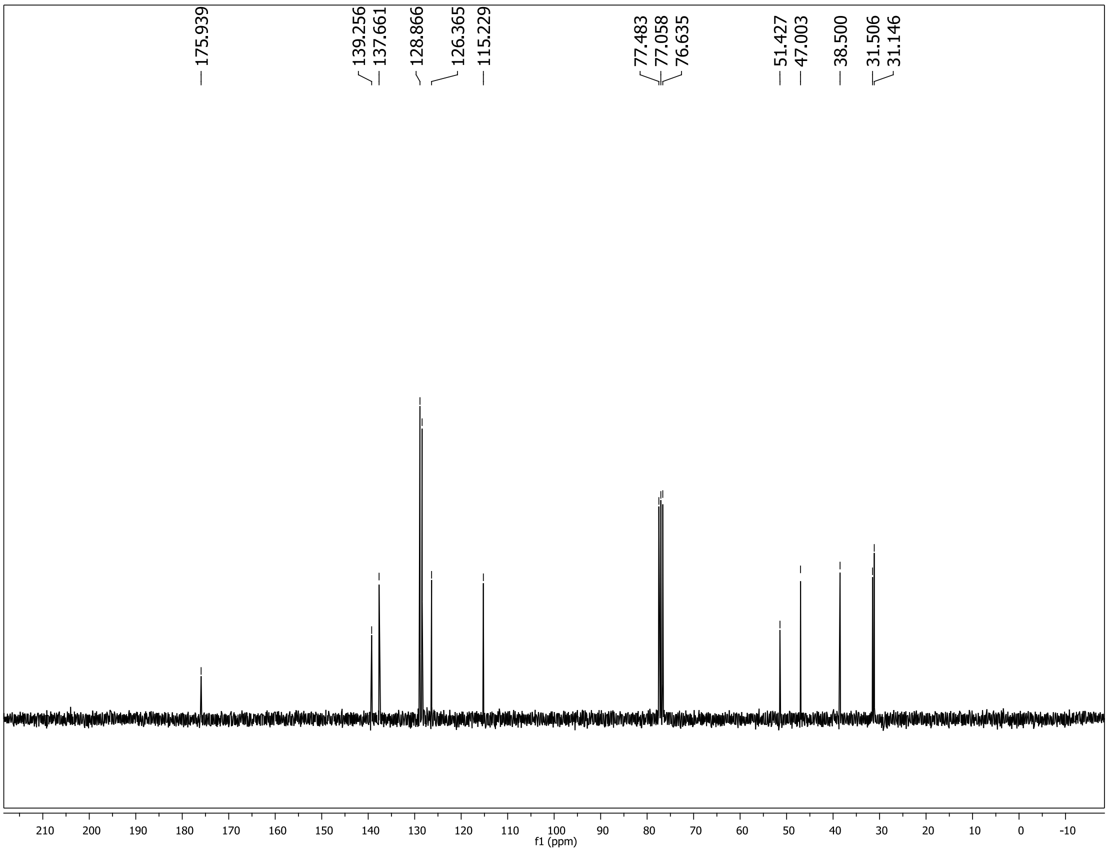
175.939
139.256
137.661
128.866
126.365
115.229
77.483
77.058
76.635
51.427
47.003
38.500
31.506
31.146
210 200 190 180 170 160 150 140 130 120 110 100 90 80 70 60 50 40 30 20 10 0 -10
f1 (ppm)

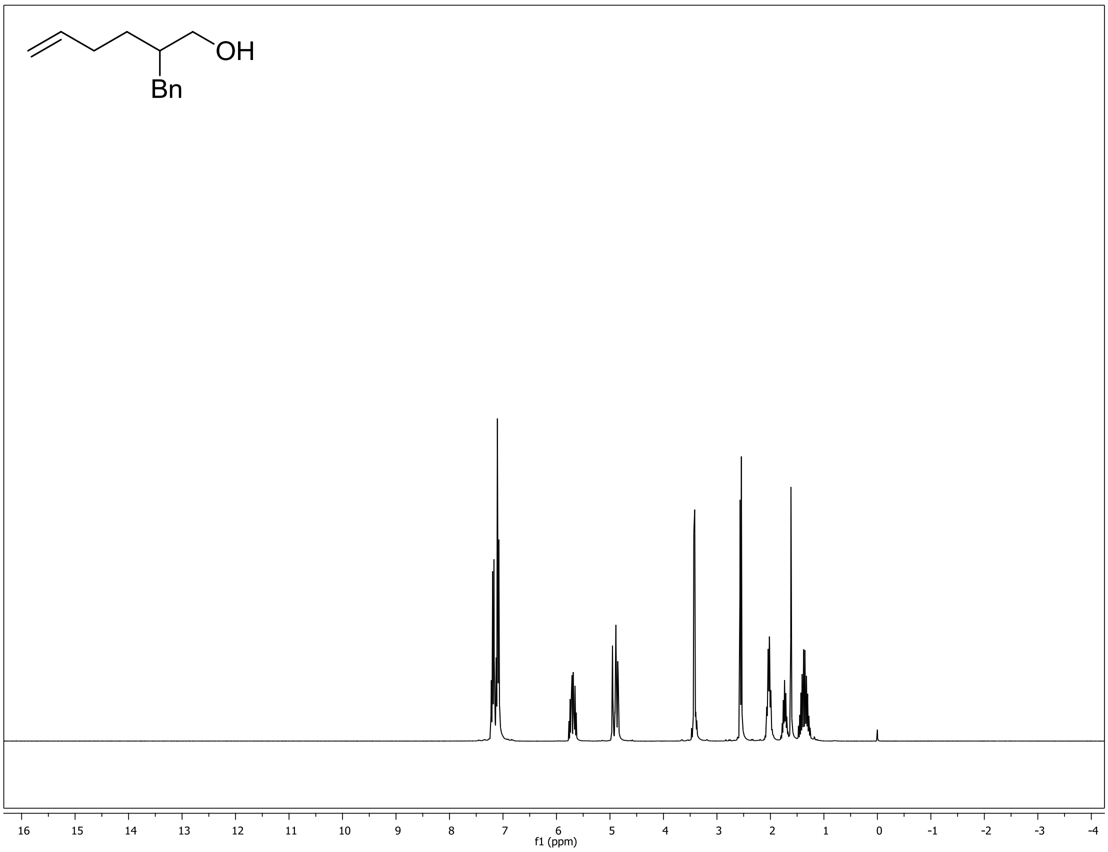
OH
Bn
16
15
14
13
12
11
10
9
8
7
6
5
4
3
2
1
0
-1
-2
-3
-4
f1 (ppm)

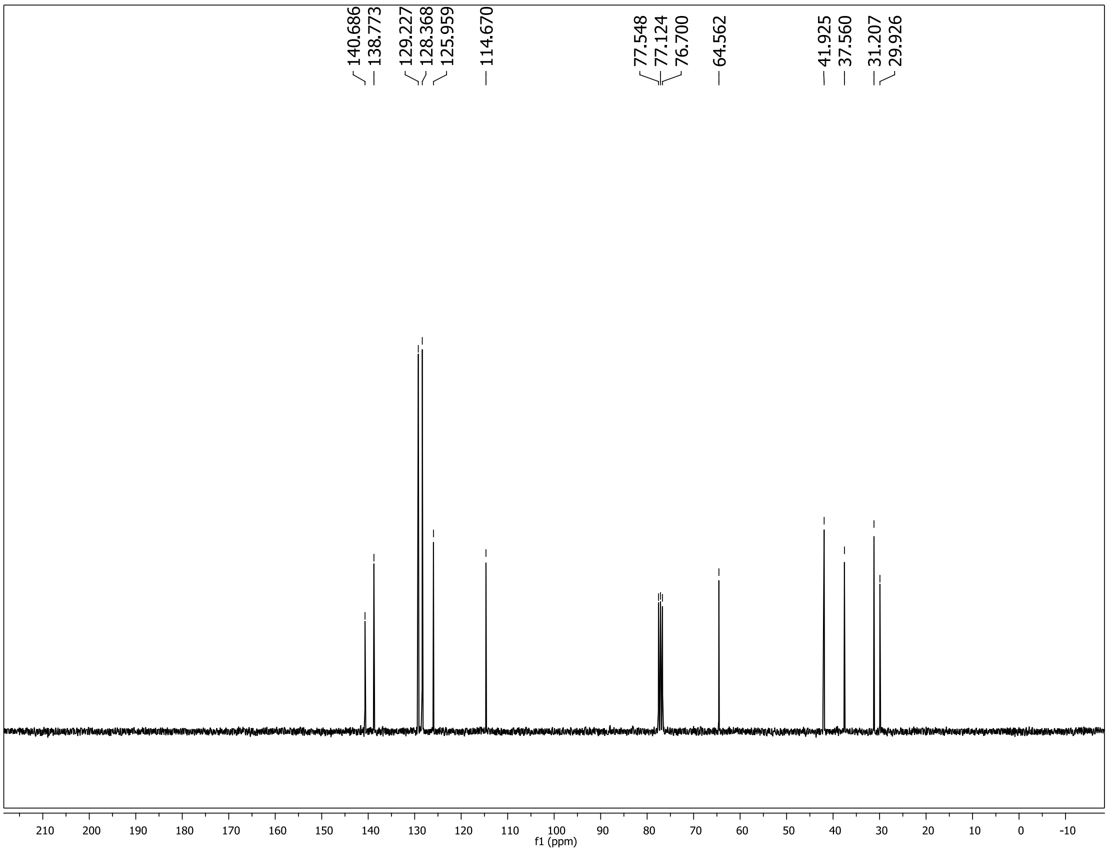
140.686
138.773
129.227
128.368
125.959
114.670
77.548
77.124
76.700
64.562
41.925
37.560
31.207
29.926
210
200
190
180
170
160
150
140
130
120
110
100
90
80
70
60
50
40
30
20
10
0
-10
f1 (ppm)

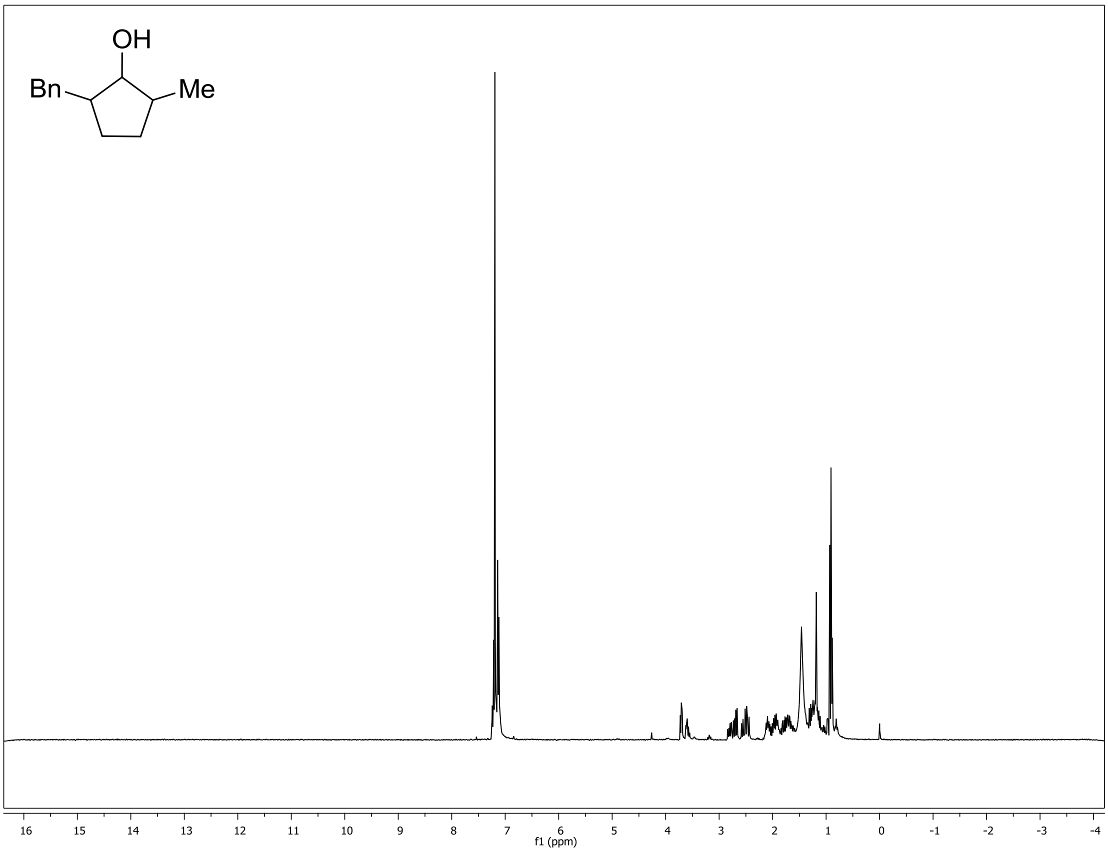
OH
Bn
Me
16 15 14 13 12 11 10 9 8 7 6 5 4 3 2 1 0 -1 -2 -3 -4
f1 (ppm)

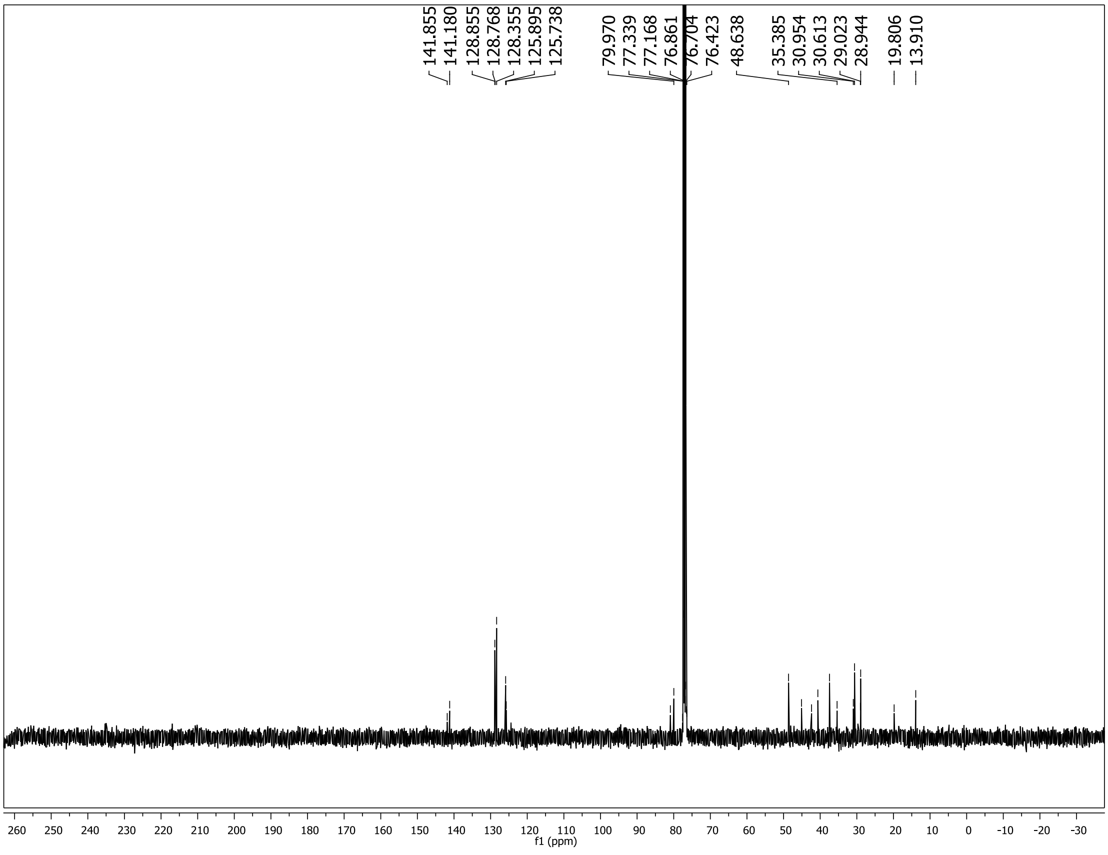
141.855
141.180
128.855
128.768
128.355
125.895
125.738
79.970
77.339
77.168
76.861
76.704
76.423
48.638
35.385
30.954
30.613
29.023
28.944
19.806
13.910
260 250 240 230 220 210 200 190 180 170 160 150 140 130 120 110 100 90 80 70 60 50 40 30 20 10 0 -10 -20 -30
f1 (ppm)

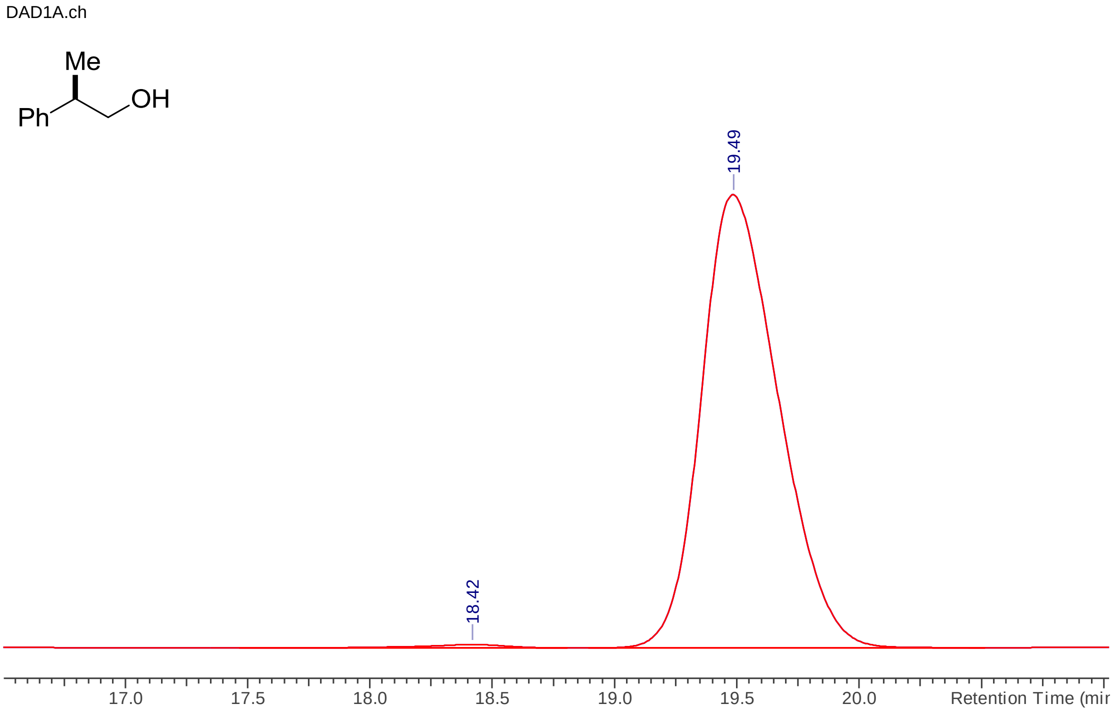

No.	t_R	Peak Area (Y units/ms)	Area Percent	Height
1	18.420	45232.68	0.783	1942.88
2	19.487	5731476.07	99.217	260776.40

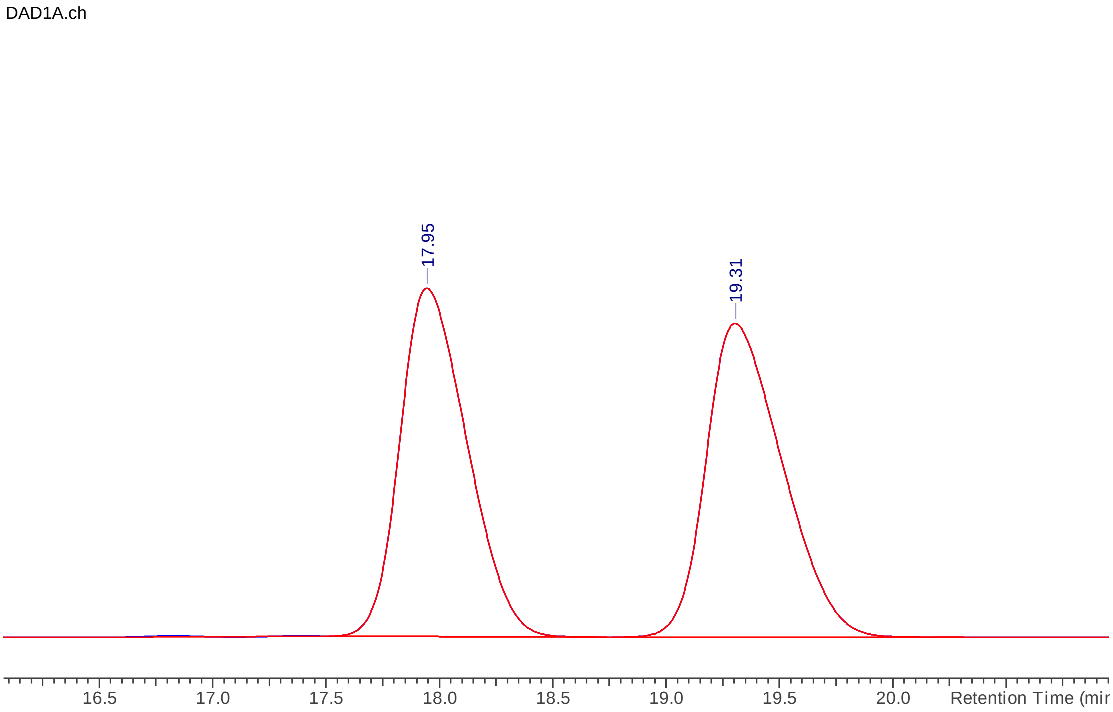

No.	t_R	Peak Area (Y units/ms)	Area Percent	Height
1	17.947	7914426.500	49.575	381518.344
2	19.307	8049978.500	50.425	342846.531
